# Canadian Network for Mood and Anxiety Treatments 2024 Clinical Practice Guideline for the Management of Perinatal Mood, Anxiety, and Related Disorders: Guide de pratique 2024 du Canadian Network for Mood and Anxiety Treatments pour le traitement des troubles de l'humeur, des troubles anxieux et des troubles connexes périnatals

**DOI:** 10.1177/07067437241303031

**Published:** 2025-02-12

**Authors:** Simone N. Vigod, Benicio N. Frey, Crystal T. Clark, Sophie Grigoriadis, Lucy C. Barker, Hilary K. Brown, Jaime Charlebois, Cindy-Lee Dennis, Nichole Fairbrother, Sheryl M. Green, Nicole L. Letourneau, Tim F. Oberlander, Verinder Sharma, Daisy R. Singla, Donna E. Stewart, Patricia Tomasi, Brittany D. Ellington, Cathleen Fleury, Lesley A. Tarasoff, Lianne M. Tomfohr-Madsen, Deborah Da Costa, Serge Beaulieu, Elisa Brietzke, Sidney H. Kennedy, Raymond W. Lam, Roumen V. Milev, Sagar V. Parikh, Arun V. Ravindran, Zainab Samaan, Ayal Schaffer, Valerie H. Taylor, Smadar V. Tourjman, Michael Van Ameringen, Lakshmi N. Yatham, Ryan J. Van Lieshout

**Affiliations:** 1 7985 Department of Psychiatry and Women's College Research and Innovation Institute, Women's College Hospital, Toronto, ON, Canada; 2 Department of Psychiatry, 12366 Temerty Faculty of Medicine, 7938 University of Toronto, Toronto, ON, Canada; 3 Institute for Health Policy, Management and Evaluation, 274071 Dalla Lana School of Public Health, 7938 University of Toronto, Toronto, ON, Canada; 4 Department of Psychiatry and Behavioural Neurosciences, 3710 McMaster University, Hamilton ON, Canada; 5 Women's Health Concerns Clinic, 25479 St. Joseph's Healthcare Hamilton, Hamilton, ON, Canada; 6 71545 Department of Psychiatry, Sunnybrook Health Sciences Centre, Toronto, ON, Canada; 7 Department of Health and Society, 33530 University of Toronto, Scarborough, ON, Canada; 8 25463 Waypoint Centre for Mental Health Care, Penetanguishene, ON, Canada; 9 Lawrence Bloomberg Faculty of Nursing, 7938 University of Toronto, Toronto, ON, Canada; 10 90755 Lunenfeld-Tanenbaum Research Institute, Mount Sinai Hospital, Toronto, ON, Canada; 11 Department of Family Practice, 8166 University of British Columbia, Vancouver, BC, Canada; 12 120922 Michael Smith Foundation for Health Research, Vancouver, BC, Canada; 13 Faculty of Nursing and Cumming School of Medicine, 8166 University of Calgary; 14 Department of Psychiatry, 8166 University of British Columbia, Vancouver, BC, Canada; 15 Department of Pediatrics, 120479 University of British Columbia, Vancouver, BC, Canada; 16 School of Population and Public Health, 8166 University of British Columbia, Vancouver, BC, Canada; 17 BC Children's Hospital Research Institute, 8166 University of British Columbia, Vancouver, BC, Canada; 18 Department of Psychiatry, 6221 Western University, London, ON, Canada; 19 Department of Obstetrics and Gynecology, 6221 Western University, London, ON, Canada; 20 Campbell Family Mental Health Research Institute, 7978 Centre for Addiction and Mental Health, Toronto, ON, Canada; 21 Toronto General Hospital Research Institute, 7989 Centre for Mental Health, University Health Network, Toronto, ON, Canada; 22 Canadian Perinatal Mental Health Collaborative, Barrie, ON, Canada; 23 Department of Educational and Counselling Psychology, and Special Education, 8166 University of British Columbia, Vancouver, BC, Canada; 24 Department of Medicine, 5620 McGill University, Montreal, QC, Canada; 25 Centre for Outcomes Research and Evaluation, 507266 Research Institute of the McGill University Health Centre, Montreal, QC, Canada; 26 Department of Psychiatry, 5620 McGill University, Montreal, QC, Canada; 27 Department of Psychiatry, 4257 Queen's University and Providence Care Hospital, Kingston, ON, Canada; 28 Department of Psychiatry, 1259 University of Michigan, Ann Arbour, MI, USA; 29 Department of Psychiatry, 2129 University of Calgary, Calgary, AB, Canada; 30 Department of Psychiatry, 26612 Montreal Institute of Mental Health, Université de Montréal, Montréal, QC, Canada

**Keywords:** clinical practice guidelines, depression, postpartum depression, mood disorder, anxiety disorder, obsessive–compulsive disorder, post-traumatic stress disorder, bipolar disorder, perinatal, postpartum psychosis, pregnancy, postpartum period

## Abstract

**Background:**

The Canadian Network for Mood and Anxiety Treatments (CANMAT) publishes clinical practice guidelines for mood and anxiety disorders. This CANMAT guideline aims to provide comprehensive clinical guidance for the pregnancy and postpartum (perinatal) management of mood, anxiety and related disorders.

**Methods:**

CANMAT convened a core editorial group of interdisciplinary academic clinicians and persons with lived experience (PWLE), and 3 advisory panels of PWLE and perinatal health and perinatal mental health clinicians. We searched for systematic reviews of prevention and treatment interventions for perinatal depressive, bipolar, anxiety, obsessive–compulsive and post-traumatic stress disorders (January 2013–October 2023). We prioritized evidence from reviews of randomized controlled trials (RCTs), except for the perinatal safety of medications where reviews of large high-quality observational studies were prioritized due to the absence of RCT data. Targeted searches for individual studies were conducted when systematic reviews were limited or absent. Recommendations were organized by lines of treatment based on CANMAT-defined levels of evidence quality, supplemented by editorial group consensus to balance efficacy, safety, tolerability and feasibility considerations.

**Results:**

The guideline covers 10 clinical sections in a question-and-answer format that maps onto the patient care journey: case identification; organization and delivery of care; non-pharmacological (lifestyle, psychosocial, psychological), pharmacological, neuromodulation and complementary and alternative medicine interventions; high-risk clinical situations; and mental health of the father or co-parent. Equity, diversity and inclusion considerations are provided.

**Conclusions:**

This guideline's detailed evidence-based recommendations provide clinicians with key information to promote the delivery of effective and safe perinatal mental healthcare. It is hoped that the guideline will serve as a valuable tool for clinicians in Canada and around the world to help optimize clinical outcomes in the area of perinatal mental health.

**Plain Language Summary Title:**

The Canadian Network for Mood and Anxiety Treatments 2024 Guideline for Helping People with Mood, Anxiety and Related Disorders During Pregnancy and Postpartum

## Plain Language Summary

The Canadian Network for Mood and Anxiety Treatments publishes guidelines for healthcare providers, to aid them in staying up-to-date with the latest and best evidence around treatments for mood and anxiety problems. This is the first time that CANMAT is publishing a guideline that focuses specifically on mental health during pregnancy and postpartum (the perinatal period). The guideline primarily focuses on treatment recommendations, including both medication and non-medication options. It explains when and how to use these treatments to make sure they are as effective as possible. Importantly, the guideline was developed in close collaboration with a diverse group of people, including people who have experience with mood and anxiety problems during the perinatal period, as well as clinicians from various fields of expertise. In creating this guideline, we reviewed scientific papers that combine results from multiple studies and considered the quality of the research when making recommendations. We also aimed to include equity, diversity and inclusion in our considerations. We hope that this clinical guideline will be a helpful tool for healthcare providers to improve care for people experiencing mental health issues during pregnancy and after childbirth.

## Résumé

**Avant-propos:**

Le Canadian Network for Mood and Anxiety Treatments (CANMAT) publie des guides de pratique pour les troubles de l'humeur et les troubles anxieux. Le présent guide du CANMAT vise à offrir des lignes directrices complètes pour la prise en charge pendant la grossesse et le post-partum (périnatal) des troubles de l'humeur, de l'anxiété et des troubles connexes.

**Méthodes:**

Le CANMAT a réuni un groupe de rédaction interdisciplinaire composé de cliniciens du monde universitaire, des personnes ayant un vécu expérientiel (PAVE) et 3 comités consultatifs formés de PAVE, de cliniciens spécialisés en santé périnatale et de cliniciens spécialisés en santé mentale périnatale. Nous avons recherché des revues systématiques portant sur des interventions préventives et thérapeutiques pour les troubles suivants en période périnatale : troubles dépressifs, bipolaires, anxieux, obsessionnels-compulsifs et de stress post-traumatique (janvier à octobre 2023). Nous avons privilégié les données probantes issues des revues d'essais contrôlés randomisés (ECR), à l'exception de notre évaluation de l'innocuité périnatale des médicaments, où nous avons donné la priorité aux synthèses d'études observationnelles de grande envergure et de haute qualité en raison de l'absence de données issues d'ECR. Des recherches ciblées d'études individuelles ont été effectuées lorsque les synthèses systématiques étaient limitées ou inexistantes. Les recommandations ont été organisées par lignes de traitement sur la base des critères établis par le CANMAT pour la qualité des données, ainsi qu'en nous appuyant sur le consensus du groupe de rédaction pour assurer l'équilibre de diverses considérations, notamment l'efﬁcacité, l'innocuité, la tolérabilité et la faisabilité.

**Résultats:**

Ce guide comporte 10 sections cliniques sous forme de questions-réponses qui suivent le parcours de soins du patient : dépistage; organisation et prestation des soins; approches non pharmacologiques (psychosociales, psychologiques ou liées au mode de vie), pharmacothérapies, neuromodulation, interventions complémentaires et alternatives; situations cliniques à risque élevé; et santé mentale du père ou de la partenaire. Le guide tient compte des principes d'équité, de diversité et d'inclusion.

**Conclusions:**

Les recommandations détaillées de ce guide, fondées sur des données probantes fournissent aux cliniciens des renseignements pertinents pour promouvoir la prestation de soins de santé mentale qui sont sûrs et efficaces en période périnatale. Nous espérons que cette ligne directrice s'avérera un outil précieux pour les cliniciens du Canada et du monde entier afin d'optimiser les résultats cliniques dans le domaine de la santé mentale périnatale.

## Introduction

Mood, anxiety and related disorders are some of the most common conditions that arise in pregnancy and in the year after childbirth.^1–3^ When untreated or undertreated, perinatal mood, anxiety and related disorders (PMADs) can negatively impact the well-being and quality of life of affected persons, and the health and development of their children and families.^3,4^ This makes the identification and management of PMADs a public health priority.

Clinical practice guidelines can synthesize best practices to help guide the delivery of timely, effective, high-quality care.^5^ The Canadian Network for Mood and Anxiety Treatments (CANMAT) is a not-for-profit scientific and educational organization that produces clinical guidelines outlining the latest research and treatment options for managing mood, anxiety and related disorders (www.canmat.org). Previous CANMAT guidelines for major depressive disorder (MDD) and bipolar disorder (BD) included sections on perinatal populations.^6,7^ This PMAD-specific CANMAT guideline aims to provide a more comprehensive guidance on best practices in care and engage a target audience beyond psychiatrists to include all clinicians (e.g., family physicians, midwives, nurses, obstetricians, pediatricians, psychotherapists, social workers and others) who may require this knowledge for case identification, referral and/or management.

This guideline summarizes the evidence for interventions to prevent and treat PMADs, including specifically MDD, anxiety disorders, obsessive–compulsive disorders (OCD), post-traumatic stress disorder (PTSD) and BD in pregnancy and up to 1 year postpartum. Postpartum psychosis, a severe postpartum clinical presentation that is strongly associated with bipolar disorder, is also included. The guideline also covers clinical presentations of mood, anxiety and related disorders that are unique to the perinatal period such as fear of childbirth and symptoms of PTSD that arise in relation to experiences of trauma around the time of birth, sometimes termed childbirth-related PTSD (CB-PTSD). While other types of mental disorders also affect people in pregnancy and/or postpartum—on their own or comorbid with PMADs (e.g., autism, attention-deficit-hyperactivity disorder, eating disorders, personality disorders, substance and alcohol use disorders and others)—they were considered to be outside the scope of this initial guideline.

Throughout the guideline, we strive to use language inclusive of women and gender-diverse childbearing people and their partners, including those who are transgender, non-binary and two-spirit.^8,9^ We aimed to include evidence related to equity, diversity and inclusion in each section of the guideline, focusing on specific considerations related to age, sexual and gender identity, race, ethnicity, culture and disability.

### Key Caveats

There are some key caveats to consider when reading this guideline. First, while the first year postpartum is a conventional cut-off point in the literature, many PMADs continue beyond this period. The evidence and recommendations in this guideline may still be applicable after the first year postpartum, especially when considering issues related to lactation and interventions adapted to focus on parenting or the maternal-child dyad. Second, in the PMAD literature, many studies utilize symptom scales, and not diagnostic interviews for study inclusion. While symptom scales correlate highly with diagnostic interviews, it is difficult to differentiate in some cases whether the results of such research would apply to those with diagnosed PMADs specifically.^10^ Many individuals with PMAD symptoms—even when they do not meet diagnostic criteria for a mental disorder per se—may benefit from treatment, but throughout the guideline we aim to distinguish between interventions evaluated in populations with elevated symptoms versus those with diagnosed disorders when possible. Third, while the guideline is meant to assist clinicians in managing PMADs using an evidence-based approach, it is not meant to replace clinical judgement nor is it a legal or policy care standard. Clinicians and decision-makers will want to consider the recommendations in the context of their own settings (e.g., availability of specific psychotherapies, medication and/or other treatments) and their local regulations of clinical practice. Fourth, the guideline evidence review is updated to October 2023, so clinicians are encouraged to keep up to date with the latest research so as to provide their patients with the best possible care. Finally, the guideline is written in technical, academic language, with the target audience being healthcare professionals. This is not necessarily easily accessible for patients or families who are trying to decipher the guidelines for their own personal educational needs, so there will be an accompanying Patient and Family Guide.

## Methods

The development of the guideline followed the structured methodology of previous CANMAT guidelines.^7,11^ The project was led by a core editorial group of 15 PMAD experts spanning psychiatry, psychology, pediatrics, nursing and public health, and 2 persons with lived experience (PWLE). Meetings of the core group were biweekly by video-conferencing for the duration of the project (April 2023 to October 2024), supplemented by email communication. The project received input from the CANMAT Board and 3 advisory panels comprising 27 individuals with diverse experiences and backgrounds from across Canada: (a) PWLE (*n* = 10), (b) non-mental health clinical experts, including midwifery, obstetrics and gynecology, nursing, pediatrics and primary care (*n* = 8) and (c) perinatal mental health clinicians (*n* = 9) (Supplement 1. Advisory Panels). Advisory panel members were engaged for input and feedback through structured surveys, email communication, focus groups and 1:1 discussions with the co-leads and other members of the project team.

### Scope and Structure

The current guideline follows a question-and-answer format, with a focus on clinical questions relevant to the management of PMADs (Table 1). The questions were initially drafted by the core editorial group and CANMAT Board, then revised based on feedback from the advisory panels. Advisory panel members were asked to provide input on the nature and importance of the questions, how they were worded, and the rank order in which questions should be presented. They were also asked their perspective on whether there were any key clinical questions missing and the extent to which equity, diversity and inclusion were addressed. Feedback was reviewed and incorporated by the core editorial group, who approved the final set of questions by consensus agreement. The final questions were then shared back to the advisory panel members.

**Table 1.** Guideline Sections and Questions.

Epidemiology and Impact
Foundations of Clinical Management
Initial Diagnostic Assessment
Diagnostic Considerations
General Treatment Considerations
What are the recommendations for the:
1. Identification of perinatal mood, anxiety and related disorders?
2. Organization and delivery of perinatal mental healthcare services?
3. Lifestyle interventions for the prevention and treatment of PMADs?
4. Use of psychosocial interventions in the prevention and treatment of PMADs?
5. Use of psychological interventions in the prevention and treatment of PMADs?
6. Use of pharmacological interventions in pregnancy and lactation?
7. Use of neuromodulation interventions in the prevention and treatment of PMADs?
8. Use of complementary and alternative treatments for the prevention and treatment of PMADs?
9. Management of high-risk clinical situations?
10. Identification, prevention and treatment of PMADs in fathers and co-parents?

*Note*. PMADs = perinatal mood, anxiety and related disorders.

### Evidence Review

PubMed, EMBASE, PsycINFO, MEDLINE, CINAHL, Cochrane and Web of Science databases were searched for English- and French-language systematic reviews and meta-analyses published from 1 January 2013 to 29 October 2023. A list of key terms and subject headings were developed by the team, and refined by a medical librarian, according to 2 concepts: (a) mood, anxiety and related disorders and (b) the perinatal life stage. Large well-conducted randomized controlled trials (RCTs) are the highest standard of evidence, so we prioritized reviews that synthesized the results of RCTs in the current guidelines. In the absence of systematic reviews of RCTs (e.g., for medication safety in pregnancy), systematic reviews of large observational studies were included. Targeted searches for single RCTs and other studies were conducted when systematic review data were limited or absent (Supplement 2. Search Strategy).

After removing duplicates, 12,718 articles were identified for title and abstract screening, 733 underwent full-text review and 470 systematic reviews of interventions (RCTs, or observational studies in the setting of medications in pregnancy only) were identified (Supplement 2). Due to a paucity of systematic reviews relevant to BD, a targeted search on case identification and pharmacotherapy was conducted, resulting in an additional 89 studies. During study selection, articles on epidemiology and key foundations of management not explicitly covered in the 10 guideline questions and other clinical practice guidelines were flagged as potential reference documents, but not included for data extraction or quality appraisal (*N* = 942). Study selection was completed by 2 independent reviewers, with a third reviewer to adjudicate conflicts.

Data extraction and quality appraisal were conducted in 2 stages. First, to prioritize the most contemporary evidence, data from included studies published in the 5 years prior to the end of the search date (2018–2023) were extracted and appraised. If the reviews published in the 5 years prior to the search date did not provide adequate data for recommendations to be made, data were extracted and appraised from the remaining reviews. In the end, this was done only for Question 9. In total, data were extracted from 298 of the studies included in the search using a standardized data extraction form based on the Template for Intervention Description and Replication checklist. Quality appraisal was based on the Grading of Recommendations, Assessment, Development and Evaluations framework.^12,13^

After data extraction and quality appraisal, the authors assigned a “Level of Evidence” to each type of prevention or treatment intervention based on study design, size and quality for the totality of the evidence, as defined in previous CANMAT guidelines (Table 2). The section leads of the given question/section (3 to 5 authors/section) reviewed the quality appraisal of the articles pertaining to their specific sections and assigned the Level of Evidence based on the criteria in Table 2. Any discordance was resolved through input of the co-leads and additional authors when necessary to reach a consensus decision. In this guideline, green circles are used to indicate that there is positive empirical evidence that the intervention is effective, and red squares are used when there is empirical evidence that the intervention is not effective. Blue circles are used to represent the strength and quality of the evidence on safety of the intervention in pregnancy and/or lactation. The blue circles do not indicate whether the safety profile is favourable or not, only the Level of Evidence available.

**Table 2.** CANMAT Criteria for Level of Evidence.

Level of evidence	Symbol			Criteria
	Efficacy		Perinatal safety	
	Positive	Negative		
1	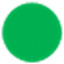	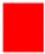	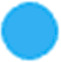	High-quality meta-analyses with narrow confidence intervals or replicated RCTs with adequate sample size
2	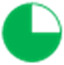	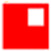	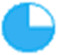	Lower-quality meta-analyses with wide confidence intervals and/or one or more RCTs with adequate sample size
3	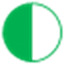	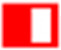	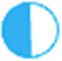	At least 1 small-sample RCT or high-quality, controlled observational studies
4	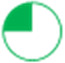	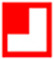	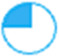	Pilot studies, uncontrolled trials, anecdotal reports or expert consensus opinion

*Note*. For Level 1 and Level 2, RCTs are required (adequate sample size is at least *n* ≥ 30 per treatment arm). Recommendations from epidemiological data primarily arise from observational studies, where the highest level of evidence is usually Level 3. Higher order recommendations (e.g., principles of care) reflect higher level judgement of strength of evidence from various data sources and therefore are primarily Level 3 or 4 evidence. RCT = randomized controlled trial.

### Recommendation Development Process

Each core editorial group member and CANMAT board member co-author was assigned to one or more of the guideline questions. Recommendations were developed based on the level of evidence available for efficacy and clinical support for the intervention, namely the balance between efficacy, safety, tolerability and the feasibility of applying the intervention perinatally (Table 3). For example, a treatment with Level 1 evidence for efficacy could be recommended as a second- or third-line treatment option (or not recommended) due to concern about its safety, side effect profile or feasibility of its use. In addition, as there are no RCTs of medications in pregnancy (and few in the postpartum), Level 3 and Level 4 perinatal-specific evidence on efficacy, safety and tolerability of medications was combined with efficacy and tolerability evidence from non-perinatal populations to inform the recommendations. For example, a medication with Level 1 evidence for efficacy non-perinatally for which there is also high-quality reassuring Level 3 evidence for safety in perinatal populations could be considered a first-line treatment even in the absence of perinatal-specific efficacy data. In contrast, a medication with Level 1 evidence for efficacy in non-perinatal populations but with high-quality Level 3 evidence that there are safety concerns in perinatal populations could be downgraded to second-, third-line or even “not recommended” depending on the degree/severity of the safety concern.

**Table 3.** CANMAT Criteria for Line of Treatment.

Line of treatment	Criteria^a^
First-line	Level 1 or Level 2 evidence plus clinical support
Second-line	Level 3 evidence or higher plus clinical support
Third-line	Level 4 evidence or higher plus clinical support
Not recommended	Level 1 or 2 evidence for lack of efficacy or safety concerns, plus clinical support

*Note*. CANMAT = Canadian Network for Mood and Anxiety Treatments.

^a^ Clinical support reflects the authors' expert opinion/consensus on the relevance of the evidence on safety, efficacy and feasibility of the intervention. For pharmacological treatment recommendations, due to the paucity of perinatal-specific randomized controlled trials, Level 3 or 4 evidence on efficacy and safety in perinatal-specific populations might be considered sufficient for a first-line or second-line recommendation in the presence of Level 1 or 2 evidence for efficacy in non-perinatal populations.

Preliminary recommendations were presented to the entire authorship group in a full-day meeting in March 2024 for initial feedback (in-person, with video-conferencing options for those unable to travel to the meeting), followed by ongoing discussion and consensus meetings within Question groups, along with the drafting of text to accompany the recommendations. The 2 project co-leads (SV, BF) then reviewed and revised each section. Draft recommendations and a full draft of the guideline were completed in June 2024. The advisory panels were then re-engaged to provide feedback on the recommendations and their explanations as well as the tone, language and completeness of the guideline. A final draft of the guideline was approved by the authorship group for submission for peer review on September 13, 2024.

## Epidemiology and Impact

The prevalence of depression at any point in the perinatal period ranges from 6.5 to 12.9%, with a lower rate when major depression is confirmed by structured clinical interviews.^14^ There is a particularly high prevalence of depression in the postpartum period, estimated at up to 19% for clinically significant depressive symptoms, and 7% for a major depressive episode (MDE).^14,15^ The risk for postpartum depression (PPD) is usually highest within the first 6 months of childbirth. This is inconsistent with the more narrow “peripartum onset” specifier for depressive disorders in the Diagnostic and Statistical Manual of Mental Disorders 5th Edition Text Revision (DSM-5-TR) that is limited to symptom onset in pregnancy and within the first 4 weeks postpartum.^15,16^

Anxiety is even more common than depression in the perinatal period, with rates of clinically significant anxiety symptoms estimated at up to 20%.^2,17^ The prevalence of the related clinical presentation of fear of childbirth may be as high as 14%.^18^ Pooled prevalence of OCD is about 2% perinatally, although transient obsessions and/or compulsions may be more common, and there have been reports of transient symptom worsening perinatally possibly in as many as 70% of those affected by the illness.^19–21^ PTSD has an estimated pooled prevalence of about 3% in pregnancy and about 4% after birth.^22^

The lifetime prevalence of BD is about 2–3% in non-pregnant populations, and diagnostic interviews in pregnancy and postpartum suggest the same.^23^ BD has the highest relapse rate for any mental disorder in the postpartum, in the range of 30–50%; the relapse rate may be as high as 66% among those who are medication-free during pregnancy.^24^ Most relapses are non-psychotic MDEs but as many as 1 in 6 (17%) are episodes of psychosis, mania or MDEs severe enough to require hospitalization. Postpartum psychosis occurs after approximately 1–2 in every 1000 births overall,^25^ but risk is increased in those with a pre-existing diagnosis of BD, and those who have previously experienced postpartum psychosis.^26,27^

While a comprehensive description of risk factors for each specific PMAD is outside the scope of this guideline, Table 4 lists common risk factors and correlates.^3,28–38^ The strongest risk factor for any PMAD is a past history of the given disorder, and untreated or undertreated symptoms prior to or during pregnancy.^39^ Lack of social support and stressful life events are major risk factors for perinatal depression and anxiety.^3^ Perinatal depression appears to be more commonly experienced by some populations, including adolescents, immigrants,^40^ those who live in lower and middle-income countries,^41,42^ are racialized,^43^ Indigenous,^44,45^ have disabilities^46,47^ and are gender and sexual minorities.^48^ Anxiety disorders also appear to be more common in low and middle-income countries^41^and underserved populations.^17,41,49^ PTSD is more common in those who have experienced pregnancy complications, stillbirth and adverse childhood experiences.^22^ Nulliparity and lower socioeconomic status are associated with increased risk for fear of childbirth.^50,51^

**Table 4.** Common PMAD Risk Factors.

Genetic, mental and physical health factors
Personal history of PMADs, mental illness related to other reproductive life stages (e.g. premenstrual syndrome or premenstrual dysphoric disorder) or significant psychiatric symptoms with oral contraceptives Personal history of non-perinatal mood, anxiety and related disorders and/or comorbid mental illnesses, including eating disorders, and alcohol and substance use disorders Family history of mental illness, especially perinatal mental illness and especially in first-degree relatives (i.e., mother, sister) Chronic medical conditions
Reproductive, perinatal and postpartum health factors
Unintended pregnancy Primiparity Multiple births (e.g., twin, triplet) Pregnancy complications (e.g., hyperemesis gravidarum, gestational diabetes mellitus, gestational hypertensive disorders, such as preeclampsia) Birth and neonatal complications (e.g., preterm birth, stillbirth, low birth weight) History of pregnancy loss Sleep disturbance in pregnancy, during and after delivery, and postpartum Difficulty with breastfeeding (or early cessation of breastfeeding)
Social and environmental factors
Age (adolescents and childbearing people >40 years) Low socio-economic status Stressful life events (e.g., birth complications, experience of trauma around the time of the birth, illness in a child, death of a loved one, unemployment/financial strain, divorce) Low social support, including emotional and instrumental supports Low partner support/relationship discord Domestic/intimate partner violence History of sexual or physical violence

*Note*. PMAD = perinatal mood, anxiety and related disorder.

The effective treatment of PMADs is of high priority for the short- and long-term health of the affected person, their child and family. Untreated or inadequately treated perinatal depression is linked to an increased risk of future depressive episodes, substance use, partner relationship problems and suicide.^52–55^ Depression in pregnancy is associated with perinatal and newborn health complications (e.g., gestational hypertension,^56^ preterm birth,^57,58^ low birth weight^58^ and neonatal intensive care unit (NICU) admission in infants^59^). Depression in both pregnancy and postpartum are associated with lower rates of breastfeeding,^60^ less optimal mother-infant interactions^61^ and higher rates of infant and child physical illness,^62^ hospitalizations,^63^ maltreatment^64^ and cognitive, emotional and behavioural problems.^65,66^ The effects of untreated or undertreated anxiety and related disorders on the health and well-being of childbearing individuals and their offspring are similar in nature and magnitude to those of depression.^3,66–68^ Fear of childbirth specifically is associated with higher rates of labour interventions and complications, such as emergency caesarean birth.^50,51,69,70^ BD is also associated with increased risks for adverse perinatal and newborn health complications and an increased risk for PPD and psychosis.^71,72^

## Foundations of Clinical Management

### Initial Diagnostic Assessment

Regardless of the manner in which a potential PMAD is identified (see Question 1), the first step in clinical management is a comprehensive diagnostic assessment. It is important that this assessment is conducted in a supportive, non-judgmental and inclusive environment. Accessible, culturally safe and trauma-informed care are all essential to a comprehensive assessment and management plan. Clinicians are encouraged to educate themselves about how to safely care for diverse populations, including those who are racialized, a gender or sexual minority, or who have a disability and others. It is important for providers to consider the systemic ways in which some populations have been historically underserved, mistreated, excluded and/or stigmatized (including in health care encounters and systems) and how the experiences, outcomes and needs of certain populations may differ as a result.

The initial diagnostic assessment includes all elements of a general psychiatric interview, including the patient's goals and expectations with the assessment, their sociodemographic characteristics, current and lifetime mental health and substance use disorders and their severity (including hospitalizations and suicidal behaviours), general medical history, current and past treatment response to psychological, pharmacological and other interventions (as well as what happened when prior interventions were discontinued), and developmental and family history. It is important to evaluate past and current risk factors for PMADs, family and social support, the roles and responsibilities of the patient with respect to caregiving (including for other children), and financial or work obligations. It is also important to inquire about health-related factors that can influence mental health and may be targeted through lifestyle interventions, such as exercise routine, diet and sleep patterns. Clinicians will want to assess for potential impact of the co-parent's mental health (see Question 10) and/or whether there are concerns about intimate partner violence. Mental health issues in relation to previous pregnancies and other times of hormonal change (premenstrual disorders, mood worsening with the use of hormonal contraceptives, for example) are associated with elevated risk for subsequent PMADs.^36^ History of pregnancy, labour and delivery and/or postpartum complications, including pregnancy loss, can also inform the diagnostic and management plan.

*Clinical Pearl—Trauma-Informed care*

Questions about past trauma are important to explore with sensitivity.^73,74^ Traumatic experiences can have long-lasting, pervasive impact on one's mental and physical health, but many individuals do not spontaneously disclose their past traumatic experiences due to fear, shame, feelings of blame and/or lack of trust. It is not uncommon for patients who have previously experienced trauma to present as guarded, sensitive and prone to emotional dysregulation. In the perinatal period, triggers of prior traumatic experiences can specifically include physical examinations, labour pain, difficult childbirth or other clinical scenarios where the patient does not experience adequate control, choice or autonomy. Trauma-informed care—different than intervention focused on treatment of the sequelae of trauma itself—refers to when healthcare clinicians and care systems are able to acknowledge the broad impacts of trauma, recognize clinical symptoms or other evidence of trauma in the clinical setting and integrate this knowledge into how care is delivered including by creating a safe environment, promoting collaborative treatment relationships and decisions and taking practical steps to reduce the likelihood that the treatment environment will mirror common characteristics of past traumatic experiences.^73,74^ Given the high prevalence of trauma, trauma-informed care is important at every stage of the assessment and treatment process for all persons with PMADs.

It is important to explore how and when a patient's symptoms may have started or changed in pregnancy and/or in the postpartum, to identify potentially addressable factors that may have led to the development or worsening of illness. This may help identify whether there has been an increase in stressful life events, whether medication discontinuation may have precipitated relapse or whether there is a need for medication adjustment due to the physiological changes of pregnancy and/or the postpartum period. In the postpartum, exploring infant/newborn health can similarly inform diagnosis and treatment (i.e., preterm, difficult labour/delivery, medication withdrawal, feeding, sleeping, irritability, colic and sibling relationships).

The initial diagnostic assessment should also include a safety risk assessment, including thoughts of self-harm or harm to the infant/others (see Question 9). When possible and appropriate to obtain, collateral information from family members, friends and/or acquaintances can be helpful in clarifying diagnosis and risk. Thyroid-stimulating hormone (TSH) and complete blood count (CBC) or ferritin measurement can assist in ruling out common conditions that occur in pregnancy and postpartum such as thyroid disease or iron deficiency/anaemia, respectively. Other laboratory tests may be required on a case-by-case basis (e.g., Vitamin D levels with suspicion of Vitamin D deficiency). Additional testing and/or the use of electroencephalography (EEG) or neuroimaging are generally only indicated when there is clinical suspicion of epilepsy/seizures, traumatic brain injury or other serious medical conditions that can cause neuropsychiatric symptoms (e.g., anti-N-methyl-D-aspartate, anti-NMDA, receptor encephalitis or posterior reversible encephalopathy syndrome).

### Diagnostic Considerations

Not all mood and anxiety symptoms experienced perinatally necessarily represent a psychiatric disorder. The “baby blues,” reported by 40% to 80% of individuals shortly after childbirth, includes symptoms such as mild mood swings, sadness, crying more easily, anxiety, and difficulties with sleep and concentration.^75^ Symptoms associated with the “baby blues” are neither persistent nor severe and usually improve significantly or disappear within a few weeks without intervention. Although less well-studied than the “baby blues,” the concept of “baby pinks” which appears to include mild symptoms of mood lability, irritability, feeling euphoric/elated, sleeplessness, increased energy and over-talkativeness, has also been reported.^76^ Like the “baby blues,” symptoms of the “baby pinks” also appear to start within the first 2 weeks postpartum, are mild, do not negatively impact daily functioning and improve without treatment. Longitudinal studies show that most cases of “baby blues” and “baby pinks” spontaneously remit and do not require medical intervention. However, some studies suggest a higher risk of PPD at 6–8 weeks postpartum in individuals who experience them.^75,76^ Therefore, CANMAT recommends monitoring those who experience “baby blues/pinks” carefully until resolution of symptoms (Level 4 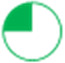). In cases of pregnancy or child health complications, unexpected occurrences during labour and delivery that are experienced as traumatic, difficulty with breastfeeding or other major stressors, a DSM-5-TR diagnosis of adjustment disorder may be more appropriate than one of a PMAD, if criteria for another mood, anxiety or related disorder are not met.

In the DSM-5-TR, PMADs do not have their own diagnostic criteria, chapter or category.^16^ There is a specifier of “peripartum onset” for MDD, BD I and BD II that is used when the onset of these disorders occurs in pregnancy or within the first 4 weeks postpartum. In practice, those with onset of mental illness prior to pregnancy will also require treatment perinatally. Further, as mental illness can also arise after the first 4 weeks postpartum, both clinicians and researchers usually consider disorders to be “perinatal” when they present in individuals up to at least 1 year postpartum.^77^ Despite their high prevalence, there are no peripartum onset specifier for anxiety disorders, OCD or PTSD at this time.

Although there are no specific DSM-5-TR symptom criteria for perinatal populations, there are some perinatal-specific aspects of the clinical presentations. In depression, highly negative and persistent ruminations about one's capacity as a parent and guilt about actions having a negative impact on the child are common. Anxiety—whether as part of a depressive, anxiety or related disorder—often focuses on worries related to the health of babies and the ability to parent.^78^ The intrusive nature of excessive worrying perinatally in depression and anxiety disorders may sometimes be difficult to distinguish from that of OCD (“obsessive worrying”), which is characterized by intrusive thoughts and/or compulsive behaviour. In any of these conditions, intrusive worries in the perinatal population can present as unwanted, intrusive thoughts or images of harm coming to the infant, often accompanied by reassurance seeking, checking behaviour and avoidance (e.g., avoiding letting others look after the baby).^19^ Fear of childbirth (also referred to as tokophobia) is an increasingly recognized clinical presentation of anxiety that encompasses many different fears related to pregnancy and childbirth, ranging from fear of pain, medical interventions and injury, to fear of loss of control, body change and staff misconduct, to fear of maternal and/or infant death.^18,51^ CB-PTSD, where PTSD symptoms focus on the birth and reminders of the birth in the context of a traumatic birthing experience, is now increasingly being described.^79^ It is also increasingly being recognized that childbirth can be experienced as traumatic and lead to poor mental well-being even when there were no serious obstetrical or neonatal complications per se.

Although this guideline is specifically focused on the management of PMADs, it is critical for clinicians to consider that mood and anxiety symptoms may be associated with other psychiatric conditions, including personality disorders (e.g., borderline personality disorder), eating disorders (e.g., anorexia, bulimia, binge eating) and/or alcohol and substance use disorders. In fact, in some cases, these psychiatric disorders other than PMADs may be the primary source of mood and anxiety symptoms. Whether psychiatric disorders other than PMADs are primary or comorbid with PMADs, they need to be fully assessed and managed. Finally, in Canada, clinicians have the duty to report to child protection services or the police (when urgent) in situations where there are concerns about child abuse and/or neglect. CANMAT recommends all clinicians to be aware of the laws of their local authorities.

### General Treatment Considerations

It is ideal for mental illness to be stabilized prior to conception. In preconception planning, it is important to provide patients with updated information to help them balance the potential risks of their illness (or illness recurrence) with various treatment options when making decisions about plans for their treatment before, during and after pregnancy. In pregnancy and postpartum, the main goals of treatment are to achieve symptom remission, prevent recurrence or worsening, reduce or eliminate symptoms of comorbid disorders, including medical, psychiatric and substance or alcohol use disorders, and minimize any risks to the developing infant or child. Healthcare providers are encouraged to provide support for the management of psychosocial issues, which may include connecting patients to social services early in treatment planning. This may include connecting the patient with resources to assist with food insecurity, financial and/or housing instability, language classes for those who do not speak the local language, and/or supporting patients experiencing intimate partner violence. For individuals presenting with more than one psychiatric disorder, it is important to prioritize which disorder(s) need(s) to be addressed first. Factors to consider include the type and severity of disorders, symptom profile (e.g., presence of psychotic symptoms), impact on maternal distress and functioning, and safety concerns. Collaboration between multidisciplinary team members is encouraged in preparation for labour, delivery and the early postpartum period, especially in complex cases.

**Figure 1.**
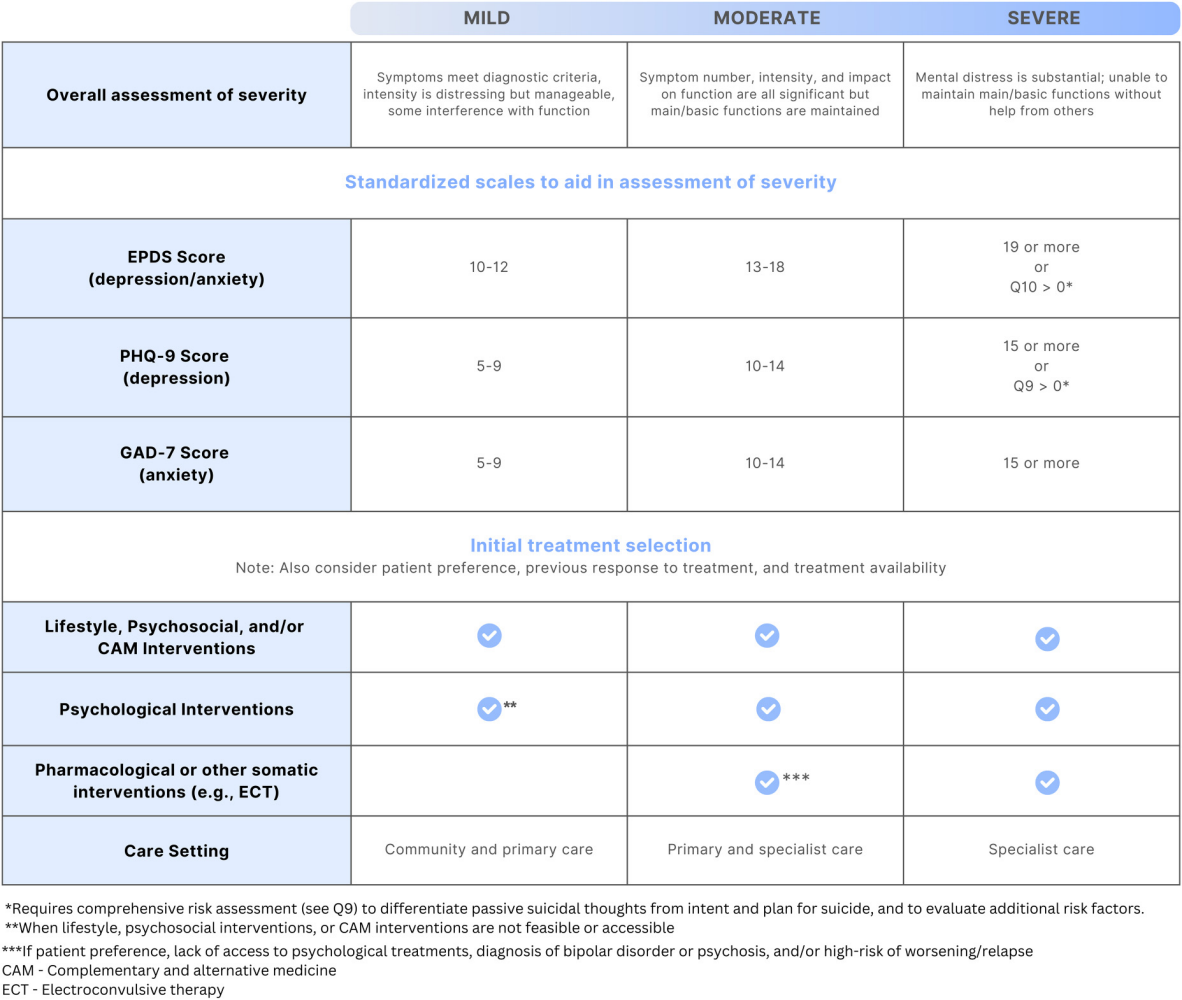
How PMAD illness severity can guide initial treatment selection. For a given illness severity, the lowest checkmarked intervention type is typically needed, but interventions in higher rows may also be implemented to complement the effect of the main intervention.

Initial treatment selection depends on the nature and severity of the illness, previous response to treatment and patient preference. Severity (mild, moderate or severe) can be assessed based on the number of symptoms that a patient is presenting with, the severity of those symptoms and the social and occupational functional impact (Figure 1).^16^ For depression and anxiety symptom severity specifically, scores on brief patient-oriented outcome measures commonly and feasibly utilized in clinical practice such as the Edinburgh Postnatal Depression Scale (EPDS), Patient Health Questionnaire-9 (PHQ-9) and Generalized Anxiety Disorder 7-item (GAD-7) may be used to aid in the severity assessment.^80^ The cut-off scores on these measures for the various severity levels in Figure 1 are meant only to be used to guide the severity assessment, and should be considered in the context of other aspects of the clinical picture (e.g., history, duration of symptoms, other comorbidities) to aid in decision-making about treatment.

PMAD = perinatal mood, anxiety and related disorder.

Source: Adapted from the Care Pathway for the Management of Perinatal Mental Health (Ontario Provincial Council for Maternal and Child Health).

As shown in Figure 1, depression, anxiety and related disorders of mild or moderate severity can often be successfully managed with non-pharmacological options alone, including with lifestyle, psychosocial, psychological and complementary and alternative medicine (CAM) interventions. Medications and other somatic treatments may then be used in addition when symptoms do not respond to non-pharmacological treatment alone. As also shown in Figure 1, medications are typically needed to successfully manage depression, anxiety and related disorders when symptoms are of moderate-severity or severe, and in the treatment of BD. They are also used to prevent relapse or recurrence of symptoms when an individual was taking medication for maintenance treatment prior to pregnancy. In these cases, non-pharmacological interventions may also be implemented to complement the pharmacological treatment (e.g., peer support programs or psychological therapy may still be beneficial alongside a medication treatment). Other somatic treatments, such as non-invasive neuromodulation or electroconvulsive therapy (ECT) may be needed when medication options have not led to adequate remission, or for ECT when there is very severe illness and a rapid response to treatment is required (e.g., acute psychosis or catatonia).

When discussing treatment options, healthcare providers should provide ample time for discussion, including possible risks, side effects and expected benefits. Patient preference should also be taken into account. Health professionals should also encourage patients to discuss the treatment options with their partners/close support and/or to bring their partners/close support to an appointment so that treatment options can be discussed jointly, as appropriate.

Once treatment is initiated, CANMAT recommends regular monitoring. The routine use of standardized tools measuring the severity of symptoms such as the EPDS, PHQ-9 and the GAD-7 is highly encouraged to systematically monitor response to treatment and potential symptom worsening or relapse. Monitoring should occur more frequently while the patient remains symptomatic (e.g., every 1–3 weeks) and the frequency can be reduced once the symptoms have fully remitted (e.g., every 6–8 weeks) (Level 4 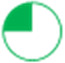).

## Question 1. What Are the Recommendations for Identification of PMADs?

The first step in the pathway to effective diagnosis and treatment is case identification. Some women and pregnant or birthing people of other genders may recognize and report symptoms themselves, while others will not. Friends or family may identify symptoms and express concerns. Clinicians may also find themselves uncertain about the level of symptomatology that suggests a diagnosable disorder to be present. As such, while case identification can be made through sensitive clinical inquiry about mental health, the use of standardized clinical questionnaires to help identify cases has been investigated.^81^

The EPDS is a well-validated tool for the detection of perinatal depression that has been translated into 60 languages.^82,83^ While multiple cut-off scores have been suggested in the literature, evidence from a large systematic review suggests that a cut-off score of 11 or higher on the EPDS maximizes the ability to accurately identify depression, when compared to semi-structured diagnostic interviews in pregnant and postpartum populations (Level 3 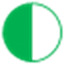).^84,85^ While we recommend the EPDS preferentially, the PHQ-9, one of the most widely used depression self-report tools in primary care,^86^ has comparable test characteristics at a score of ≥10, so can be used if more practical to implement (Level 3 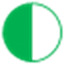).^87^ The PHQ-2 and Whooley Questionnaire have more moderate specificity (Level 3 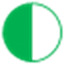),^88^ but may be useful when the longer questionnaires are not feasible despite the trade-off of generating more false-positive cases.

Data are still emerging on the validity of standardized tools to identify cases of anxiety and related disorders in perinatal populations.^89–93^ For generalized anxiety disorder (GAD), the GAD-7 scale (widely used in GAD screening non-perinatally), there is one study in pregnancy that suggests that a cut-off score of 7 may have better sensitivity and internal consistency than the score of 10 that is usually used for clinically meaningful symptomatology outside of the perinatal period.^94^ There is limited data about optimal scales and cut-off scores for the detection of other anxiety disorders, OCD, or PTSD in the perinatal period, although studies in this area are also emerging.^95,96^ Given the importance of case identification, if there is clinical suspicion, scales validated outside of the perinatal period—or those with some validation within the perinatal period—can be used with caution (Level 4 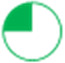).^92^

When a patient presents with symptoms of depression, anxiety or related disorders, it is also important to determine whether there is a current or past history of mania or hypomania because that may significantly alter the proposed treatment plan. The Mood Disorder Questionnaire (MDQ), a widely used instrument in non-perinatal populations, has evidence in the identification of BD in perinatal populations when a cut-off score of 7 or above is used and the supplementary questions are excluded (Level 3 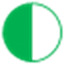).^97–100^ The MDQ is excellent in ruling out the diagnosis of BD (>90% negative predictive value). However, positive cases need to be followed by a diagnostic assessment since only 50% of those scoring positive in the MDQ have a diagnosis of BD. The Highs, although less studied than the MDQ in non-perinatal populations, is the only instrument developed specifically for the detection of acute hypomania and mania symptoms in the postpartum period. The Highs is a 7-item questionnaire, where a score of 8 or higher can be used to detect BD, particularly when used at 1 and 6 weeks postpartum.^101^ The MDQ would be best utilized for assessment of past and acute symptoms whereas the Highs is best utilized to measure the severity of acute symptoms.

When using a standardized questionnaire for case identification, typically a cut-off score above a certain number represents a high likelihood of diagnosis. However, diagnoses cannot be made through questionnaires alone. When there is a concern for PMADs due to clinical suspicion and/or score on a questionnaire, the patient should receive further diagnostic assessment and treatment. It is also possible for an individual with a score below the threshold to still require care. As such, it is critical that when standardized clinical scales are used for case detection, that there is also a mechanism or pathway for proper diagnostic assessment and treatment.

One area of discussion has been whether standardized clinical questionnaires to identify PMADs should be used routinely with all perinatal patients. For perinatal depression, there is evidence that the use of a standardized questionnaire for case identification can improve identification rates, treatment rates and clinical outcomes (Level 2 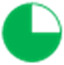).^102,103^ Given the high prevalence of perinatal depression, the fact that perinatal people may not disclose their mental health concerns when not asked about them, the well-established risks of untreated illness and the validity of various standardized scales in identifying perinatal depression, the US Preventive Services Task Force has positioned themselves in favour of this strategy, as have organizations from other countries.^102,104^ In contrast, the Canadian Task Force on Preventive Health Care recommended against the routine use of standardized questionnaires with concern that the evidence in favour of the strategy was not strong enough to outweigh the potential economic costs and stress to patients of false-positive cases.^105^ Although there is high acceptability of the use of standardized questionnaires for case identification in perinatal populations,^93,106^ there was concern that use of a cut-off score on a standardized questionnaire on its own could lead to misdiagnosis or overdiagnosis, increased mental health stigma and unnecessary treatment.^107^

CANMAT believes that it is essential to ensure that cases of depression are identified as quickly and early as possible to promote timely and effective treatment, such that the evidence to date –which includes the good validity and acceptability of questionnaires in case identification and the Level 2 evidence for improvement of clinical outcomes—is sufficient to support the use of validated questionnaires to help in case identification. While standardized questionnaires may be helpful in identifying cases of PMADs other than depression as indicated previously, there is not yet evidence for how applying such tools routinely would impact on clinical outcomes. Further research on the feasibility and effectiveness of systematic or routine use of questionnaires to detect other PMADs is needed.

While there is no empirical evidence on the optimal timing for case identification and the feasibility of integrating questionnaires into routine care may be challenging in some settings, CANMAT recommends that clinicians providing antenatal, postnatal and/or pediatric care implement case identification of PMADs into their clinical practice throughout the perinatal period (e.g., once per trimester pregnancy, at postpartum obstetrical follow-up, in family practice and pediatric care up to 12 months postpartum) (Level 4 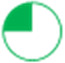).^102,108,109^

### Considerations in Diverse Populations

While some well-validated tools have been translated into many languages, some populations may not recognize or experience mental health symptoms in a manner that is accurately captured by case identification tools standardized in a different population.^110,111^ Further, it has been found that local language versions of tools like the EPDS may been less likely to identify true cases of PMADs compared to the original English version.^112^ It may be beneficial to use tailored cut-off scores on questionnaires in specific populations, as evidenced in a recent scoping review showing that lower cut-off scores may be more appropriate in South and/or East Asian populations.^113^ There is limited evidence for optimal scales and cut-off scores in adoptive parents,^82^ and for those who are Indigenous or racialized.^113–115^

Some racial and ethnic minority and migrant populations may be less likely to disclose mental health concerns due to the stigma surrounding mental illness and expectations of motherhood within their family or culture.^110,116–119^ There may also be a reluctance to disclose mental health challenges perinatally among those with disabilities due to fears of mistreatment or child apprehension.^120^ For people with intellectual and developmental disabilities, tools like the EPDS may not be as useful given this population's challenges interpreting and recalling symptoms.^121^ There is also evidence that populations who are marginalized by racism and socioeconomic disadvantage are less likely to be assessed for PMADs.^108^ Further efforts to create culturally appropriate and accessible case identification tools and to examine and address inequities in assessment are needed.

## Question 2. What Is Recommended for the Organization and Delivery of Healthcare Services?

### Barriers to Care

Initial identification of PMADs may occur in antenatal or postpartum care, including in midwifery, public health nursing, primary care (e.g., family physicians, nurse practitioners), pediatrics or obstetrical settings. Ideally, healthcare services will be organized such that once PMADs are identified, there are clear pathways to assessment and treatment. Therefore, it is important to consider barriers to care and how to address them at the individual, healthcare provider and health system level, to improve treatment access and outcomes.

Multiple barriers to care for PMADs have been described.^122^ Key barriers include stigma, feelings of shame, guilt, embarrassment and lack of self-efficacy (i.e., a lack of confidence that there is anything that is under a person's control that could help them to feel better). Sometimes patients and clinicians may lack knowledge about the symptoms of PMADs and that there are effective treatment options, or have specific perceptions about treatment that lead them not to want to seek care (e.g., concern that a medication may be the only option recommended when they prefer psychotherapy). Many patients have reported feeling too overwhelmed to seek care and some report fears of disclosing their mental health symptoms due to concerns about losing parental rights to their children. Further, interventions are effective only if they are accessible. While some can be implemented within one's home, or with little to no cost, many interventions require funding, qualified personnel and other resources (e.g., transportation, childcare). Practical barriers to care include inadequate health insurance or financial issues, lack of childcare, time, transportation and/or internet connections for virtual services, as well as problems with care availability (i.e., there is variable access to specialized services in most jurisdictions with fewer treatment options especially in rural and remote regions).

Some populations, including those who are adolescents, disabled, gender-diverse, immigrants and refugees, Indigenous, racialized and sexual minorities, disproportionally experience barriers to care.^48,123–126^ Specifically, socioeconomic disadvantage and the structural impacts of colonization, racism, homo/bi/transphobia, ableism, and migration and country, community integration or settling experiences, impact access to care, provider-patient interactions, and in turn can negatively impact perinatal mental health outcomes.^127^ These structural factors and their legacies, including (intergenerational) trauma, must be considered and integrated into the training of care providers to ensure that all childbearing people receive safe, accessible and equitable perinatal mental health care.^128^

There is evidence that some racial and ethnic minority populations have lower rates of treatment initiation and engagement and higher dropout rates of psychological interventions despite very strong preferences for psychological over pharmacological interventions.^116,129^ Some barriers to care discussed above may partly explain race- and other identity-based differences in treatment preference modality, initiation and adherence. Culturally sensitive care and cultural adaptations of psychosocial and psychological interventions can reduce inequities in treatment adherence, as well as improve clinical outcomes among racial and ethnic minority groups.^117,130^ Clinicians are advised to provide all the information necessary for patients to make an informed decision, preferably in ways that are most accessible or applicable to that person (e.g., recommendations around web-based or social media resources, providing visual and printed materials, in various languages, etc., as opposed to use of medical or technical language). Clinicians are also advised to consider patients’ specific life circumstances, notably barriers to and/or availability of financial, social and other resources to support specific intervention initiation and adherence.

#### Models of Care

Strategies that have been suggested to decrease barriers and facilitate the implementation of perinatal mental health policy and practice are summarized into:^119^ (a) Design of care: Patients should have a choice in the care they receive; easy access; care should be delivered clearly, openly and honestly; flexible times of the appointments; practical supports such as assistance with childcare, transport and links with social work; access to interpreters if needed; and options for both in-person and virtual care; (b) Characteristics of healthcare providers: Healthcare providers should be open, non-judgmental, willing to listen, motivated, sensitive to verbal cues, interested and well-trained in perinatal mental health issues, dedicated to act as patients’ advocates, culturally sensitive, knowledgeable and confident, good communication skills and feel positively about the care they provide; (c) Organizational factors: There should be clear workflow procedures; collaborative working; personnel dedicated to perinatal mental health; and supervision; (d) Political factors: There should be free care or easy access through insurance policies (in Canada, many psychological services are not covered by universal healthcare insurance; in other settings there can be variability in access to many different types of services based on insurance status); services adequately funded with proper services and staff; clear and easy pathways of referral; and (e) Societal factors: There should be patient, family and public education to improve health literacy and support, and decrease stigma.

Unfortunately, most healthcare jurisdictions have not implemented systematic management pathways for PMADs. Instead, health care providers such as family physicians, midwives, nurses, obstetricians and pediatricians who identify possible cases of PMADs often rely on ad hoc referrals to local therapists or general mental health services, with referrals to more specialized perinatal mental health services only in regions where these are available. Similar to issues with mental healthcare in non-perinatal populations, it is thought that the lack of a systematic approach to perinatal mental health care delivery contributes to low treatment rates. Because of this, various models of care have been developed and evaluated in an effort to better organize and deliver care (Table 5).

**Table 5.** Perinatal Mental Health Care Delivery Models.

Line	Model of care	Level of evidence
First-line	Collaborative care	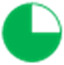
Second-line	Stepped care	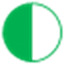
Third-line	Staged care	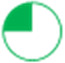

Collaborative care, also known as integrated care, is a mental healthcare model that uses interdisciplinary contributions by 2 or more health professionals to systematize case identification, assessment, triage and referral, initiation of treatment appropriate to the level of patient symptoms and risk, and ongoing/continuous follow-up symptom monitoring and management for entire patient populations.^131^ These models are typically based in primary or obstetrical care settings, with a mental-health trained behavioural health coordinator (e.g., RN) to assess, triage and monitor care and psychiatrists to advise primary care clinicians (i.e., family physicians and nurse practitioners) or obstetricians and oversee treatment programs. In this model, patients with mild and even moderate severity of symptoms can have their concerns initially addressed via front-line providers. For example, patients can be directly supported with social services, connections to peer support and other interventions in the primary care or obstetrical setting. Then, psychiatric support can be available when these initial interventions are insufficient or in more severe cases. Integrating mental healthcare into general healthcare settings (e.g., primary care and obstetrical or midwifery care) achieves greater access to treatment and continuity of care, which can provide benefits for patient mental health and obstetric outcomes.^132^ Collaborative care models are associated with high treatment satisfaction, and have been shown to be effective for the treatment of depressive and anxiety disorders in perinatal settings in some although not all trials (Level 2 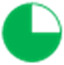).^133–135^ There is emerging evidence for perinatal psychiatric access programs, an approach that incorporates elements of collaborative care, but with less resource intensity, where health care providers can reach out to a specialized centre for resource and referrals, telephone provider consultation and direct patient consultation with expert perinatal psychiatrists.^136^

Stepped care models involve providing patients with treatments of increasing intensity depending on the severity of illness.^137^ Patients are typically advised to begin with the least intensive treatment shown to be effective for their symptom severity and then to ‘step up’ into more intensive interventions depending on their treatment response. These models are currently in use to deliver perinatal mental healthcare in certain national health systems (e.g., in the UK), but have not been extensively studied for PMADs (Level 3 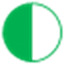).^138^ In staged care models, patients are matched through a range of methods, including self-report questionnaires, to treatments according to their symptom severity.^139^ Staged care models have not been rigorously tested in perinatal populations. Some studies describe program development and there are non-randomized studies focusing on PPD and comorbid anxiety that show some promise for this type of intervention (Level 4 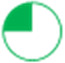).^140–143^

Stepped and staged care models differ from collaborative care models in that they do not necessarily encompass the population-based assessment and monitoring components. However, in health care systems where more comprehensive integrated care models have yet to be implemented, these approaches may be helpful. For example, in some Canadian jurisdictions, clinical care pathways have been developed to support antenatal, postnatal and pediatric care providers in assessing the severity of a patient's PMAD symptoms and directing the patient to a specific step in the pathway based on this (e.g., peer support for mild symptoms, psychological therapy for symptoms of moderate-severity or medication for more severe symptoms).^80,144^

Studies of mental health care models for perinatal mental health have largely focused on interventions for depressive and anxiety disorders. Further research is needed beyond perinatal depression and anxiety, including in BD, PTSD, OCD and other perinatal mental disorders.^145,146^ Further research is also needed to determine the effectiveness of mental health care models in under-served populations, such as disabled, sexual minority, Black and Indigenous populations.^147,148^

#### Treatment Providers and Settings

Psychosocial interventions may be delivered in the community by peers, nurses and other community healthcare providers. Psychological interventions are usually delivered by trained mental health care professionals. However, task-sharing—the “rational distribution of tasks to less specialized personnel”^149^—can be leveraged to improve access to screening, self-care options and frontline psychological treatments.^150,151^ For example, brief psychological treatments to prevent and treat PMADs can be delivered by non (mental health) specialist providers.^152–154^ Medications are usually prescribed by primary care clinicians (i.e., family physicians, nurse practitioners) and sometimes obstetricians. Psychiatrist input and follow-up is most important in complex or more severe cases, such as when there are comorbid conditions, inadequate response to initial treatments and in high-risk situations. Virtual delivery of care such as by telephone or video-conferencing can improve access to care by eliminating barriers to care, such as arranging childcare, transportation and geographical barriers, among others, with comparable effectiveness to in-person care for perinatal depression and anxiety (Level 2 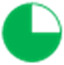).^155–165^

For individuals with more severe illness, intensive psychiatric day programs (also called “day hospitals”) have been developed, although these have only been evaluated in non-randomized studies (Level 4 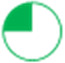).^166,167^ For severe postpartum mental illness, mother–baby units (MBUs, not currently available in Canada) have been proposed as a means of providing specialized mental health treatment options for postpartum people with severe forms of mental illness without mother–infant separations. MBUs are meant to support the mother–infant relationship, while stabilizing maternal mental health. Evidence from non-randomized, uncontrolled studies shows positive effects on maternal mental health, mother–infant relationships and child development (Level 4 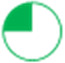).^168,169^ As there would be significant practical challenges to conducting randomized trials to provide additional evidence comparing MBUs to the standard of care, it may be important for jurisdictions currently without MBUs to consider that they may be particularly helpful in certain situations, especially for patients who may require prolonged hospitalizations (and thus long separations from their infants).

The range of different providers and settings in which perinatal mental health care can be accessed in Canada and other countries can result in a treatment landscape that is difficult to navigate.^170^ Patient navigation aims to facilitate timely access to mental healthcare by helping patients understand what services may be helpful for them, and connect them to the right service option (e.g., by identifying service options, coordinating with services to assist with referrals, and helping to remove access barriers).^171^ It has shown some promise in terms of clinical outcomes (e.g., reduced symptoms of depression and anxiety), patient satisfaction and service use (Level 4 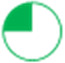).^171^ Patient navigation's effectiveness on its own without the actual connection to services that would occur in a collaborative, stepped or staged care model is not known.

## Question 3. What Are the Recommendations for Lifestyle Interventions?

CANMAT considers lifestyle interventions as those that serve to intervene on health-related behaviours, including as this relates to diet, exercise and/or sleep. Prior CANMAT guidelines have also included light therapy in this category of interventions. A summary of lifestyle interventions for the prevention and treatment of PMADs is in Table 6.

**Table 6.** Lifestyle Interventions for the Prevention and Treatment of PMADs.

Line of treatment	Depressive symptoms		Anxiety symptoms	
	Intervention	Level of evidence	Intervention	Level of evidence
**Prevention**				
**First-line**	Low to moderate-intensity exercise	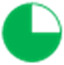	—	
**Second-line**	—		Low to moderate-intensity exercise	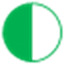
**Third-line**	^a^Sleep protection especially in at-risk groups	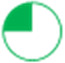	—	
**Not recommended**	Sleep education in non-clinical populations does not appear to prevent PPD	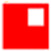	—	
	Infant behavioural sleep interventions in non-clinical populations may help with child sleep and maternal sleep quality but do not appear to prevent PPD	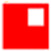		
**Treatment**				
**First-line**	Low to moderate-intensity exercise	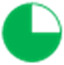	—	
	Bright light therapy (postpartum)	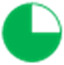		
**Second-line**	—		Low to moderate-intensity exercise	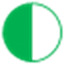
**Third-line**	^a^Sleep protection	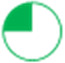	^a^Sleep protection	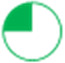
Sleep protection interventions are highly recommended for the prevention of relapse in perinatal bipolar disorder, including to help prevent postpartum psychosis and are also essential in the treatment of these conditions (Level 4 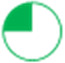 ) (see Question 9 for additional details)				

*Note*. Exercise should be conducted under supervision to ensure clinical appropriateness. At-risk groups = those with a past history of, or current evidence of PMADs, and especially bipolar disorder and/or postpartum psychosis; CBT = cognitive–behavioural therapy; PMAD = perinatal mood, anxiety and related disorders; PPD = postpartum depression.

^a^ Sleep protection interventions may range from minimizing the time a mother has to be awake at night (e.g., bottle-feeding by another adult with pumped breastmilk or formula) to evidence-based interventions to treat insomnia when it is present. CBT for insomnia, CBT-I, is a highly effective treatment for insomnia where there is specifically effectiveness demonstrated for improving sleep quality, insomnia severity, depression and anxiety symptoms in pregnancy (Level 1 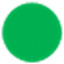), and open-label evidence for its use for insomnia in postpartum depression specifically (Level 4 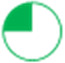).

The evidence supports exercise interventions as helpful in preventing depressive symptoms from developing in pregnancy and postpartum in non-clinical populations (Level 2 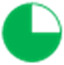).^172,173^ Exercise of at least moderate intensity and greater than 150 min per week appears to most reduce the risk (Level 2 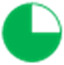).^173^ In terms of treatment, aerobic exercise has been associated with small to medium reductions in depression symptom severity, although available RCTs are of low-moderate quality (Level 2 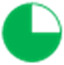).^174–178^ This applies to low-intensity activity (including walking), although moderate-intensity activity may lead to greater symptom reduction.^175,176,179^ Exercise interventions have also been shown to prevent anxiety symptoms and reduce their severity in pregnancy (Level 3 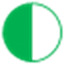).^180^ The extent to which these findings related to perinatal depressive and anxiety symptoms would generalize to those who meet diagnostic criteria for depressive, anxiety, anxiety-related or BDs is unknown. Regardless, exercise recommendations should be tailored to individual circumstances (e.g., finances, childcare support, physical limitations, other barriers), preferences, values and possible health risks (e.g., eating disorders, injuries) and adapted for the context of a person's pregnancy and post-delivery recovery circumstances.

Sleep disruption is common in pregnancy (e.g., due to general discomfort, need for frequent urination and/or medical conditions such as sleep apnea) and when caring for an infant, and is linked to increased risk for and worsening symptoms of PMADs.^181–184^ Neither universal sleep education interventions nor behavioural sleep interventions for the infant (e.g., settling methods, such as “cry it out” or “fading,” or bedtime routines) appear to effectively prevent PPD in non-clinical populations (Level 2, 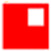 negative).^185,186^ However, behavioural sleep interventions can help with child sleep and maternal sleep quality. Also, sleep disruption increases relapse risk for those with pre-existing mental illness,^187^ and especially BD in the postpartum period.^188^ So, initiatives to protect sleep perinatally are recommended in the prevention and treatment of PMAD symptoms (Level 4 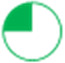). Sleep protection interventions may range from minimizing the time a mother has to be awake at night (e.g., bottle-feeding by another adult with pumped breastmilk or formula for a night-time feed to allow a longer sleep time for the mother) to evidence-based interventions to treat insomnia when it is present so that the mother can sleep when the baby is sleeping. Cognitive–behavioural therapy (CBT) for insomnia, CBT-I, is a highly effective treatment for insomnia where there is specifically effectiveness demonstrated for improving sleep quality, insomnia severity, depression and anxiety symptoms in pregnancy (Level 1 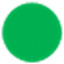), and open-label evidence for its use for insomnia in PPD specifically (Level 4 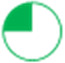).^164,189,190^ Pharmacological management of insomnia in perinatal populations in addressed in Question 6.

A systematic review of 8 trials (*n* = 231) found bright light therapy to be superior to placebo with a small-to-moderate effect size in the treatment of depression in pregnancy and postpartum, although quality of the included studies was variable, statistical heterogeneity was high and in subgroup analysis, the effect was significant only in the postpartum (5 trials) but not in pregnancy (3 trials) (Level 2 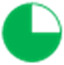).^191^ Available studies focused on maternal diet and perinatal depressive symptoms were of low or very low quality, precluding recommendations therein; specific dietary supplements are discussed in Question 8.^192^

## Question 4. What Are the Recommendations for the Use of Psychosocial Interventions?

An individual's perception of insufficient support is a strong risk factor for PMADs.^193–195^ Thus, CANMAT recommends optimizing informal supports such as those provided through community or embedded social networks of family members and friends (Level 4 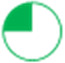). Psychosocial interventions are designed specifically to enhance perceptions of support by providing informational (i.e., psychoeducational), emotional and instrumental support.^196,197^ Psychosocial interventions that have been evaluated perinatally include home-visiting support, peer support and psychoeducational programs (summarized in Table 7). Most research is focused on perinatal depression and anxiety. Given the paucity of empirical evidence in BD, PTSD and OCD, clinicians may need to consider evidence from non-perinatal populations in the management of these disorders.

**Table 7.** Psychosocial Interventions for the Prevention and Treatment of PMADs.

Line of treatment	Depressive symptoms		Anxiety symptoms	
	Intervention	Level of evidence	Intervention	Level of evidence
**Prevention**				
**First-line**	Trained peer support^a^	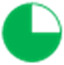	—	
**Second-line**	Co-parenting (postpartum)	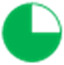	—	
**Third-line**	—		Co-parenting (postpartum)	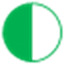
**Not recommended**	Home visits	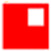	Home visits	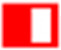
	Psychoeducational programs	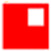	Psychoeducational programs	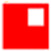
**Treatment**				
**First-line**	Trained peer support	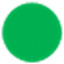	—	
**Second-line**	Listening visits (postpartum)	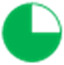	Trained peer support	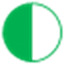
**Not recommended**	Home visits	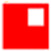	Home visits	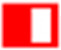
	Psychoeducational programs	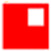	Psychoeducational programs	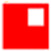
In BD, trained peer support may be helpful as adjunctive to medication treatment in the prevention of symptom recurrence, including mania and depressive symptoms (Level 4 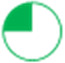 ). There is insufficient evidence for the role of structured psychoeducational programs in this population.				

*Note*. BD = bipolar disorder; PMADs = perinatal mood, anxiety and related disorders.

^a^ If risk factors for and/or subsyndromal symptoms of PMADs are present.

There is strong evidence for peer support (i.e., support by trained peers) in reducing depressive symptoms in affected individuals during pregnancy and postpartum (Level 1 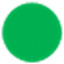).^194,198^ Peer support is effective whether delivered face-to-face, by telephone or online (virtually), in individual or group sessions. Efficacy has also been demonstrated in specific perinatal sub-populations, such as adolescents and parents of preterm infants.^198,199^ The optimal frequency for peer support appears to be at least once per week, with a length of less than 3 months being as effective as a length of 3–36 months. Peer support can also prevent PPD in at-risk populations such as those with risk factors for PPD or those with depressive symptoms that are subsyndromal (i.e., not severe enough to meet criteria for an MDE but have the potential to progress to an MDE if not addressed) (Level 2 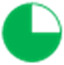).^200^Peer support may further be effective for perinatal anxiety symptoms, though additional research is warranted (Level 3 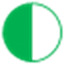).^201–203^ While there are no perinatal studies of trained peer support in BD, there is Level 2 evidence outside the perinatal period for adjunctive peer support (i.e., in addition to medication) in the prevention of symptom recurrence. Therefore, while empirical data in the perinatal population is awaited, adjunctive peer support from a peer with BD who had experienced pregnancy would be reasonable in this population (Level 4 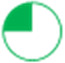).^7^

Educating patients and families about pregnancy, parenting, PMAD risks, symptoms and treatments is important and part of best practices in healthcare. However, there is an overall lack of support for specific psychoeducational programs, including group-based prenatal care, that have been evaluated for the prevention and treatment of perinatal depression, anxiety and PTSD symptoms (Level 2 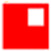 negative).^134,174,204–211^ That being said, one type of psychoeducational program, a co-parenting intervention where the focus is on educating parents and/or parental figures about how to share roles and effectively interact with each other while parenting a child together, does appear to be beneficial for the prevention of postpartum depressive (Level 2 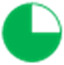) and anxiety (Level 3 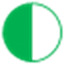) symptoms.^212^ Several structured psychoeducational programs have shown promise in relapse prevention in non-perinatal BD as adjunctive to medication treatment. However, to our knowledge no programs designed specifically for perinatal populations with BD have been rigorously evaluated.^7^

Home visiting by a nurse or other provider who provided unstructured support was generally not effective in preventing or treating perinatal depressive or anxiety symptoms (Level 2 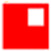 negative for depression; Level 3 negative for anxiety 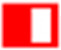).^198,213,214^ However, one type of home visiting program, structured “listening visits,” which typically involves a nurse or other home visitor (∼4–6 sessions) trained in the use of empathic listening and problem-solving, has been shown to reduce depressive symptoms in the postpartum period (Level 2 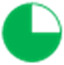).^215^ This suggests that if home-visiting programs are implemented, these should be based on specific intervention models that have been evaluated and shown to be effective.

## Question 5. What Are the Recommendations for the Use of Psychological Interventions?

Psychological treatment is recommended as a first-line option for treating moderate-severity presentations of depression, anxiety, OCD and PTSD or in mild-severity illness when lifestyle and psychosocial interventions alone are not fully effective, or are not accessible. Severe forms of these illnesses will not generally respond adequately to psychological treatments alone, and pharmacotherapy or other somatic interventions (e.g., neuromodulation) may be required to attain remission. While medications are foundational for the successful treatment of BD, adjunctive psychological interventions (i.e., in addition to medication) can be helpful in the treatment of depressive episodes, in relapse prevention and to improve quality of life.

### Depression and Anxiety

Both CBT and interpersonal psychotherapy (IPT) are effective in the prevention of perinatal depression in those with a prior history of depression, subsyndromal depressive symptoms or those with psychosocial risk factors for perinatal depression such as low socioeconomic status, single parenthood and young maternal age (Level 1 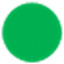).^200,205^

Psychological treatments are also effective in treating MDEs in pregnancy and postpartum, with and without comorbid anxiety symptoms (Level 1 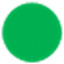).^200,205,216–222^ The modalities for which there is the most evidence for efficacy are the manualized therapies, such as CBT,^200,205,216,217,220–222^ IPT,^200,205,217,218^ mindfulness-based therapies (e.g., mindfulness-based stress reduction and mindfulness-based cognitive therapy),^216,219,222^ and behavioural activation (BA)^223^ (Level 1 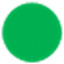). CBT and mindfulness-based treatments are also effective when specifically targeting perinatal anxiety symptoms (Level 2 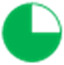).^216,219,221,222^ This includes emerging evidence for several forms of psychological treatment, primarily those containing elements of CBT, in the specific treatment of fear of childbirth symptoms (Level 2 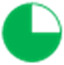).^218^

Psychological treatments for depression and anxiety are typically delivered weekly, frequently ranging from 4–16 sessions, although there is also evidence for 1-day CBT workshops in the reduction of postpartum depressive and anxiety symptoms (Level 1 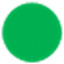).^224–226^ They are effective when delivered individually or in group format, in-person or in virtual care (i.e., by telephone or video-conference) (Level 1 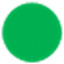).^153,155,158,160,200,205,216–222,227–236^ There is specific evidence for virtual care in ethnically and racially diverse populations (Level 1 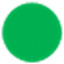).^227,234,235^ Non-specialist providers (e.g., nurses, midwives and peers)—individuals with no formal or prior experience in mental healthcare—can be trained to effectively deliver BA, CBT and IPT for perinatal depressive and anxiety symptoms(Level 1 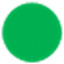).^153^

Psychological interventions can also be delivered through self-help, where the patient works through a program of therapy on their own—usually using cognitive behaviour therapy techniques—via print workbooks or online web-based modules or mobile applications. These self-help interventions are considered guided when they occur along with online or telephone support from a trained coach to encourage completion, review progress and outcomes. Self-help psychological interventions (workbook and internet-based) reduce depressive symptoms, with benefits mostly observed for guided (Level 1 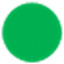 for PPD, and Level 2 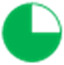 for depression in pregnancy) rather than self-guided treatments (which is thought to be at least partially because the completion rates for self-guided treatments are very low).^202,203,230,237–243^ Guided self-help interventions can also reduce anxiety (Level 2 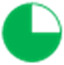).^155,158,160,216,227,231,232^ with some evidence specifically for guided iCBT in pregnancy in fear of childbirth (Level 4 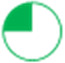).^160^ Guided self-help (internet- and non-internet based) interventions can also prevent prenatal depression and anxiety in at-risk populations such as those with risk factors for PMADs and/or subsyndromal levels of symptoms (Level 3 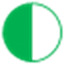).^238–243^

### OCD

Less is known about psychological treatments for PMADs other than depression and anxiety. CBT with exposure and response prevention is recommended for OCD given its well established efficacy in non-perinatal OCD,^244^ despite the fact that the volume of evidence for CBT in perinatal OCD is not extensive (Level 3 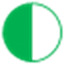).^245^

### PTSD

There has been interest in the secondary prevention of PTSD after a traumatic childbirth. Psychological debriefing interventions shortly after the event where the postpartum person is encouraged to process their emotional response to the traumatic event are not effective in the prevention of PTSD or the treatment of emergent symptoms (Level 1 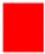 negative).^246^ This is consistent with the evidence in non-perinatal populations.^247^ However, the lack of evidence for debriefing should not deter clinicians from providing clarification to patients around the events of a birth and answering questions that patients may have. In RCTs of mothers of preterm infants with elevated PTSD symptoms, there is some positive evidence for interventions with trauma-focused CBT components for the secondary prevention of PTSD (Level 2 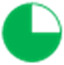).^246^ RCTs of other interventions to prevent PTSD after traumatic childbirth that were included in systematic reviews are very heterogeneous and generally of low quality, precluding the ability to make a specific recommendation about them.^246^

There are multiple psychological treatments that are effective for PTSD non-perinatally (e.g., cognitive processing therapy, prolonged exposure therapy and written exposure therapy).^248,249^ However, there is limited evidence about which treatments would be most effective for a primary target of perinatal PTSD or posttraumatic stress symptoms, particularly for symptoms of PTSD related to events that occurred in childbirth.^146,211,250^ A recent pilot study using written exposure therapy for PTSD symptoms in pregnant individuals with PTSD and substance use disorders reported promising results (Level 4 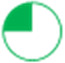).^251^

### Bipolar Disorder

Although psychological interventions for BD non-perinatally have not been shown to control symptoms on their own (i.e., in the absence of medication treatments) and have rarely been studied in the perinatal population, limited data on CBT, family-focused therapy and interpersonal and social rhythms therapy for BD in non-perinatal patients suggests some depressive symptom reduction, functional improvement and prevention of symptom recurrence when added as an adjunct to pharmacotherapy (Level 2 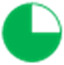). These could be considered as adjunctive interventions in pregnant and postpartum populations where feasible (Level 4 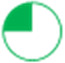).^7^

A summary of psychological interventions for the prevention and treatment of PMADs are in Tables 8 and 9, respectively.

**Table 8.** Psychological Interventions for the Prevention of PMADs.

	Line of treatment	Intervention	Level of evidence
**Depression**	First-line	CBT^a^	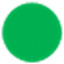
		IPT^a^	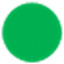
	Second-line	Guided self-help psychological interventions^a^	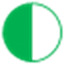
**Anxiety**	First-line	—	
	Second-line	Guided self-help psychological interventions^a^	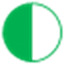
**OCD**		n.d	
**PTSD**	First-line	Interventions with trauma-focused CBT components^b^	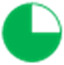
	Not recommended	Psychological debriefing after traumatic childbirth	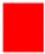
**Bipolar disorder**	First-line	—	
	Second-line	—	
	Third-line	Adjunctive CBT (relapse prevention)	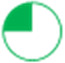
		Adjunctive family-focused therapy (relapse prevention)	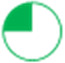
		Adjunctive interpersonal and social rhythms therapy (relapse prevention)	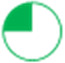

*Note*. CBT = cognitive–behaviour therapy; IPT = interpersonal therapy; OCD = obsessive–compulsive disorders; PMADs = perinatal mood, anxiety and related disorders; PTSD = post-traumatic stress disorder.

^a^ If risk factors for and/or subsyndromal symptoms of PMADs are present; ^b^After preterm birth adjunctive = adjunctive to medication treatment; n/d = no data

**Table 9.** Psychological Interventions for the Treatment of PMADs.

PMAD	Line of treatment	Intervention	Level of evidence
**Depression**	First-line	CBT^a^	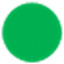
		IPT^a^	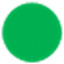
		Mindfulness-based therapies^a^	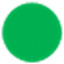
		BA^a^	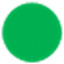
		Guided internet-based self-help including iCBT and iBA in pregnancy	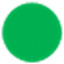
	Second-line	Guided internet-based self-help interventions including iCBT and iBA postpartum	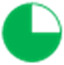
**Anxiety**	First-line	CBT for anxiety symptoms	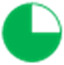
		Mindfulness-based therapies for anxiety symptoms	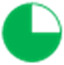
		Psychological therapies with CBT elements for symptoms of fear of childbirth	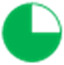
	Second-line	Guided internet-based self-help for anxiety symptoms	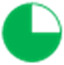
	Third-line	Guided iCBT for fear of childbirth	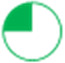
**OCD**	Second-line	CBT with exposure and response prevention elements	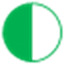
**PTSD**	Third-line	Trauma-focused psychological treatments with evidence in non-perinatal populations	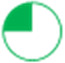
	Not recommended	Psychological debriefing after traumatic childbirth	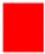
**Bipolar disorder**	Third-line	Adjunctive CBT for MDE and quality of life	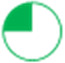
		Adjunctive family-focused therapy for MDE and quality of life	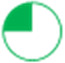
		Adjunctive interpersonal and social rhythms therapy for MDE and quality of life	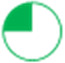

*Note*. BA = behavioural activation; CBT = cognitive–behaviour therapy; IPT = interpersonal therapy; MDE = major depressive episode; OCD = obsessive–compulsive disorders; PMAD = perinatal mood, anxiety and related disorders; PTSD = post-traumatic stress disorder.

^a^ For MDE and for those with elevated depressive symptoms (with or without MDE diagnosis).

## Question 6. What Are the Recommendations for the Use of Pharmacological Interventions?

Medications are most often used in depressive disorders, anxiety disorders, OCD and PTSD when non-pharmacological therapies are ineffective, and as first-line treatment when the initial symptoms are moderate-to-severe or severe. They may also be used as initial treatments in patients with milder symptoms who are unable to access psychological interventions, or who prefer medication over psychotherapy. Medication is the mainstay of treatment in BD and postpartum psychosis, regardless of symptom severity. While most medications used for PMADs are not perinatal-specific, brexanolone which is delivered via intravenous infusion, and its oral counterpart zuranolone, are novel medications that have recently been approved specifically for the treatment of PPD in the U.S. Brexanolone and zuranolone are neurosteroid derivatives of the progesterone metabolite allopregnanolone, and modulate Gamma-Aminobutyric Acid A (GABA-A) receptors, which are known to be involved in the pathophysiology of depression.^252,253^

Decisions about medication use during pregnancy and lactation will always involve balancing the benefits to the patient and foetus or developing infant (especially given the negative impacts of untreated or undertreated illness) against the potential risks of medication exposure in pregnancy or lactation (safety and tolerability). Some individuals will be taking medication prior to the pregnancy, so decisions centre around risk for relapse. Others will be faced with decisions about whether to initiate a medication when new symptoms arise. It is critical that clinicians have up-to-date knowledge about benefits and risks so that they can provide patients and significant others with clear, balanced and unbiased information. Decision-making can be optimized by: (a) advising patients about all of their treatment options (including not being treated), (b) helping them clarify their values and preferences about these options and (c) ensuring ample time for decision-making.^254,255^ Best practice supports describing estimates of efficacy and safety in absolute terms when such estimates are possible to provide (e.g., 1 in 1000), and acknowledging when there is uncertainty.^256^

### Medication Safety in Pregnancy and Lactation

With some exceptions, most commonly used psychotropic medications are considered fairly low risk in terms of their impact on pregnancy outcomes, including for congenital malformations (first trimester exposure), and for fetal growth, pregnancy complications, neonatal health and latent child developmental effects (e.g., motor, language and socioemotional development). When evaluating medication safety in pregnancy, it is sometimes difficult to separate the potential impacts of a medication from those of other medications or exposures (including for example pre-pregnancy exposures such as fertility treatments), the underlying illness and other factors associated with the indication for the medication (e.g., genetic risk, smoking, high body mass index and others). Because RCTs of psychotropic medications have not been conducted in pregnant people, the best available evidence is usually from large observational (e.g., case-control, cohort) studies. Unlike RCTs, observational designs can be subject to bias (e.g., confounded by the illness itself or other associated factors). Multiple methods have been used to attempt to control for confounding variables (e.g., comparing siblings born to the same parents where only one sibling was exposed to medication in-utero, comparisons to unmedicated pregnant people with the same underlying illness, etc.), so this guideline uses data from these higher-quality observational studies to inform recommendations. As observational studies cannot fully control for confounders, some degree of uncertainty about risk of medications prescribed in pregnancy will always remain.

Similarly, most psychotropic medications appear to be low-risk in lactation. In lactation, available safety evidence derives mostly from case-series and small observational studies, and there is often less information on safety available than in pregnancy. However, the majority of psychotropic medications have a relative infant dose (RID, the dose received via breastmilk relative to the mother's dose) of less than 10%, which is generally considered to be of minimal risk.^257^ A drug with an RID > 10% may still be appropriate to prescribe if it does not appear to confer risks. Caution should be exercised with preterm infants and those with renal, hepatic, neurological or other significant health conditions, particularly with agents with a long half-life or active metabolites.^258^ There is no convincing evidence that attempts to alter feed time or dump breastmilk to reduce infant exposure will reduce risks to the infant.

### Therapeutic Drug Monitoring

The dosing and monitoring protocols for most psychotropic medications are generally similar to those of the non-perinatal period. There are pregnancy-related physiological changes that can impact the metabolism of some psychotropic medications, reducing their blood levels.^259^ In most cases, clinicians should monitor patients for possible symptom worsening and adjust medication dosage as clinically appropriate should worsening of symptoms occur. However, lamotrigine and lithium elimination both increase in pregnancy with reduction to pre-pregnancy levels fairly quickly postpartum. As such, perinatal-specific monitoring protocols are advised (see Antiepileptics and Lithium below).

#### Antidepressants

Antidepressants are first-line medications in depressive disorders, anxiety disorders, OCD and PTSD and are sometimes used in bipolar depression as an add-on medication. Most studies evaluating safety in pregnancy have focused on selective serotonin reuptake inhibitors (SSRIs), serotonin-norepinephrine reuptake inhibitors (SNRIs), tricyclic antidepressants (TCAs) and other antidepressants as a whole or as a class (e.g., SSRIs), with fewer focusing on specific medications within classes. This means that, with some exceptions, it is difficult to distinguish between safety within medication classes. Data (summarized in Box 1) show that links between most antidepressants and most adverse pregnancy outcomes are reduced signficantly when confounding by indication is well-managed.^260,261^ Some experts advise tapering antidepressants near delivery to reduce the risk of poor neonatal adaptation syndrome (PNAS). While this approach is supported by evidence from a recent non-randomized case series, earlier evidence suggested that there was no difference in risk for neonatal respiratory distress between pregnancies where the antidepressant was and was not discontinued 14 days prior to delivery after accounting for maternal illness severity.^262,263^ While CANMAT acknowledges this is a developing area, the benefits of tapering to reduce the risk of PNAS likely do not outweigh the risk of worsening maternal illness severity; hence reducing or discontinuing antidepressants near delivery is not recommended (Level 4 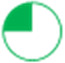).

Box 1. Summary of evidence on the safety of antidepressant use in pregnancy

*Level 3 evidence* 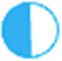 *unless otherwise specified*

Pregnancy complications: The risk of spontaneous abortion (SA) is not thought to be increased with SSRIs after accounting for maternal depression and other confounders, though there is less certainty about risk with venlafaxine, duloxetine and mirtazapine.^261,264,265^ Risks for gestational diabetes mellitus and gestational hypertension do not appear to be meaningfully increased in those taking SSRIs, SNRIs, TCAs, or monoamine oxidase inhibitors (MAOIs) during pregnancy.^261,265,266^ For postpartum haemorrhage (a concern that has been investigated due to an increased risk for bleeding with serotonergic antidepressants in non-pregnant populations), findings are mixed but the studies that have adjusted for psychiatric disorder suggest that the increase in blood loss with SSRIs and SNRIs is of small magnitude (i.e., ∼300 mL of excess blood loss) with no evidence of severe complications or mortality to the pregnant person or infant.^265,267^

Congenital malformations: SSRIs, SNRIs and TCAs as classes do not appear to increase risk for major congenital malformations.^261,268^ There may be an increased risk for cardiovascular malformations specifically during first-trimester exposure with SSRIs, SNRIs and bupropion, however this risk is thought to be very small in absolute terms (if not nil after accounting for confounders); some studies have suggested slightly greater cause for concern with paroxetine than for others in these classes.^269–272^ TCAs do not appear to be associated with meaningfully increased risk.^269^ For the MAOI tranylcypromine there are only case-series data, but severe facial dysmorphisms, cardiac malformations and fetal death have been reported (Level 4 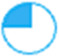).^273^

Fetal growth: While the risk of growth restriction and shortened gestational age may be increased with antidepressants generally, the magnitude of these reductions is not likely to be clinically significant (i.e., <100 grams and <1 week gestational age in most cases).^265,274–276^

Neonatal complications: Persistent pulmonary hypertension of the newborn is a rare but serious condition affecting 2/1000 infants. In foetuses exposed to SSRIs and SNRIs in the third trimester, the absolute risk may be increased to ∼3/1000 overall. For SSRIs, the elevation in risk may be lower for sertraline and escitalopram than for fluoxetine, but this is not definitive.^277^ Up to 30% of newborns exposed to SSRIs and SNRIs in pregnancy will display transient symptoms of PNAS, a constellation of symptoms that include jitteriness, respiratory distress, irritability, sleep disturbance, tachycardia and feeding problems.^278^ PNAS is self-limited in most cases, is rarely associated with severe complications, and usually resolves within 2–3 days with supportive care. This risk is increased with all SSRIs/SNRIs, but may be highest with paroxetine, venlafaxine and fluoxetine.^261^

Longer-term child health: There are no consistent associations between fetal exposure to SSRIs and later physical health (including asthma, cancer, epilepsy), neurodevelopmental (cognitive or motor problems, autism or attention-deficit hyperactivity disorder) or psychiatric problems such as anxiety or depression in adolescence.^279^ It is hypothesized that most observed associations with neurodevelopmental or psychiatric disorders in children may reflect that SSRIs are merely a “proxy exposure” for the shared genetics that place both parent and child at increased risk of these conditions (as well as the exposure to the environmental risk of parental mental illnesses).

In general, antidepressants do not have significant adverse effects on infants exposed via lactation, nor do they substantially impact breastmilk production.^280^ Fluoxetine has a longer half-life than many SSRIs and so other medications with shorter half-lives are preferred. There have also been case reports of infant seizure associated with bupropion, although these may have been due to other factors and not the medication per se. The only medication not recommended in lactation is the TCA doxepin, due to concern about infant sedation.^281^

#### Antiepileptics

Antiepileptic medications are used in the treatment of BD and certain anxiety disorders. Carbamazepine, lamotrigine and valproic acid are the only antiepileptics recommended as monotherapies in the CANMAT BD guidelines, so are reviewed here individually to the extent that it is possible to separate out specific medications from class effects. Valproic acid is associated with a substantially increased risk for infant congenital malformations (mainly neural tube and cardiac defects) after exposure in the first trimester of pregnancy and increased risk for child neurodevelopmental delay after second and third trimester exposure (Box 2).^282,283^ As such, valproic acid is *not recommended* in pregnancy. In fact, due to its known teratogenicity, it is not recommended for reproductive-aged persons of female sex generally due to the high prevalence of unplanned pregnancy. If an individual arrives in pregnancy or to a pre-pregnancy planning consultation while taking valproic acid, serious consideration should be given to whether it is possible to change the medication without substantial risk to maternal psychiatric stability. In cases where other medications have not been effective or tolerated (or if the pregnancy is nearing term and the risk for destabilization would be high with minimal reduction in risk from discontinuing at that point), clinicians and patients should fully discuss the risks and the potential benefits of continuing to maintain this medication. Although a systematic review of risks to the offspring associated with medication use in fathers and non-birthing parents was outside the scope of our guideline, it is important to mention recent literature investigating potential congenital malformation and neurodevelopmental risks associated with the use of valproate in fathers. While the European Medicines Agency's Pharmacovigilance Risk Assessment Committee reported a study showing that paternal use of valproate exposure was associated with increased risk of neurodevelopmental disorders but not congenital malformations in the offspring,^284^ 2 independent population-based studies that adjusted for important confounders did not find any association between valproate use in fathers and congenital malformations or neurodevelopmental outcomes in the offspring.^285,286^

Carbamazepine is also associated with an increased risk for congenital malformations (likely in a dose-dependent manner with greater risk as the dose increases). However, as the risk is less than with valproic acid and there is minimal concern about an association with adverse infant neurodevelopment or obstetric outcomes, it can be used with more confidence after the first trimester.^282,283^ Exposure to carbamazepine in pregnancy may increase the risk of infant vitamin K deficiency, so vitamin K should be administered in late pregnancy to reduce the risk for newborn bleeding.^287^ Carbamazepine concentrations are not significantly affected by pregnancy so dose adjustments are not typically required.^281^

Lamotrigine is the antiepileptic with the most favourable reproductive risk profile in pregnancy and lactation.^288^ However, its elimination increases as early as 5 weeks gestation and by >200% during pregnancy in ∼80% of patients so it can be difficult to attain therapeutic concentrations in a timely manner in pregnancy.^289,290^ For those already taking lamotrigine, measurement of a pre-pregnancy lamotrigine concentration can help determine the baseline therapeutic concentration. Having a target therapeutic concentration can be helpful in guiding treatment if symptoms worsen, and could be used for proactive dose adjustments in an effort to reduce risk for relapse.^291^ Lamotrigine levels return to pre-pregnancy levels within the first 2 weeks postpartum, so the dose can be tapered to the original dose associated with symptom remission prior to pregnancy.^289^ This is particularly important if the patient was taking a high dose (i.e., greater than 200 mg) prior to pregnancy, to prevent adverse side effects or toxicity.

Folic acid supplementation of at least 0.4 mg/day is recommended when antiepileptics are prescribed in pregnancy. The evidence for whether a higher dose of folic acid supplementation (i.e., 4 to 5 mg) definitely reduces the risk of neural tube and cardiac defects remains inconclusive.^292^ There has been some concern raised about whether folic acid could reduce the effectiveness of lamotrigine in non-perinatal populations with BD, so patients using lamotrigine with folic acid supplementation should be carefully monitored for potential clinical symptom re-emergence.^293^

Box 2. Summary of evidence on the safety of antiepileptic use in pregnancy (for antiepileptics indicated in the treatment of psychiatric disorders)

*All are Level 3 evidence* 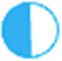 *unless otherwise specified.*

Pregnancy complications: Antiepileptics as a group are associated with a slightly increased risk for postpartum haemorrhage and induction of labour.^294^ Because these complications are also associated with epilepsy (the illness exposure primarily studied for antiepileptic exposure) independent of pharmacotherapy, it is difficult to estimate the risk for the non-epileptic pregnant patient with a psychiatric disorder. Analysis of data on antiepileptic exposure across studies suggests that these medications do not appear to increase the risk of gestational hypertension, bleeding in pregnancy, or caesarean section (Level 4 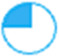).^295^

Congenital malformations: Risk for major congenital malformation is elevated with valproic acid exposure. This includes an increased risk of cardiac malformations (e.g., atrial septal defects), hypospadias, neural tube defects (e.g., spina bifida, craniosynostosis) and oral clefts.^282,296,297^ Risk of congenital malformations with valproic acid may be up to 3-fold higher or more than the general population. Carbamazepine is also associated with a slightly elevated risk for congenital malformations including neural tube defects, cardiac malformations, urinary tract abnormalities and oral cleft/palate defects, but to a much lesser degree (e.g., pooled OR 1.37).^282,296,298^ Risk for congenital malformations with lamotrigine is not significantly greater than that of the general population.^282^

Fetal growth: Antiepileptic medications in general have been associated with small absolute increased risks for small-for-gestational age and low birthweight, and with a small reduction in birthweight (∼119 g), but overall not birth height or head circumference.^299^ These risks may be increased with polytherapy of antiepileptic agents.^299^ Reduced fetal head growth may be associated with carbamazepine exposure.^300^

Neonatal complications: Antiepileptic exposure in general may increase the risk of neonatal intensive care unit admission and may be associated with increased risk for preterm birth but data are mostly in populations with epilepsy (which is associated with its own reproductive risks that may not be applicable in psychiatric populations).^294^ When lamotrigine exposure has been studied in isolation, it does not appear to be associated with neonatal complications (Level 4 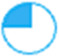).^301^

Longer-term child health: Risk for global language deficits and for intellectual and developmental disorders are increased up to 4–5 fold with valproic acid exposure and there appears to be a dose–response relationship. There may be an elevated risk for neurodevelopmental disorders with exposure to pregabalin (discussed herein as it is a monotherapy recommended in the treatment of GAD).^283,302,303^ Neither carbamazepine nor lamotrigine have been consistently associated with neurodevelopmental risks including intelligence, or mental and behavioural disorders; data are limited for other antiepileptics.^283,302,304,305^

There is ample data suggesting that there are very few adverse effects from lamotrigine exposure in lactation.^306^ Carbamazepine levels are detectable in breastfed infants although only minor adverse effects have been reported.^307^ While few adverse effects of valproic acid in lactation are reported, it is not recommended to start valproic acid in lactation due to the risk for a subsequent pregnancy unless there is a reasonable clinical rationale, patient preference and use of a method of contraception that ensures that an unplanned pregnancy would be highly unlikely (e.g., birth control implant, intrauterine device) (Level 4 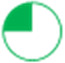).^308^ If a patient was taking valproic acid throughout the pregnancy, then it can be continued during lactation if the patient chooses to breastfeed.

#### Antipsychotics

Second-generation antipsychotics (e.g., olanzapine, quetiapine, risperidone) are a mainstay of medication treatment in BD (acute mania, depression and postpartum psychosis) and are also used in the treatment of MDD, some anxiety disorders, OCD and PTSD, mostly as adjunctive agents. Older, first-generation antipsychotics (e.g., haloperidol or loxapine) may be used in mania, severe acute agitation or postpartum psychosis (see Question 9). In general, antipsychotic dosing is similar inside and outside of the perinatal period, although the increased metabolism of pregnancy may lead to a need for dosage adjustments if symptoms re-emerge.^309^

Antipsychotics have typically been studied more as a class than individually with respect to their perinatal safety, with a few exceptions. Most data pertain to first-generation or older second-generation antipsychotic medications (e.g., olanzapine, quetiapine, risperidone) (Box 3). There is very limited information about long-acting injectable antipsychotic medications in the perinatal period, with the available evidence primarily from case reports and case series.^310,311^ Antipsychotics do not appear to be associated with an increased risk for congenital malformations, although risperidone has been linked in some, but not all analyses, with a small increased risk for cardiac malformations.^312,313^ Second-generation antipsychotics (particularly olanzapine and to a lesser extent quetiapine) may be associated with some increased risk for metabolic complications such as gestational diabetes and large-for-gestational age infants.^314,315^ Antipsychotic exposure in pregnancy may also be associated with transient delays in neurodevelopment in infancy, but does not at this time appear to be associated with longer-term child health or developmental problems.^316^

Box 3. Summary of evidence for the safety of antipsychotic use in pregnancy

*All are Level 3* 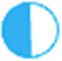 *evidence unless otherwise specified.*

Pregnancy complications: Second-generation antipsychotics with a high-risk for metabolic effects are associated with an increased risk of gestational diabetes that appears to be most significant for olanzapine (RR ∼ 1.6) and for quetiapine (RR ∼ 1.3) than for other second-generation antipsychotics.^315^ Risk for SA does not appear to be elevated for those taking first- or second- generation antipsychotics.^317^

Congenital malformations: Neither first- nor second-generation antipsychotics appear to increase the risk for major congenital malformations, although some data suggest the possibility of a small increased risk for cardiac malformations with risperidone (RR ∼ 1.3).^312,318^ Accumulating data for ziprasidone and lurasidone suggest a favourable safety profile without increased risk but more data are needed for lurasidone.^309,312,319^ Data for newer antipsychotics, such as cariprazine and asenapine, are too sparse to draw clinical conclusions.

Fetal growth: Second- but not first-generation antipsychotics are linked to large for gestational age (RR ∼ 1.6),^314^ and findings are mixed for small for gestational age and growth restriction with both types of agents.^314,320^

Neonatal complications: There does not appear to be an elevated risk for preterm birth^320^ or stillbirth.^317^ Infants exposed to first- or second-generation antipsychotics may experience symptoms in the time shortly after delivery such as respiratory distress, fussiness and gastrointestinal issues which may require neonatal intensive care admission, although the magnitude of elevated risk for these outcomes (or whether the risk for this is confounded by other factors) is unclear.^309^

Longer-term child health: Antipsychotic exposure in-utero has been relatively consistently associated with transient delays in motor development in the first few months of life that appear to resolve in the first 1–2 years (RR ∼ 1.36).^316^ Antipsychotics have not been consistently associated with long-term adverse effects on neurodevelopmental outcomes (including behavioural disorders, autism spectrum disorder, attention deficit hyperactivity disorder and intelligence quotient) or psychomotor development.^320^

All lactation safety evidence on antipsychotics stems from uncontrolled case series or observational studies (Level 4 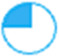). Olanzapine and quetiapine are the agents with the most reported cases in lactation and appear to have a favourable risk profile.^321,322^ Although fewer cases have been reported, risperidone and aripiprazole also appear to have low transfer into breast milk, although risperidone has been linked to more reports of adverse neonatal reactions than the aforementioned medications.^323,324^ There have been several case reports of reduced breastmilk production with aripiprazole, but data are limited. Minimal data on lactation safety are available for ziprasidone, and even less for other second-generation antipsychotic medications. Adverse effects in infants are more commonly reported for first-generation than for second-generation antipsychotics in lactation, thus more caution is recommended when using first-generation antipsychotics in lactation.^325,326^

#### Lithium

Lithium is indicated in the treatment of BD and as an adjunctive agent in the treatment of MDD. For a significant number of individuals with a history of severe bipolar I episodes, lithium may be particularly effective and so reducing or stopping it in pregnancy must be weighed against risk of severe relapse. That being said, lithium use in the first trimester is associated with a small increased risk for malformations (including the rare Ebstein's anomaly).^327^ It has also been linked to a small increased risk for some neonatal complications, including preterm birth, infant hypoglycemia, abnormal thyroid and kidney function lab values, and decreased muscle tone; data suggesting lack of longer-term neurodevelopmental impact in children are reasonably reassuring (Box 4).

Box 4. Summary of evidence for the safety of lithium use in pregnancy

*All are Level 3* 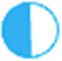 *evidence unless otherwise specified.*

Pregnancy complications: Spontaneous abortion, pre-eclampsia and postpartum haemorrhage do not appear to be associated with lithium exposure.^328–330^

Congenital malformations: During the first trimester, there is an increased risk (Relative Risk, RR ∼ 1.7) for cardiovascular malformations including Ebstein's anomaly that appears to be dose-dependent, but the absolute risk is low (i.e., an RR of 1.7 would increase the risk from 1–5 to 2–8 per 200,000 births).^331^ No associations with congenital malformations other than cardiac malformations have been clearly identified.^329^ A recent meta-analysis reported an increased risk for congenital anomalies overall, but this did not separate out the risk of cardiac malformations from the overall risk for malformations.^328^

Fetal growth: Low birth weight has not been associated with lithium exposure but some data suggest an increased risk for large for gestational age at birth (RR ∼ 2.6).^328–330^

Neonatal complications: Data from well-controlled studies do not support a risk for preterm birth.^328,330^ There may be a slightly elevated risk of hypoglycemia, thyroid abnormalities, nephrogenic diabetes insipidus and hypotonia (Level 4 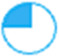)^332^ and a higher rate of 28-day neonatal readmission to special care baby units (OR ∼ 1.6).^330^

Longer-term child health: Lithium has not been associated with adverse neurodevelopmental outcomes.^316^

Pregnancy-related physiological changes such as increased cardiac output and glomerular filtration rate increase the elimination rate of lithium during pregnancy which can lead to lithium concentrations that are lower than the therapeutic range and/or the patient's pre-pregnancy therapeutic level, sometimes requiring medication adjustment.^333,334^ There are no standard guidelines for monitoring lithium levels in pregnancy. At first assessment or in early pregnancy, obtaining a baseline level is advised. Clinicians can then monitor lithium concentration once each trimester or if symptoms or side effects emerge and adjust as needed.^24,333,335^ Dose adjustments should be conservative (balancing the need for mood stabilization) to minimize any risk for adverse effects. Twice daily dosing may be necessary to prevent peak concentration side effects for the patient. Lithium should be held for any patient that develops preeclampsia due to impaired kidney function as this condition significantly increases the risk for maternal/fetal toxicity. If holding lithium for an extended period is required, an alternate mood stabilizer may need to be considered for relapse prevention. Patients with gestational hypertension, which also impairs kidney function, should be closely monitored to prevent toxicity. Because of anticipated maternal volume contraction post-delivery, the pre-pregnancy dose (or 150 to 300 mg less than the dose in pregnancy if the medication was started in pregnancy) can then be resumed immediately post-birth. Monitor the lithium level 5–7 days later, and adjust the dose as needed for symptom control and to reach therapeutic concentration. There is some controversy on whether to hold the lithium dose for 24–48 h at the onset of labour to both prevent maternal toxicity and to reduce lithium exposure levels through the umbilical cord at delivery (Box 5).

Box 5. Lithium and holding the dose at the onset of labour: reconciling conflicting evidence

Some experts advocate for holding the dose to reduce the risks for maternal toxicity (due to maternal volume contraction post-delivery) and adverse neonatal effects (due to high levels of lithium exposure via the umbilical cord).^336^ Others have expressed concerns that holding the lithium dose could increase the risk for postpartum maternal relapse, with minimal benefit of reduced exposure to the infant.^337^ While maternal volume contraction does occur post-delivery, it is unlikely that there would be immediate maternal toxicity if a dose is not held. That being said, based on the half-life of lithium in the cerebrospinal fluid, holding the dose for 24–48 h should also not significantly increase the risk of episode recurrence.^338,339^ Existing studies with infants exposed to very high concentrations of lithium (mean 1.68 mmol/L, range up to 2.6 mmol/L) showed an increased risk for adverse effects in the neonate (Level 4 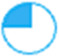).^336^ In contrast, a cohort study of 29 neonates exposed to lithium during birth did not show a significant association with adverse effects with a mean lithium level of 0.61 (SD = 0.31) mmol/L (Level 4 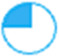).^337^ Therefore, in the absence of more definitive evidence, clinicians can consider holding the lithium dose at the onset of labour or induction (or for 24 h prior to a scheduled caesarean birth) when lithium concentration is 0.7 mmol/L or higher as this may reduce the amount of lithium in the umbilical cord without substantively increasing the risk for maternal relapse. An informed decision-making process should take place with the patient, considering both their clinical severity and risk for relapse, as well as the health of the pregnancy to date. Holding the lithium dose may not be appropriate in patients who are at very high risk for postpartum mania or psychosis, or other high-risk clinical situations such as suicide or infanticide (see Question 9).

There is also some debate around lithium use in lactation. Lithium is excreted by the kidney, so there is potential for immature infant renal function to lead to high infant lithium levels with resultant adverse effects. While some early data were concerning, it was unclear whether infant adverse effects were due to exposure in lactation, or from gestational exposure.^340^ More recent data suggest that adverse effects are limited to mild cases of hypotonia that resolve spontaneously and to transient or reversible lab abnormalities (e.g., blood urea nitrogen, creatinine, TSH).^340^ For those maintained on lithium therapy, the decision about whether to breastfeed (vs. formula feed or use donor milk) must be personalized to the risk of relapse when considering discontinuation or alternative treatment, the infant's health and the patient's preference.^341^ CANMAT does not recommend breastmilk exposure in premature infants, infants with kidney malformations, or with illnesses that may cause dehydration (Level 4 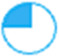). Postnatal lithium exposure via lactation may result in irritability, reduced muscle tone, or feeding difficulties. Therefore, CANMAT also recommends that infants be monitored for any change in behaviour and muscle tone and in the case of these or any other clinical concerns, lithium levels, cardiac (e.g., via electrocardiogram), thyroid and kidney function should be assessed (Level 4 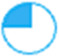).

#### Sedative-Hypnotic Medications

Benzodiazepines are sometimes used to manage acute anxiety and sedative-hypnotic medications (i.e., benzodiazepines and the hypnotic benzodiazepine receptor agonists such as zopiclone or zolpidem) are also considered a treatment option in more severe cases of insomnia. These are intended for short-term use only due to concern about developing tolerance and dependency. Safety in pregnancy and lactation must be considered with respect to their use. While not consistently linked with fetal malformations, regular use of benzodiazepines up to delivery are associated with risk of respiratory problems, somnolence and NICU admission in infants (Level 3 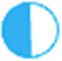).^342,343^ There are generally fewer concerns with the use of occasional (“as needed”) benzodiazepines. Hypnotic benzodiazepine receptor agonist exposure in pregnancy has not been associated with risk for congenital malformations, but may be linked to a slightly increased risk for preterm birth, low birthweight and small-for-gestational age infants (Level 3 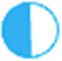).^344^ Whether there would be lower risk for these outcomes with periodic versus prolonged use is not known.

### Treatment Recommendations

To decide Line of Treatment recommendations, the CANMAT guideline committee started with relevant non-perinatal treatment guidelines, where medications are ordered by efficacy and tolerability. We used the most recently published CANMAT guidelines for MDD, OCD and BD, the World Federation of Societies of Biological Psychiatry (WFSBP) guidelines for anxiety disorders and the U.S. Department of Veterans Affairs and U.S. Department of Defense for PTSD since the most recent Canadian guidelines for anxiety disorders and PTSD were published in 2014.^345–347^ We ranked the medication options for monotherapies from these guidelines only, as there is minimal safety evidence for specific combinations of medications in the perinatal period. Based on perinatal-specific considerations, we adjusted medications up or down in Lines of Treatment for the current guideline by consensus recommendation (Box 6). In pregnancy, we considered data on: (a) congenital malformation risk (usually a concern for first trimester exposures only), pregnancy and obstetric complications, fetal growth, neonatal complications and latent developmental effects and (b) the safety of the medications in lactation since it is preferable not to have to change a medication in the early postpartum period given that this is the highest-risk time for a relapse. In the postpartum recommendations, we considered safety in lactation, and the possibility of a future pregnancy, as many parents will be planning for another child soon and unplanned pregnancies are relatively common. We also considered other aspects of maternal tolerability that are important in the postpartum. For example, if a medication causes substantial maternal sedation, it may not be ideal due to night-time infant awakenings and daytime caregiving needs unless there is additional caregiver support.

Line of Treatment recommendations are for patients initiating a new medication or those who require a medication switch because the current medication is ineffective, poorly tolerated or contra-indicated perinatally. For patients on maintenance pharmacotherapy, risk for relapse if the medication were to be discontinued or switched (and its potential implications) must be weighed against medication safety concerns.^348^ Generally, the more severe the illness (i.e., number of episodes, severity of symptoms), and the longer the time to treatment response after initiation of medication in the past, the greater the risk of discontinuing or switching medication. Importantly (see Box 6), when symptoms are well-controlled, it is not usually advisable for a medication to be changed to another one based on its placement in the Line of Treatment tables, unless it is listed as “not recommended” due to serious issues about perinatal safety. For patients taking medications where there is insufficient evidence about safety in pregnancy or lactation to be included as a first-, second- or third-line treatment, the clinician will need to consider treatment history (i.e., were other medications previously ineffective for that patient?), and severity of illness (i.e., what would be the potential risk for relapse with medication discontinuation or switch and the potential consequences of such a relapse?) to help the patient make an informed choice aligned with their values and preferences. For patients who are not taking medication at the time of presentation, but who have responded to a specific medication in the past, the best agent may be the one they have responded to previously, even if it is in a “lower” line of treatment, unless it is specifically contraindicated.

Box 6. Explanation of PMAD pharmacotherapy recommendation tables

- Options are classified into first-line, second-line and third-line treatment recommendations for each PMAD.
- Lines of treatment were created by initially considering the placement of individual agents in non-perinatal treatment guidelines, and then by adding, removing, or moving agents up or down in lines of treatment based on evidence for their safety and efficacy in pregnancy and in lactation. For example, if 2 medications were similarly effective and tolerated in non-perinatal populations, but safety data in pregnancy and lactation were more reassuring for one medication, that agent would be placed in a higher line of treatment.
- Within lines of treatment, agents are listed alphabetically. A justification for placement column contains the rationale for placing the medications within that line of treatment. Clinicians and patients can use this information to help select which agent(s) to try first within each line of treatment. Such decisions may be based on prior history or response/tolerability with previous treatments and/or how patients value the potential benefits and risks associated with specific agents.
- Although CANMAT recommends that agents listed in a higher line of treatment be tried first, there may be specific reasons for choosing an agent in a lower treatment line such as patient preference, prior treatment response or non-response, or other clinical features. Specifically:
  - In individuals on medication(s) whose symptoms are well-controlled, the medication(s) should not usually be changed to another one based on its placement in the rankings, unless the current agent is listed as “not recommended.” Switching medication may lead to clinical worsening.
  - If a medicine is taken in pregnancy, it is generally preferable to continue it postpartum to reduce the risk for clinical worsening. Significantly less medication enters breastmilk than passes through the placenta, so there would be few benefits of stopping or switching medications immediately post-delivery. Changes do not usually need to be made for lactation unless the patient is doing poorly clinically, or the medication is causing significant adverse effects in the patient or infant.

#### Considerations of Monotherapy versus Combination Medication Treatment

As the combined use of 2 or more agents during pregnancy is associated with a slightly increased risk for adverse pregnancy and infant outcomes compared with monotherapy,^349^ monotherapy treatment is preferred. Clinicians are encouraged to consider a higher dosage of a single medication (or augmenting with psychotherapy when available) before prescribing multiple medications. For those presenting on 2 or more agents, it is recommended that clinicians take a careful history of the indication for each medication and its effectiveness, to determine whether one or more medications can be safely discontinued. Caution is recommended not to expose, or maintain exposure, to medications that are not clearly effective. However, lowering the dose or minimizing the number medication exposures should not be at the expense of inadequate treatment response. In this guideline, given the minimal available safety evidence about medication combinations, Lines of Treatment are presented for monotherapies only. Because combination treatment is often needed (especially in severe depression, OCD, BD and/or postpartum psychosis), clinicians may need to use the available safety evidence for monotherapies or medication classes to inform the best approach for a specific patient.

#### MDD

Pharmacotherapy in pregnancy is reserved for individuals who have a severe MDE, those at high risk for relapse, those who are unable to access or have an inadequate response to psychotherapy, or those who prefer to use medication. For those already taking maintenance antidepressant medication, there should be counselling around the risk for relapse if medication were to be discontinued for pregnancy. Discontinuation of antidepressants in pregnancy has been associated with an elevated risk for relapse among those with a more severe depression history (i.e., multiple previous episodes, a greater severity of depression), but population-level studies suggest that those with mild-to-moderate depression severity may be not at significantly increased risk.^350^ Patients should also be counselled on risk for antidepressant discontinuation syndrome, which could be particularly unpleasant if the patient is already experiencing nausea or other physical symptoms in pregnancy. The threshold for pharmacotherapy is lower in the postpartum because the main issue is transmission to infants via breast milk, and individuals could choose not to breastfeed if they have concerns about infant medication exposure.

*Pregnancy.* Antidepressant medications are the mainstay of pharmacotherapy treatment for MDD in pregnancy (Table 10). The first-line medications—citalopram, escitalopram and sertraline—are first-line medications outside of the perinatal period, generally well-tolerated and have the most reassuring safety data in pregnancy. The second-line medications are all first-line medications outside the perinatal period, but are downgraded here as initial selections because either there are some (albeit minimal) safety concerns, there is less certainty about their safety, or tolerability is not as good as that of the first-line medications. Selection from within second-line medications can be made by clinicians and patients based on what patients prefer, and how they value the various risks, side effects and benefits presented. Paroxetine is listed as a third-line agent in pregnancy because of its links, albeit somewhat inconsistent, to a higher risk for cardiac malformations. However, it is reasonable to prescribe paroxetine if patients have had a good response to it and prefer it over other available agents. In this case, Level II ultrasonography in pregnancy is recommended to evaluate for cardiac malformations 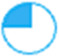. Several medications are listed as having insufficient safety data, including certain newer agents and the MAOI tranylcypromine for which the case reports that exist are of concern (Level 4 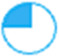).^273^ This does not mean automatic discontinuation in people who have had a good clinical response, but patients should be advised accordingly. Ketamine—increasingly being used in treatment-resistant MDD outside the perinatal period—may be particularly problematic, and likely patients should not use it during pregnancy until adequate safety data are available (Level 4 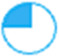).^351^

**Table 10.** Medications for Major Depressive Disorder in Pregnancy.

Line of treatment	Medication	Level of evidence efficacy		Justification for placement
		Non-perinatal	Pregnancy	
First-line	Citalopram	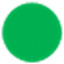	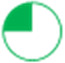	Most reassuring safety data of all antidepressants
	Escitalopram	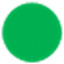	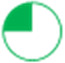	
	Sertraline	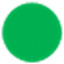	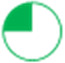	
Second-line	Bupropion	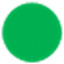	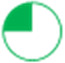	Less safety data than first-line agents, and rare case reports of infant seizure if continued in lactation
	Desvenlafaxine	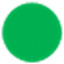	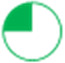	Less safety data than first-line agents
	Duloxetine	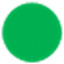	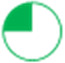	Some uncertainty about spontaneous abortion risk
	Fluoxetine	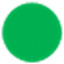	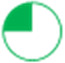	Possible higher PPHN and PNAS risk and longer half-life/higher passage into breastmilk if continued in lactation
	Fluvoxamine	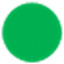	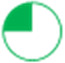	Maternal tolerability concerns at higher doses
	Mirtazapine	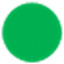	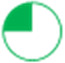	Less safety data than first-line agents and some uncertainty about spontaneous abortion risk
	Venlafaxine XR	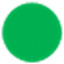	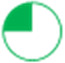	Possible higher PNAS risk Some uncertainty about spontaneous abortion risk
Third-line	Paroxetine	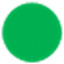	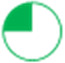	Possible higher risk for CV malformations and PNAS
	Quetiapine	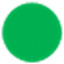	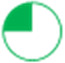	Maternal and fetal metabolic impacts, sedation
	Trazodone	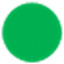	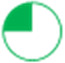	Maternal tolerability concerns at high doses
	Tricyclics^a^	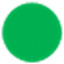	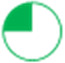	Maternal tolerability concerns
Insufficient data	Agomelatine, dextromethorphan/bupropion, ketamine, levomilnacipran, mianserin, milnacipran moclobemide, phenelzine, reboxetine, tranylcypromine (case reports of malformations and of fetal death), vilazodone, vortioxetine			

*Note*. CV = cardiovascular; insufficient data = insufficient data on safety in pregnancy/lactation; PNAS = poor neonatal adaptation syndrome; PPHN = persistent pulmonary hypertension of the newborn; SSRIs = selective serotonin reuptake inhibitors; SNRIs = serotonin-norepinephrine reuptake inhibitors.

^a^ Tricyclics (amitriptyline, clomipramine, desipramine, doxepin, imipramine, nortriptyline, protriptyline, trimipramine) are not listed individually herein as it is difficult to separate their safety within the class of medications and their tolerability issues render them as lower in the treatment line of agents (after SSRIs, SNRIs, and others) non-perinatally in any case.

*Postpartum.* Antidepressants are also the mainstay of pharmacotherapy postpartum. It is expected that the efficacy of antidepressants would be similar in the postpartum as in non-perinatal periods, but there is also some RCT evidence for the efficacy of SSRIs over placebo in treating PPD specifically (Level 2 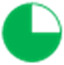).^352^ For those who are breastfeeding, there is reassuring data for several antidepressants in terms of their safety in lactation to guide recommendations about lines of treatment (Table 11). The only antidepressant not recommended in lactation is the TCA doxepin given its potential to cause excessive somnolence in exposed infants.^281^ Insufficient safety data exist for most newer agents. However, if a patient and clinician feel that the benefit of continuing that medication outweighs its drawbacks, they may select it, and close infant monitoring is recommended.

**Table 11.** Medications for Major Depressive Disorder in Lactation.

Line of treatment	Medications	Level of evidence (efficacy vs. placebo)		Justification for placement
		Non-perinatal	Postpartum	
First-line	Citalopram	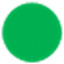	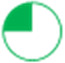	
	Escitalopram	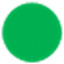	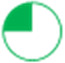	Most reassuring safety data of antidepressants
	Sertraline	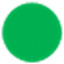	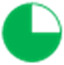	
Second-line	Bupropion	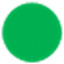	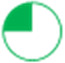	Rare case reports of infant seizure
	Desvenlafaxine	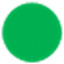	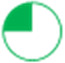	Less safety data than first-line agents
	Duloxetine	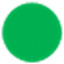	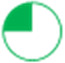	Less safety data than first-line agents
	Fluoxetine	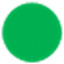	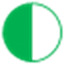	Longer half-life and higher RID than first- and second-line agents, more side effects reported, no severe concerns
	Fluvoxamine	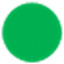	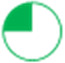	Less tolerability at higher doses
	Mirtazapine	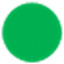	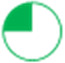	Less safety data than first-line agents and potential for maternal sedation
	Venlafaxine	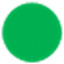	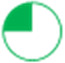	Less tolerability than first-line agents
Third-line	Paroxetine	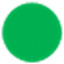	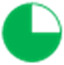	Downgraded due to possible higher risk for CV malformations in a future pregnancy
	Quetiapine	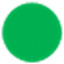	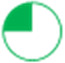	Maternal metabolic impacts, sedation
	Trazodone	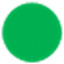	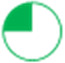	Maternal tolerability concerns at high doses
	Tricyclics (except doxepin)	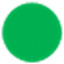	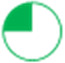 ^a^	Maternal tolerability concerns
Not recommended	Doxepin	n/a	n/a	Concern about excessive infant sedation
Insufficient data	Agomelatine, brexanolone, dextromethorphan/bupropion, ketamine, levominacipran, mianserin, milnacipran, moclobemide, phenelzine, reboxetine, tranylcypromine, vilazodone, vortioxetine, zuranolone			

*Note*. Insufficient data = insufficient data on safety in pregnancy/lactation; not recommended = not recommended due to concerns about safety in lactation; n/a = not applicable as safety concerns outweigh the value of efficacy evidence.

^a^ One trial identified in the systematic review of antidepressant efficacy for PPD (*n* = 58) found problem-solving therapy was superior to amitriptyline in the treatment of postpartum depression in Africa (Level 2 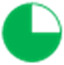).^353^

For those who are not breastfeeding, concerns about exposure to the infant do not apply, so general guidelines for treatment of MDD can be followed, also incorporating the possibility of postpartum-depression specific pharmacotherapies. Brexanolone (IV) and zuranolone (PO) (not yet available in Canada) are synthetic analogs of allopregnanolone that have been shown in RCTs to be effective for treating PPD in those who developed depression in the 3rd trimester of pregnancy or in the first 4 weeks postpartum specifically (Level 1 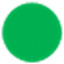).^252,253^ The onset of symptom reduction appears to be within hours for brexanolone and within days for zuranolone. However, common side effects of these medications include headache, dizziness and drowsiness or sedation, and brexanolone requires about 72 h of continuous monitoring in an inpatient setting for the intravenous infusion due to the potential for excessive sedation and sudden loss of consciousness. There is as yet an insufficient amount of safety data on these agents in lactation, so until data in lactation are available, they can be considered outside of lactation where available, feasible to administer and not cost-prohibitive.

There have been 2 small RCTs that studied psychotropic medications vs. placebo (sertraline *n* = 22, nortriptyline, *n* = 51) for preventing PPD in euthymic individuals with a history of PPD not taking medication. Sertraline was protective against PPD (Level 3 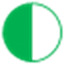) but nortriptyline was not (Level 2 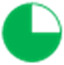).^354^ Given the uncertainty of the available evidence, clinicians are advised to consider risk for patient relapse on an individualized basis when making recommendations about whether to start (or restart) antidepressants in euthymic patients with a history of PPD in the postpartum period.

#### Anxiety Disorders

While 7 anxiety disorders exist in the DSM-5, there is currently no formally recognized, distinct perinatal anxiety disorder. For individuals presenting in pregnancy or in pre-pregnancy planning on maintenance medication therapy, the principles outlined for decision-making in MDD (see above) can be applied for anxiety disorders, as there are no data specifically on risk for relapse with discontinuation of medication in pregnancy. The potential implications of relapse should be considered based on the nature of the person's anxiety disorder, and the risks of impact of relapse or symptom recurrence on daily function.

There are no RCTs evaluating pharmacological treatments for anxiety disorders perinatally. In this guideline, formal treatment recommendations for initial medication selection are made for Generalized Anxiety Disorder (GAD), the most common anxiety disorder seen perinatally, although the principles can be applied for other anxiety disorders by consulting treatment guidelines for the other anxiety disorders along with the safety information in pregnancy and lactation presented herein. In perinatal GAD, antidepressants are also considered to be the main pharmacotherapeutic treatments, although medications from other classes are recommended in non-perinatal treatment guidelines. For GAD in pregnancy (Table 12), valproic acid is not recommended due to its teratogenicity (Box 3), nor is regular/daily benzodiazepine use (see sedative and hypnotics above). While no agents used to treat anxiety disorder in the postpartum period are absolutely contraindicated, there are inadequate data to recommend most newer agents in lactation and it is not recommend to start valproic acid due to risk for future pregnancy (Table 13).

**Table 12.** Medications for Generalized Anxiety Disorder in Pregnancy.

Line of treatment	Medication	Level of evidence efficacy		Justification for placement
		Non-perinatal	Pregnancy	
First-line	Escitalopram	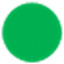	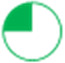	Most reassuring safety data of antidepressants
	Sertraline	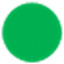	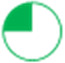	
Second-line	Citalopram	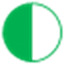	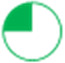	Less efficacy data than first-line agents, but reassuring safety data
	Duloxetine	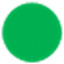	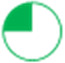	Less safety data than first-line agents, some uncertainty about spontaneous abortion risk
	Fluoxetine	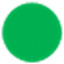	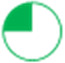	Not as much efficacy data and possible higher PPHN and PNAS risk, longer half-life and passage into breastmilk if continued in lactation
	Venlafaxine	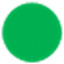	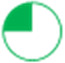	Higher PNAS risk than first-line agents, some uncertainty about spontaneous abortion risk
Third-line	Bupropion XL	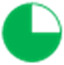	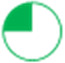	Less efficacy data than higher-ranked agents, rare case reports of infant seizure if continued in lactation
	Imipramine	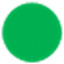	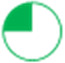	Maternal tolerability concerns
	Mirtazapine	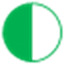	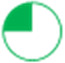	Less efficacy and safety data than higher-ranked agents, uncertainty about spontaneous abortion risk
	Paroxetine	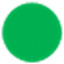	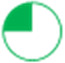	Possible higher risk for CV malformations and PNAS than higher-ranked agents
	Pregabalin	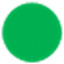	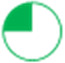	Concern for neurodevelopmental impacts
	Quetiapine	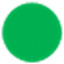	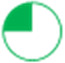	Maternal sedation and metabolic effects
	Trazodone	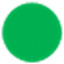	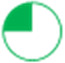	Maternal tolerability concerns at higher doses
Not recommended	Daily benzo-diazepines	n/a	n/a	Respiratory problems, somnolence and NICU admission in infants; inconsistent associations with congenital malformations; maternal dependence risk
	Valproic acid	n/a	n/a	Substantially elevated risk for congenital malformations and child developmental delay
Insufficient data	Agomelatine, buspirone, hydroxyzine, vilazodone			

*Note*. CV = cardiovascular; insufficient data = insufficient data on safety in pregnancy/lactation; not recommended = not recommended due to concerns about safety in lactation; n/a = not applicable as safety concerns outweigh the efficacy evidence; NICU = neonatal intensive care unit; PNAS = poor neonatal adaptation syndrome; PPHN = persistent pulmonary hypertension of the newborn.

**Table 13.** Medications for Generalized Anxiety Disorder in Lactation.

Line of treatment	Medications	Level of evidence for efficacy		Justification for placement
		Non-perinatal	Postpartum	
First-line	Escitalopram	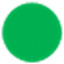	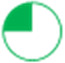	Most reassuring safety data of antidepressants
	Sertraline	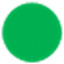	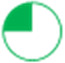	
Second-line	Citalopram	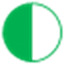	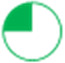	Less efficacy data than first-line agents, but reassuring safety data
	Duloxetine	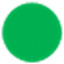	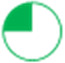	Less safety data than first-line agents
	Fluoxetine	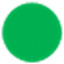	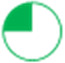	Longer half-life and higher RID than first- and second-line agents, more side effects reported in infants
	Venlafaxine	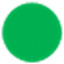	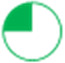	Less tolerability than first-line agents
Third-line	Bupropion XL	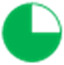	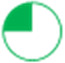	Less efficacy data, case reports of infant seizure
	Imipramine	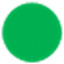	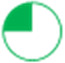	Maternal tolerability concerns
	Lorazepam	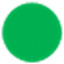	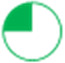	Caution for infant sedation
	Mirtazapine	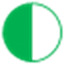	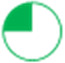	Less efficacy data than second-line agents and possible maternal sedation
	Paroxetine	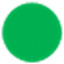	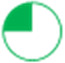	Downgraded due to possible higher risk for CV malformations in a future pregnancy
	Pregabalin	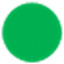	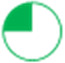	Few data in lactation, caution if future pregnancy
	Quetiapine	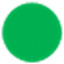	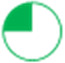	Maternal sedation and metabolic effects
	Trazodone	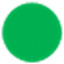	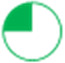	Maternal tolerability concerns at higher doses
Not recommended	Valproic acid	n/a	n/a	Concern re: starting this medication in lactation due to risk for future pregnancy. Can continue for lactation if was taking in pregnancy
Insufficient data	Agomelatine, buspirone, hydroxyzine, vilazodone			

*Note*. CV = cardiovascular; insufficient data = insufficient data on safety in pregnancy/lactation; not recommended = not recommended due to concerns about safety in lactation; n/a = not applicable as safety concerns outweigh the efficacy evidence; RID = relative infant dose.

#### OCD

There are no RCTs evaluating pharmacological treatments for OCD perinatally. The mainstays of medication treatment for OCD are antidepressants that inhibit serotonin reuptake (e.g., the SSRIs and the TCA clomipramine). In non-perinatal samples, higher doses tend to produce greater symptom improvement and remission rates than lower doses, and for some medications, dosages that exceed dose monograph recommendations may be needed.^355^ Medication recommendations for OCD are in Tables 14 and 15 for pregnancy and lactation, respectively. No first- or second-line pharmacotherapy treatments in the most recent CANMAT OCD guidelines fall into the not recommended or insufficient data category.^356^ Similar to anxiety disorders, there are no specific data on relapse risk if maintenance pharmacotherapy is discontinued, and the principles of depression management apply here as well.

**Table 14.** Medications for Obsessive–Compulsive Disorder in Pregnancy.

Line of treatment	Medications	Level of evidence for efficacy		Justification for placement
		Non-perinatal	Pregnancy	
First-line	Citalopram	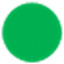	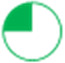	Most reassuring safety data, slightly less non-perinatal efficacy data
	Escitalopram	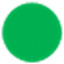	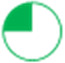	Most reassuring safety data
	Fluvoxamine	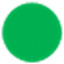	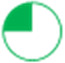	Reassuring safety data, maternal tolerability at higher doses may limit
	Sertraline	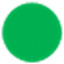	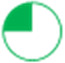	Most reassuring safety data
Second-line	Clomipramine	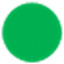	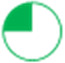	Less well tolerated than first-line agents; however, safety data are reassuring
	Fluoxetine^a^	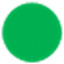	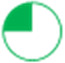	Possible higher PPHN and PNAS risk than first-line agents and longer half-life and greater passage into breastmilk if continued into lactation
Third-line	Paroxetine^b^	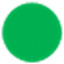	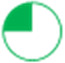	Possible higher CV malformation and PNAS risk
	Venlafaxine^c^	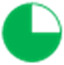	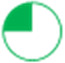	Less non-perinatal efficacy data, possible higher PNAS risk than first- and second-line agents

*Note*. CV = cardiovascular; PNAS = poor neonatal adaptation syndrome; PPHN = persistent pulmonary hypertension of the newborn.

^a^ Dosages required may go up to 80 mg; ^b^Dosages required may go up to 60 mg; ^c^Dosages required may go up to 300 mg;

**Table 15.** Medications for Obsessive–Compulsive Disorder in Lactation.

Line of treatment		Level of evidence efficacy		Justification for placement
	Medications	Non-perinatal	Postpartum	
First-line	Citalopram	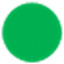	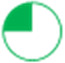	Most reassuring safety data, slightly less non-perinatal efficacy data Most reassuring safety data
	Escitalopram	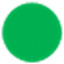	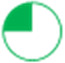	
	Fluvoxamine	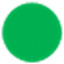	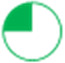	Reassuring safety data, maternal tolerability at higher doses may limit
	Sertraline	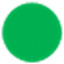	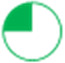	Most reassuring safety data
Second-line	Clomipramine	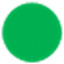	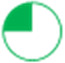	Tolerability concerns, and slightly less volume of safety data than first-line agents
	Fluoxetine^a^	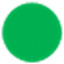	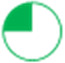	Longer half-life and higher RID than first and second-line agents, more side effects reported, no severe concerns
Third-line	Paroxetine^b^	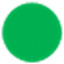	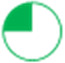	Downgraded due to possible higher risk for CV malformations in a future pregnancy
	Venlafaxine^c^	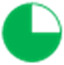	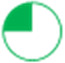	Less efficacy than first-line agents, and maternal tolerability

*Note*. CV = cardiovascular; RID = relative infant dose.

^a^ Dosages required may go up to 80 mg; ^b^Dosages required may go up to 60 mg; ^c^Dosages required may go up to 300 mg.

#### PTSD

There are no RCTs evaluating pharmacological treatments for PTSD perinatally. As such, CANMAT recommends that the use of pharmacological treatment for PTSD perinatally follows the guidance from non-perinatal clinical practice guidelines for PTSD. Non-perinatal guidelines recommend psychological therapies as first-line treatments (i.e., before pharmacological therapies) unless these are not available, severity of illness is so high that patients are not able to engage in that type of therapy without stabilization with medication, or medication is preferred.^347^ When pharmacological treatments are used, recent U.S. clinical practice guidelines (updated 2023) recommend only the antidepressant medications sertraline, venlafaxine and paroxetine, indicating that the evidence for other medications is either of low quality or that evident benefits do not outweigh potential harms or tolerability issues.^347^ Fluoxetine was at one time also in the list in a previous version of this guideline, and other guidelines.^347^ However a recent systematic review has raised questions about its efficacy.^357^ Based on these recommendations, for pharmacological treatment of PTSD in perinatal populations, sertraline is considered first-line in pregnancy, with venlafaxine as second-line and paroxetine as third-line due to its risk profile (Table 16). In the postpartum, sertraline remains the first-line treatment recommendation. If another pregnancy is not planned (and birth control measures are in place), then paroxetine may become second-line together with venlafaxine (Table 17). Prazosin is a medication that has been inconsistently recommended in clinical practice guidelines for the treatment of nightmares specifically in PTSD. CANMAT does not recommend the use of prazosin perinatally as data during pregnancy are limited with some concerning fetal effects, and there are very minimal data available on its safety in lactation (Level 4 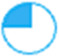).^358^

**Table 16.** Medications for Post-traumatic Stress Disorder in Pregnancy.

Line of treatment	Medications	Level of evidence for efficacy		Justification for placement
		Non-perinatal	Pregnancy	
First-line	Sertraline	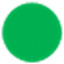	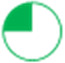	Most reassuring safety data
Second-line	Venlafaxine	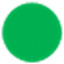	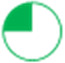	Possible higher PNAS risk than first-line
Third-line	Paroxetine	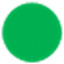	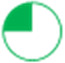	Possible higher CV malformation and PNAS risk than first-line
Not recommended	Prazosin	n/a	n/a	Concern re: fetal effects

*Note*. CV = cardiovascular; not recommended = not recommended due to concerns about safety in pregnancy; n/a = not applicable as safety concerns outweigh the value of efficacy evidence; PNAS = poor neonatal adaptation syndrome.

**Table 17.** Medications for Post-traumatic Stress Disorder in Lactation.

Line of treatment	Medications	Level of evidence for efficacy		Justification for placement
		Non-perinatal	Postpartum	
First-line	Sertraline	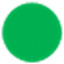	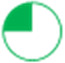	Most reassuring safety data
Second-line	Paroxetine	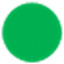	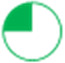	Less tolerability than first-line agent, and also downgraded due to possible higher risk for CV malformations in a future pregnancy
	Venlafaxine	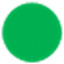	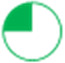	Less tolerability than first-line agent
Insufficient data	Prazosin	n/a	n/a	

*Note*. CV = cardiovascular; insufficient data = insufficient data on safety in lactation; n/a = not applicable as safety concerns outweigh the value of efficacy evidence.

#### Bipolar Disorder

Although there are no RCTs for the pharmacotherapeutic treatment of BD perinatally, the mainstay of acute and maintenance treatment for BD in pregnancy and postpartum is medication, primarily certain antiepileptics, antipsychotics and lithium. The specific medications that are advised may differ depending on the type of illness (i.e., BD type I or type II), and phase of illness, namely acute depression, mania/mixed states and maintenance treatment. In the postpartum, if not considering lactation, treatment recommendations can be based on general CANMAT BD guidelines, except for postpartum psychosis (see Question 9). Clinical pearls for prescribing in perinatal bipolar disorder are in Box 7.

Box 7. Clinical pearls for prescribing in perinatal BD

Maternal medical concerns may impact medication choices. For instance, quetiapine and olanzapine, due to concerns about metabolic impacts, may be downgraded in those who have risk factors for gestational diabetes (i.e., high body mass index, family history of type 2 diabetes), given diabetes increases the risk of pregnancy and neonatal complications. Similarly, lithium may be downgraded or not recommended in those with renal problems. For infants born preterm or with medical comorbidities, it is important to try to avoid medications with risks for adverse effects due to breastmilk exposure and those without data. If this is not an option, formula feeding is a recommended alternative to discontinuation of an effective agent. Discontinuation of effective treatment significantly increases the risk of episode recurrence in BD, particularly postpartum.

##### Maintenance

Prospective non-randomized studies in BD show that maintaining pharmacological treatment in pregnancy significantly decreases risk of symptom re-emergence (Level 3 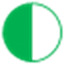).^359^ Abruptly discontinuing mood stabilizers (i.e., antiepileptics, antipsychotics, lithium) in pregnancy leads to a much more rapid relapse (∼2 weeks after stopping the treatment) versus gradual discontinuation (∼22 weeks afterward) (Level 3 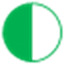).^359^ In most cases, the risk of discontinuation of medication will generally outweigh the potential safety concerns of maintenance treatment in pregnancy, even for medications that are likely to be associated with small elevations in risks for congenital malformations and other pregnancy complications (see Boxes 2–4). Patients will need to be counselled about the various benefits and risks of their medications in the context of their clinical condition and relapse risk, but it is CANMAT's opinion that most patients in remission who choose to stay on pharmacological treatment should be supported in continuing existing medication regimens in pregnancy. A notable exception to this recommendation is valproic acid. Serious consideration should be given to switching valproic acid to another medication even if it has been an effective treatment given the elevated risk for congenital malformations and neurodevelopmental problems in offspring (see Box 2).

Bipolar disorder has the highest relapse rate for any mental disorder in the postpartum period, at on average, 37%.^24^ Most relapses are non-psychotic depressive episodes, but as many as 1 in 6 (17%) will be episodes of psychosis, mania, or other severe presentations requiring psychiatric hospitalization.^24^ Risk of an acute mood episode postpartum is as high as 66% in those not taking vs. 23% in those taking medication.^24^ As such, continuation of pregnancy medications into the postpartum is strongly recommended and initiation of medication in the immediate postpartum is generally recommended to prevent relapse in those who did not take medication in pregnancy. The potential for adverse effects on the infant due to medication exposure in lactation (which will vary based on the medication) needs to be considered, as does the potential for maternal sleep deprivation. Sleep interruption is expected with a newborn but can be exacerbated when the patient is waking frequently at night to feed their infant. For this reason, plans for infant feeding must be tailored to the individual patient. Where possible, limiting the amount of patient waking overnight by pumping milk in advance or using formula or donor milk, and having a partner, family member, doula or night nurse do some or all of the nighttime infant feedings may optimize longer durations of consolidated sleep (Level 4 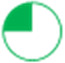).

##### Acute Treatment of Mania and Mixed Episodes

Monotherapies that are considered first-line treatment options for acute mania outside the perinatal period in the CANMAT/International Society for Bipolar Disorders (ISBD) bipolar disorder clinical practice guidelines are asenapine, aripiprazole, cariprazine, lithium, paliperidone, quetiapine, risperidone and valproic acid.^7^ In pregnancy, quetiapine (despite concerns about a small increased risk for metabolic and sedative side effects) and aripiprazole (despite fewer data being available than for quetiapine) remain first-line due to their relatively reassuring safety profiles (Table 18). Risperidone is downgraded to second-line due to a possible higher risk for congenital malformations than the previous agents. Lithium is also downgraded to second-line due to concerns about safety and complexity in its use in pregnancy, but should remain an important option to consider due to its effectiveness in preventing relapse after the acute phase. There are some data, although limited, on the safety of paliperidone in pregnancy, so it can be used but is downgraded to third-line in favour of the above-mentioned options for which there are more data available. Valproic acid is not recommended perinatally due to safety concerns. There are not enough safety data in pregnancy to reassuringly recommend other first-line medications (asenapine, cariprazine).

**Table 18.** Medications for Acute Mania in Pregnancy.

Line of treatment	Medications	Level of evidence for efficacy		Justification for placement
		Non-perinatal	Pregnancy	
First-line	Aripiprazole^a^	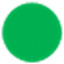	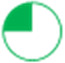	Safety data mostly reassuring, although fewer safety data than quetiapine
	Quetiapine	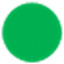	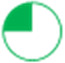	Safety data most reassuring, despite possible increased risk for gestational diabetes
Second-line	Haloperidol	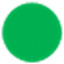	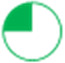	Safety data reasonably reassuring, but maternal tolerability and relapse prevention lower than first-line
	Lithium	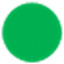	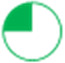	Highly effective including for relapse prevention, but elevated risk for multiple adverse outcomes and complex to manage during pregnancy (see Lithium section)
	Olanzapine	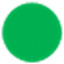	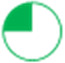	Gestational diabetes higher than quetiapine, should switch after lactation due to metabolic effects
	Risperidone^b^	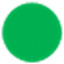	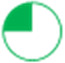	Possible higher risk for congenital malformations, not as good as lithium in relapse prevention
Third-line	Carbamazepine^b^	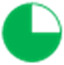	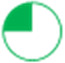	Increased risk for congenital malformations and Vitamin K deficiency
	Chlorpromazine	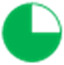	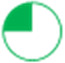	Less efficacy data than higher-ranked agents, few specific safety concerns other than class-effects
	Clonazepam	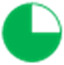	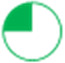	Less efficacy data than higher-ranked agents and fetal safety with exposure across pregnancy a concern
	Paliperidone	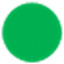	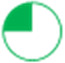	Safety data volume is small
	Ziprasidone	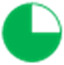	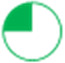	Less efficacy data than higher-ranked agents and safety data volume are minimal
Not recommended	Tamoxifen	n/a	n/a	Teratogenic potential
	Valproic acid	n/a	n/a	Substantially elevated risk for congenital malformations and child developmental delay
Insufficient data	Asenapine, cariprazine, clozapine			

*Note.* insufficient data = insufficient data on safety in pregnancy/lactation; not recommended = not recommended due to concerns about safety in pregnancy/lactation; n/a = not applicable as safety concerns outweigh the efficacy evidence.

^a^ Also first-line treatment for mixed states; ^b^Main concern is for congenital malformations so could be considered higher in the line of treatment when used after first trimester.

Olanzapine, although an excellent anti-mania agent, is second-line outside the perinatal period due to concerns about metabolic impacts. Its safety data are relatively reassuring in pregnancy, despite a possible increased risk for gestational diabetes, but it can cause sedation, so remains a second-line agent herein. If used in pregnancy, a switch to a more metabolically friendly option should be considered to prevent relapse after lactation. Haloperidol, another second-line agent non-perinatally, also remains second-line given its reasonable reproductive safety profile, although as it does not have strong evidence for relapse prevention, it would also likely need to be switched to another medication after the acute phase. Carbamazepine and ziprasidone are second-line agents outside the perinatal period, but are downgraded to third-line perinatally due to the potential for malformations and vitamin K deficiency with carbamazepine, and the limited amount of safety data available for ziprasidone. Other non-perinatal third-line medication options remain third-line perinatally, except for tamoxifen which is not recommended due to safety concerns (Level 4 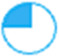).^360^ For recommendations on the management of acute agitation, see Question 9.

Monotherapies considered first-line treatments for the traditional DSM-IV definition of a mixed state in BD (i.e., full criteria for depression and mania are met at the same time) are asenapine and aripiprazole.^361^ As such, aripiprazole is the recommended first-line agent in pregnancy in this case. As yet, there are no medications that meet evidence levels to be first-line recommendations for the DSM-5 definitions of depression with mixed features or mania with mixed features.^361^

In lactation, quetiapine remains first-line for mania despite its metabolic effects and potential for sedation, as does aripiprazole although the volume of safety data is less than for quetiapine and it may reduce breastmilk. Data in lactation are also reasonably reassuring for risperidone so it can also be used as a first-line option. There is more potential for metabolic effects and sedation with olanzapine than the above-mentioned drugs, but its reasonable safety profile in lactation suggests that it can be considered a second-line option for acute mania. Switching from olanzapine to a more metabolically friendly option for relapse prevention in the longer-term would be advisable. Other potential medications are ranked accordingly based on what is known about their efficacy outside the perinatal period, and their safety in and impact on lactation (Table 19). As in pregnancy, aripiprazole is the first-line agent recommended for acute mixed states in lactation.

**Table 19.** Medications for Acute Mania in Lactation.

Line of treatment	Medications	Level of efficacy		Justification for placement
		Non-perinatal	Postpartum	
First-line	Aripiprazole^a^	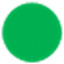	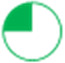	Reassuring safety data in lactation although less data than for quetiapine and may reduce breastmilk production
	Quetiapine	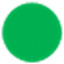	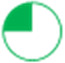	Reassuring safety data in lactation, although may cause maternal sedation
	Risperidone	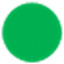	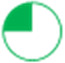	Some adverse effects in lactation (more than quetiapine)
Second-line	Carbamazepine	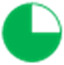	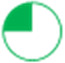	Less efficacy data than higher-ranked agents, some adverse effects reported in lactation
	Haloperidol	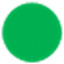	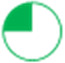	Maternal tolerability lower, relapse prevention less than higher-ranked, is linked to some adverse lactation effects, can reduce breastmilk
	Lithium	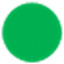	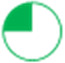	Highly effective, including for relapse prevention, but multiple concerns with lactation safety
	Olanzapine	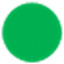	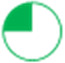	Reassuring safety data in lactation, although may cause maternal sedation (try lower doses) and will need to be switched longer-term due to metabolic effects
	Paliperidone	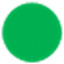	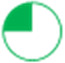	Very little known but is active metabolite of risperidone, so may have similar impact
Third-line	Chlorpromazine	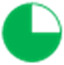	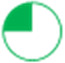	Less efficacy data than higher-ranked agents, may lead to infant drowsiness and neurodevelopmental effects, can reduce breastmilk
	Clonazepam	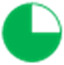	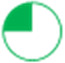	Less efficacy data than higher-ranked agents, concern about infant sedation and other developmental problems, although hard to separate from pregnancy effects
	Ziprasidone	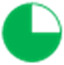	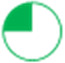	Less efficacy data than higher-ranked agents and safety data volume are minimal
Not recommended	Tamoxifen	n/a	n/a	No safety data and can suppress lactation^362^
	Valproic acid	n/a	n/a	Concern re: starting this medication in lactation due to risk for future pregnancy. Can continue for lactation if was taking in pregnancy
Insufficient data	Asenapine, cariprazine, clozapine			

*Note.* Insufficient data = insufficient data on safety in pregnancy/lactation; not recommended = not recommended due to concerns about safety in pregnancy/lactation; n/a = not applicable as safety concerns outweigh the efficacy evidence.

^a^ Also first-line treatment for mixed states.

##### Bipolar I Depression

For acute BD I depression, quetiapine and lamotrigine are first-line recommendations in pregnancy, although it may be sometimes challenging to reach therapeutic levels with lamotrigine (and thus a therapeutic effect) in pregnancy due to the significant increase in metabolism (Table 20). Lithium, a first-line agent non-perinatally, is second-line in pregnancy due to its safety and use profile. As there are minimal safety data in pregnancy for lurasidone (another first-line agent non-perinatally) it is not included in the treatment recommendations here. Neither of the 2 non-perinatal second-line options (valproic acid and cariprazine) are recommend herein due to safety concerns and insufficient safety data, respectively. While olanzapine is efficacious, it is considered a third-line option outside of the perinatal period due to the potential for metabolic side effects. In pregnancy, however, due to its relatively favourable reproductive safety profile, it is a reasonable second-line medication in pregnancy, with a plan to change to a medication with a more favourable metabolic profile after lactation. Carbamazepine is considered a third-line option based on both its efficacy profile and the higher risk for congenital malformations than lamotrigine.

**Table 20.** Medications for Acute Bipolar I Depression in Pregnancy.

Line of treatment	Medications	Efficacy level of evidence		Justification for placement
		Non-perinatal	Pregnancy	
First-line	Lamotrigine	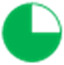	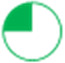	Safety data reassuring; less efficacy data than quetiapine
	Quetiapine	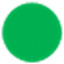	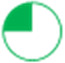	Safety data reassuring, despite possible increased risk for gestational diabetes
Second-line	Lithium	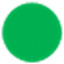	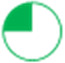	Elevated risk for multiple adverse outcomes and complex to manage during pregnancy (see Lithium section)
	Olanzapine	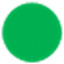	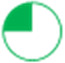	Safety data in pregnancy reasonably reassuring despite risk for gestational diabetes higher than quetiapine, should switch long-term due to metabolic effects
Third-line	Carbamazepine	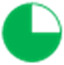	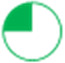	Less efficacy data than higher-ranked agents, increased risk for congenital malformations and Vitamin K deficiency
Not recommended	Valproic acid	n/a	n/a	Elevated risk for congenital malformations and child developmental delay
Insufficient data	Cariprazine, lurasidone			

*Note*. Not recommended = not recommended due to concerns about safety in pregnancy/lactation; insufficient data = insufficient data on safety in pregnancy/lactation; n/a = not applicable as safety concerns outweigh the efficacy evidence.

In the postpartum, lamotrigine is a first-line medication given that its metabolism normalizes and it has a favourable reproductive safety profile in lactation (Table 21). Quetiapine follows as a first-line treatment, due to concern about maternal metabolic effects. The remainder of the monotherapies follow the same order as the recommendations in pregnancy, for similar reasons. There is some emergent safety data for lurasidone, which has Level 2 evidence for efficacy outside the perinatal period, however, the lactation safety data are not yet clear enough to support recommending this medication. Valproic acid is not recommended due to concern about future pregnancies in the reproductive-aged population, unless there is a reasonable clinical rationale, patient preference and use of a highly-effective method of contraception.

**Table 21.** Medications for Acute Bipolar I Depression in Lactation.

Line of treatment	Medications	Efficacy level of evidence		Justification
		Non-perinatal	Postpartum	
First-line	Lamotrigine	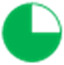	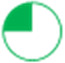	Minimal cause for concern with lactation or maternal sedation
	Quetiapine	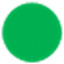	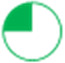	Minimal cause for concern with lactation but some maternal sedation
Second-line	Lithium	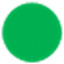	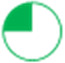	Multiple concerns with lactation safety
	Olanzapine	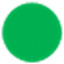	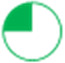	Reassuring safety data in lactation, although may cause maternal sedation (try lower doses) and may need to be switched longer-term due to metabolic effects
Third-line	Carbamazepine	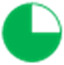	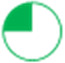	Less efficacy data than higher-ranked agents, some adverse effects reported in lactation
Not recommended	Valproic acid	n/a	n/a	Concern re: starting this medication in lactation due to risk for future pregnancy. Can continue for lactation if was taking in pregnancy
Insufficient data	Cariprazine, lurasidone			

*Note*. Insufficient data = insufficient data on safety in pregnancy/lactation; not recommended = not recommended due to concerns about safety in pregnancy/lactation; n/a = not applicable as safety concerns outweigh the efficacy evidence.

##### Bipolar II Depression

For acute BD II depression, recommendations based on non-perinatal efficacy suggest quetiapine should be first-line, and lamotrigine and lithium second-line due to their more limited efficacy. We also consider lamotrigine as a reasonable first-line agent in pregnancy due to its favourable safety profile (Table 22). Due to its potential adverse effects, lithium remains second-line. In the postpartum, as quetiapine is associated with sedation, lamotrigine might be preferred for some patients. As such, in lactation and in the postpartum generally, we recommend either quetiapine or lamotrigine as first-line options, with lithium remaining second-line as it is in non-perinatal populations (Table 23).

**Table 22.** Medications for Acute Bipolar II Depression in Pregnancy.

Line of treatment	Medications	Efficacy level of evidence		Justification for placement
		Non-perinatal	Pregnancy	
First-line	Lamotrigine	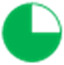	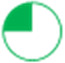	Safety data reassuring; less efficacy data than quetiapine
	Quetiapine	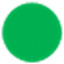	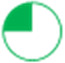	Safety data reassuring, despite possible increased risk for gestational diabetes
Second-line	Lithium	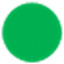	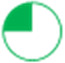	Possible higher risk for cardiovascular malformations and other fetal impacts
	Sertraline^a^	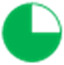	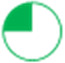	Safety data reassuring (caution for possible antidepressant-induced mania)
Third-line	Fluoxetine^a^	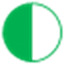	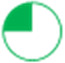	Safety data less reassuring than sertraline, possible higher PPHN and PNAS risk than sertraline (caution for possible antidepressant-induced mania) and longer half-life and greater passage into breastmilk if used in lactation
	Venlafaxine^a^	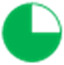	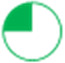	Safety data less reassuring than sertraline, possible higher PPHN risk than sertraline (caution for possible antidepressant-induced mania)
	Ziprasidone^b^	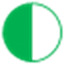	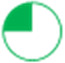	Safety data volume are minimal
Not recommended	Paroxetine	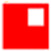	n/a	n/a
	Valproic acid	n/a	n/a	Elevated risk for congenital malformations and child developmental delay

*Note*. Not recommended = not recommended due to concerns about safety in pregnancy/lactation; n/a = not applicable as safety concerns outweigh the efficacy evidence; PNAS = poor neonatal adaptation syndrome; PPHN = persistent pulmonary hypertension of the newborn.

^a^ Pure, non-mixed depression; ^b^Depression and mixed hypomania.

**Table 23.** Medications for Acute Bipolar II Depression in Lactation.

Line of treatment	Medications	Efficacy level of evidence		Justification for placement
		Non-perinatal	Postpartum	
First-line	Lamotrigine	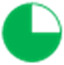	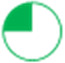	Less efficacy data than quetiapine; minimal cause for concern with lactation or maternal sedation
	Quetiapine	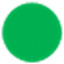	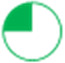	Effective, minimal cause for concern with lactation but some maternal sedation
Second-line	Lithium	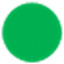	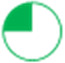	Effective but multiple concerns with lactation safety
	Sertraline^a^	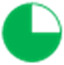	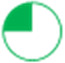	Less effective than higher-ranked agents, safety data very reassuring in lactation (caution for possible antidepressant-induced mania)
Third-line	Fluoxetine^a^	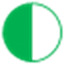	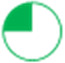	Less effective than higher-ranked agents, longer half-life and higher RID than first and second-line agents, more side effects reported, no severe concerns (caution for possible antidepressant-induced mania)
	Venlafaxine^a^	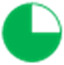	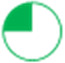	Less tolerability than higher-ranked agents (caution for possible antidepressant-induced mania)
	Ziprasidone^b^	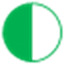	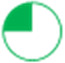	Less effective than higher-ranked agents, safety data volume in lactation are minimal
Not recommended	Paroxetine	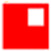	n/a	n/a
	Valproic acid	n/a	n/a	Concern re: starting this medication in lactation due to risk for future pregnancy. Can continue for lactation if was taking in pregnancy.

^a^ Pure, non-mixed depression only; ^b^Depression and mixed hypomania. RID = relative infant dose.

Antidepressant monotherapy is sometimes carefully used with close monitoring in the case of depression without mixed features outside the perinatal period even though it confers some risk for antidepressant induced mania or mixed states. Due to the elevated risk for postpartum psychosis among those with BD (even BD II) this is not an ideal strategy perinatally, especially in the first 3 months postpartum and especially as monotherapy without a mood stabilizing medication (see Question 9). That being said, sertraline, given its relatively favourable reproductive safety profile in pregnancy and lactation, could be a second-line treatment in pregnancy and in lactation, although it should likely be used after the potentially more effective second-line options. Clinicians should provide education to patients (and partners when possible) about the possibility of emergent manic or mixed symptoms, and develop a plan, including how and when to seek urgent assistance.

Third-line treatments are the other antidepressants with some efficacy data in BD II depression (fluoxetine and venlafaxine), but that have a less favourable reproductive safety profile than sertraline (see antidepressants section and tables for MDD recommendations above). The same precautions about risk for postpartum psychosis would apply as were described above for sertraline. Of the third-line monotherapies in non-perinatal populations, ziprasidone has some reassuring safety data in pregnancy and lactation so is a possibility. Valproic acid is not recommended in either pregnancy or postpartum due to safety concerns.

## Question 7. What Are the Recommendations for the Use of Neuromodulation?

Neuromodulation (or neurostimulation) interventions comprise a group of treatments that affect the central nervous system via electrical or magnetic stimulation. In non-perinatal populations, non-invasive interventions such as transcranial direct current stimulation (tDCS) and repetitive transcranial magnetic stimulation (rTMS) are generally used in acute MDEs when initial treatments have not been successful.^7,11^ They are delivered daily or multiple times daily over a course of several weeks, which has limited their uptake for some patients. These non-invasive neuromodulation interventions have begun to receive some attention in the perinatal period.

Several RCTs of rTMS for PPD suggest efficacy with minimal maternal side effects, mostly when used as an adjunctive treatment in cases of incomplete response to antidepressant medication; however, the quality of the evidence is limited in most trials (Level 2 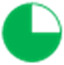

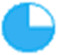
).^363–365^ Given the strong evidence for its use non-perinatally, adjunctive rTMS is recommended for PPD.^11^ Adjunctive tDCS is recommended as a third-line option for mild-to-moderate PPD, as further evidence to substantiate its use is required in the perinatal population.

There has been one small RCT of rTMS for unipolar depression in pregnancy (monotherapy and as an adjunct to antidepressant medication; *n* = 26); although 3 of eleven participants receiving rTMS experienced late preterm birth versus none in a sham-control group (Level 3 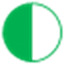

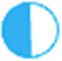
).^366,367^ There has also been a small-sample RCT (*n* = 20) of tDCS as monotherapy for unipolar depression in pregnancy that generated promising results (Level 3 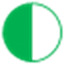

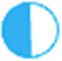
).^368–371^ Since safety data on rTMS and tDCS in pregnancy remain limited, these are not recommended as routine treatments for depression in pregnancy at this time. No recommendations can be made at this time for the use of rTMS or tDCS perinatally for anxiety, OCD, PTSD or BD.

ECT, which is typically delivered 2–3 times a week, requires a general anaesthetic and is associated with more adverse effects than less invasive treatments, is also included in this category. In non-perinatal populations, ECT is generally used in severe depression, mania, psychosis and/or acute suicidality.^7,11^ Maintenance ECT is vital in some cases of refractory depression, and ECT may work particularly quickly in treating acute psychotic depression and in treating mania, so its use is clinically important in the perinatal period as well.

Studies on ECT safety in the perinatal period are limited to case studies or series, often with no comparison groups, and there are no RCTs.^372,373^ Reports of ECT use in pregnancy are limited to severe depression, psychosis and mania (with and without medications). ECT appears relatively safe even when administered during the first trimester of pregnancy. Adverse effects reported in pregnancy include fetal arrhythmia, fetal bradycardia, premature birth, abdominal pain, uterine contractions, vaginal bleeding, placental abruption, threatened abortion and fetal death, but it is difficult to quantify risk attributable to ECT in these high-risk cases (Level 3 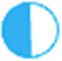).^370,372,373^

Based on these considerations, ECT is an appropriate first-line intervention for acute depression with severe psychotic or catatonic features, acute suicidality or deteriorating physical condition in pregnancy. ECT is also recommended for severe presentations of depression, mania, mixed states or psychosis, when pharmacological treatments are not successful. ECT in pregnancy should occur in close collaboration with anaesthesia and obstetrical services. While most general anaesthetics and muscle relaxants used in ECT administration are considered compatible with pregnancy, several strategies to optimize safety of the patient and foetus have been recommended.^374^ These include administering anti-nausea agents prior to the procedure, adding sodium citrate (oral) to prevent aspiration, avoiding hyperventilation prior to induction (as pregnancy is already a state of mild hyperventilation), elevation of the right hip (especially after 20 weeks gestation) to avoid aortal-caval compression of placental blood flow and fetal monitoring before, during and after procedure (including continuous fetal monitoring throughout the procedure and for some time afterward once it is possible to do so based on the number of weeks gestation). Intubation may be required depending on the stage of pregnancy to avoid the risk of aspiration.

Safety concerns with ECT are less in the postpartum relative to pregnancy. It can be used for severe cases as in non-perinatal populations with consideration given to the perinatal context (e.g., needs for assistance with childcare). It is generally recommended that patients can resume breastfeeding immediately after anaesthesia as the passage of anaesthetic medications into the breastmilk is minimal.^375^

## Question 8. What Is Recommended for the Use of Complementary and Alternative Treatments?

CAM treatments are increasingly being studied for depression and anxiety in perinatal populations.^11^ Research on CAMs perinatally is mostly in populations with mild-severity depressive episodes (PPD or MDE in pregnancy) or elevated depressive or anxiety symptoms based on standardized symptom scale cut-off scores. The methodological quality of the available literature is typically low, precluding the ability to make recommendations for any CAMs as first-line treatments for the perinatal context.^376^ Further, evidence on the use and effectiveness of some CAMs, such as yoga, acupuncture (a key component of traditional Chinese medicine), and consuming specific foods and supplements, has been culturally- or country-specific, so may not necessarily apply outside of the settings in which evaluation occurred. However, it is clinically reasonable to consider the use of CAMs that have promising evidence for efficacy and minimal evidence for harm as adjunctive treatments and/or in individuals who prefer the use of CAMs.

Yoga can prevent depressive symptoms in pregnancy, and can reduce depressive symptoms in pregnancy and in PPD (Level 2 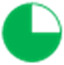); the evidence is mixed for the treatment of individuals with a clinical diagnosis of MDE in pregnancy, and for prevention and treatment of anxiety in pregnancy.^172,178,237,377–391^ Acupuncture as an adjunct to the standard of care has evidence in reducing depressive symptoms postpartum and in PPD specifically (Level 2 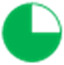).^392^ Massage therapy in pregnancy (in some studies as an adjunct to the standard of care) is effective in decreasing depressive symptoms in mild-severity MDE and in reducing anxiety symptoms (Level 2 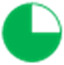).^237,385,386,393,394^ Music therapy in the postpartum as an adjunct to the standard of care decreases depressive symptoms in individuals with mild-severity PPD (Level 2 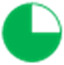).^395,396^ Beneficial effects have also been found for reduction of perinatal depressive and anxiety symptoms (Level 2 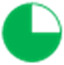) but there is not enough evidence to recommend it for prevention of depression or anxiety symptoms (Level 4 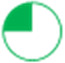).^209,237,384,385,395–400^

Multiple RCTs have examined the effectiveness of macronutrients and micronutrients for treating perinatal depression and anxiety. In the single positive trial, 1000 IU of Vitamin D taken daily for 6 months postpartum led to improvement in depressive symptoms (Level 2 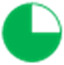).^401^ In mild-severity PPD, 8 weeks of saffron (15 mg po bid) led to depressive symptom improvement in one small-sample trial with a very high risk of bias (Level 3 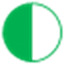).^402^ Trials of Omega-3 fatty acids for the treatment of perinatal depression have had mixed results, so a recommendation cannot be definitively made about their use at this time.^403^

Although some of these nutrients may be used for other purposes in pregnancy or postpartum (e.g., for protection against neural tube defects in pregnancy with folic acids, or treatment of anaemia with iron supplements), CANMAT does not recommend the use of folic acids, high flavonoid foods, nor iron, selenium, calcium, zinc, magnesium or copper for the prevention or treatment of depression in pregnancy or PPD because these interventions have generally not be shown to be effective for these conditions (Level 2, 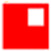 negative).^403^ St. John's Wort is also not recommended in pregnancy or in lactation based on non-controlled, case-series data suggesting a higher-than-usual risk for adverse outcomes (Level 4 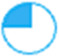).^404,405^ There is insufficient evidence to provide recommendations for aromatherapy,^237,387,400,406–410^ relaxation,^237,378^ hypnosis^174^ or probiotics (Level 4 negative for treatment of depression and anxiety in pregnancy for the last three interventions in this list 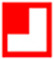).^411,412^

A summary of recommendations for CAM treatments for depression and anxiety symptoms are in Table 24.

**Table 24.** Complementary and Alternative Medicine Treatments for Depression and Anxiety Symptoms.

Line of treatment	Depression symptoms		Anxiety symptoms	
	Intervention	Level of evidence	Intervention	Level of evidence
**Prevention**				
**First-line**	—		—	
**Second-line**	Yoga in pregnancy	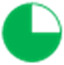	—	
Insufficient or mixed evidence	Aromatherapy postpartum Music postpartum		Music postpartum Yoga in pregnancy	
**Treatment**				
**First-line**	—		—	
**Second-line**	Adjunctive acupuncture postpartum^a^	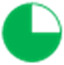	Massage therapy in pregnancy	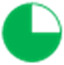
	Massage in pregnancy	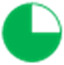	Adjunctive music in pregnancy and postpartum	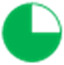
	Adjunctive music in pregnancy and postpartum^(a for postpartum)^	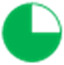	—	
	Vitamin D in postpartum	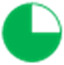	—	
	Yoga in pregnancy and postpartum^(a^ ^for postpartum)^	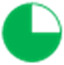	—	
**Third-line**	Acupuncture in pregnancy^a^	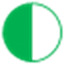	Massage therapy postpartum	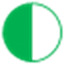
	Massage in pregnancy^a^	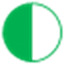	Relaxation in pregnancy	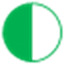
	Saffron postpartum^a^	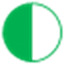	—	
**Not recommended**	Folic acids, high flavonoid foods, nor iron, selenium, calcium, zinc, magnesium, or copper	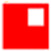	—	
	St. John's Wort	Safety concerns	—	
**Insufficient or mixed evidence**	Aromatherapy postpartum Hypnosis Omega-3 fatty acids Probiotics in pregnancy Relaxation Yoga for MDE in pregnancy		Aromatherapy postpartum Hypnosis Probiotics in pregnancy Relaxation Yoga in pregnancy	

*Note*. The level of evidence for saffron postpartum was downgraded to Level 3 due to its significant high risk of bias. Given the low quality of the evidence and the difficulty in classifying it according to the standards described, all interventions started below first-line. Adjunctive = adjunctive to the standard of care; MDE = major depressive episode.

^a^Also effective in mild MDE.

## Question 9. What Are the Recommendations for Managing High-Risk Clinical Situations?

When providing care for PMADs, clinicians should always assess a patient's risk to themself or others, including the foetus, infant or older children. Clinically high-risk symptom presentations may include acute psychotic episodes (e.g., postpartum psychosis), periods of increased acute risk of suicide, acute intoxication or agitation, and other situations where there are thoughts about or evidence of potential for harm to self or others, including child abuse or neglect.

High-risk clinical symptom presentations can occur regardless of past psychiatric history but individuals with a previous psychiatric history are at increased risk. In assessing safety, particular attention should be paid to the following:

- Inquire about suicidal thoughts, intent and/or plan, including access to means (e.g., medications for intentional overdose, firearms, knives).
- Inquire about the presence of a safety plan and other strategies to mitigate risk.
- Inquire about thoughts about harming the infant or others, and distinguish intrusive, ego-dystonic thoughts about harm from intent to harm.
- Assess for psychotic symptoms, including delusions, hallucinations and/or disorganized thoughts or behaviours.
- Inquire about and observe for signs of inability of the patient to take care of themselves and/or others, including child neglect or abuse.
- Pursue collateral history, for instance from family members, to clarify the level of risk, and assess family/social support capacity to care for the patient and the infant as well as the availability and use of other supports.

In any high-risk situation, referral to expert mental health providers is warranted. Emergency services may be required, which can be accessed by calling 9-1-1 or 9-8-8 (mental health crisis line) in Canada and the U.S. and/or sending a patient to the emergency department if there is a concern about imminent harm to the patient or others. Clinicians should follow their local professional practice guidelines, laws and policies with respect to involuntary hospitalizations and when to report to child protection services.

### Postpartum Psychosis

Postpartum psychosis occurs after about 1–2 in 1000 births and is a very high-risk perinatal mental health condition due to its association with risk of maternal suicide and infanticide.^413^ “Postpartum psychosis” is a term that is generally used to refer to a specific unique mix of mood and psychotic symptoms that are strongly associated with BD.^414,415^ Symptoms typically start within the first week after delivery, and almost always in the first 4 weeks postpartum, although they can also begin later in the postpartum period.^416^ They can include delusions, hallucinations, irritability, mood swings/emotional lability, catatonia, agitation and/or hyperactivity. Disorientation, depersonalization and derealization can occur, but are less common than the mood symptoms.^416^ There can be rapid fluctuation in symptoms, including changes in the level of consciousness in a delirium-like presentation. This can make diagnosis complicated as there may be fluctuation in and out of psychotic states. In some cases, patients may present signs of impaired attachment with their offspring.

When considering a diagnosis of postpartum psychosis, it is important to consider that psychotic symptoms such as delusions, hallucinations and disorganization of thoughts and behaviours can occur in several contexts. The DSM-5-TR allows the use of a “peripartum onset specifier” to characterize episodes of psychosis across various diagnoses (e.g., MDD with psychotic features, BD). Psychotic symptoms can also be seen in the context of a primary psychotic disorder such as schizophrenia or schizoaffective disorder, with acute intoxication, or more rarely due to a general medical condition. There is a need to consider this differential diagnosis. Treatment recommendations will differ if a better explanation for the presentation would be a major depression with psychotic features, acute intoxication or a psychotic episode due to a general medical condition, instead of a postpartum psychosis. There should be a full physical and neurological examination and basic laboratory testing including urine toxicology, to rule out any organic cause. Additional laboratory tests and/or the use of EEG or neuroimaging scans may be indicated when there is clinical suspicion of epilepsy/seizures, traumatic brain injury or other serious medical conditions, including those that can cause neuropsychiatric symptoms (e.g., anti-NMDA receptor encephalitis or posterior reversible encephalopathy syndrome).

*Prevention.* The strongest risk factors for postpartum psychosis are a personal and family history of BD, and a previous history of postpartum psychosis.^24,416–418^ As such, individuals with a personal (or familial) history of BD or postpartum psychosis should be provided with psychoeducation about their risk during or even before pregnancy. Sleep deprivation may precipitate psychotic symptoms in this group, although a reduced need for sleep can also be an early sign of psychotic mania.^419^ A treatment plan aimed at the prevention of psychosis should be offered, including consideration of prophylactic medication treatment and strategies to protect sleep (Level 4 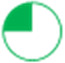).^419^ Lithium appears to have the best evidence in the prevention of postpartum psychosis (likely in cases of a past history of postpartum psychosis and bipolar I disorder) (Level 4 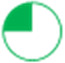).^420^ However, those with BD in remission on appropriate antimanic or antipsychotic treatment in pregnancy should continue their maintenance pharmacotherapy into the postpartum (Level 4 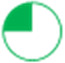). Regardless, close monitoring is required in the early postpartum to identify early emerging symptoms. A longer hospital stay for monitoring and implementation of sleep protection strategies may also be considered, especially if the patient displays initial signs of mental distress and/or does not have appropriate social supports.

Primiparity as well as pregnancy and delivery complications, including pre-eclampsia, have also been identified as factors associated with postpartum psychosis, although most primiparous people and those with obstetrical complications do not develop it, so no specific recommendations are made in this regard.^24,416–418^

*Management.* Postpartum psychotic symptoms require urgent assessment and almost always require immediate treatment, in an in-patient treatment setting. It is essential to assess risk to self, the newborn, other children in the home and other family members. Given the acute risks associated with postpartum psychosis, it is not uncommon that breastfeeding will need to be paused or discontinued for the benefit of both the patient and the baby. Clinicians need to provide psychoeducation and ongoing support for patients, family and other members of the patient's support system. Individuals with possible psychosis and thoughts of harming themselves and/or the baby should not be left alone, especially with the baby, until a comprehensive risk assessment is completed (Level 4 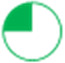). Safety planning and the creation of a plan of action with the patient and their support system including active ongoing involvement of mental health professionals should be formulated, closely monitored, and maintained until the psychotic symptoms fully subside (Level 4 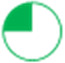).^213^

Postpartum psychosis treatment should be individualized to address the primary disorder and possible contributing psychiatric comorbidities, and to prevent recurrence of psychosis. For acute treatment, the initial recommendation is to start antipsychotic medication with or without an intermittent benzodiazepine (such as lorazepam) to manage insomnia and/or to decrease agitation (Level 4 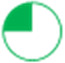).^420^ Lithium may be added to enhance treatment response and also due to evidence for its efficacy in relapse prevention, taking into account patient preference and considerations about lithium in lactation (Level 4 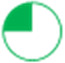) (see Question 6).^420^ ECT is an option if rapid treatment is required, if there is insufficient response to pharmacotherapy, or if there is a past history of response to ECT (see Question 7).^420^ In the context of known BD, treatment of acute postpartum psychosis must take into account prior response to medications. In this case, lines of treatment for acute mania in the BD section can then be followed (see Question 6).^7^

After the acute episode of postpartum psychosis is treated, patients will require careful ongoing monitoring and follow-up as outpatients, preferably in mental health specialty care for a minimum of 6 to 12 months, with the antimanic agent maintained for a minimum of 12 months for relapse prevention (Level 4 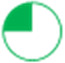).^420^ Optimizing social support and involving the partner, carer and/or support system in the post-acute phase are also important (Level 4 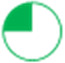).^420^ Peer support may be a key resource during recovery (Level 4 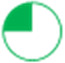).^420^

### Acute Agitation

Acute agitation—that is, ranging from self-reported symptoms to observable impatience, restlessness and aggression—in PMADs would be expected to occur mainly in the context of mania or psychosis, but can also occur in acute interpersonal crises, or other etiologies that should be managed accordingly (e.g., a medical condition, substance intoxication or withdrawal).

There are no RCTs on the management of acute agitation in PMADs. A sequential approach to management is recommended from the least to the most restrictive (Level 4 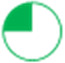),^421,422^ as follows: The first step for cooperative persons is verbal de-escalation. The next step is offering oral medication. In this case, a benzodiazepine can be used, with lorazepam preferred over longer-acting benzodiazepines. Monotherapy is preferred but if the agitation is more severe, especially in the context of psychotic symptoms, an antipsychotic can be combined with a benzodiazepine. If an individual has already started on an antipsychotic, then the dose can be optimized. In some cases, an antipsychotic can be prescribed on an “as needed” basis. Olanzapine is supplied in a rapid dissolving form as well as intramuscular. Quetiapine is sedating but has a slower onset of action and is only available in oral form. Ziprasidone has also been suggested as an alternative for a quick response, but has less safety data perinatally.^423^ Typical antipsychotics are less favoured in general psychiatry but haloperidol (available in oral, intramuscular or intravenous forms) has minimal perinatal risk so it can be used; loxapine is also a reasonable option but less is known about its safety perinatally than for haloperidol.^424^ If there is imminent risk of violence, physical restraint may be necessary. In this case, special care must be taken with positioning, especially in the second or third trimesters of pregnancy, when using 4-point restraints, to avoid compression of the vena cava. Physical restraints should be used for the shortest amount of time possible, and with fetal monitoring if available (Level 4 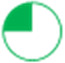).^421^

### Risk of Suicide

Suicidal thoughts are estimated to occur in as many as 8% of pregnant people and birthing parents.^425^ Most people who experience suicidal thoughts do not attempt suicide, and in many cases, being pregnant or having a child is a protective factor against suicide.^426^ However, although maternal mortality is relatively rare, suicide is a leading cause of death in the perinatal period according to data from several countries.^427^ The highest risk for suicide appears to be during acute episodes of severe depression, mania/mixed states, psychosis and/or substance or alcohol intoxication. Inquiry about suicidal thoughts should be part of mental health assessments and follow-up in all individuals with PMADs. Inquiring about or monitoring suicidal thoughts does not increase the risk for suicidal thoughts or actions. Some of the most commonly used standardized clinical questionnaires (e.g., EPDS, PHQ-9) contain an item focused on suicidal thoughts, so these can be used to aid in inquiry.

In the absence of perinatal-specific evidence, it is recommended that the assessment and management of suicide risk is similar to that in non-perinatal populations (Level 4 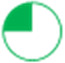).^11^ Assessment involves asking detailed questions about the presence, frequency and persistence of suicidal thoughts, whether there are specific plans for suicide, intent, or means to carry out a plan, and the potential for lethality. For instance, individuals with specific plans, intent and means, especially high-lethality means, would be considered much higher risk than those without these characteristics. Suicidal thoughts in the context of psychosis or acute intoxication would also be high-risk situations. It is important to always ask specifically about whether there is intent to end the life of their foetus or infant.

In the management of suicide risk, optimizing treatment for the underlying PMAD is key. For individuals at high or imminent risk for suicide, hospitalization is required (involuntarily if needed). For those not hospitalized, management requires careful vigilance, optimization of social supports and close monitoring by partners, family members and care providers. Prevention considerations include removing access to lethal means (e.g., removal of sharp objects, oversight of medications due to risk of misuse or overdose, etc…). Clinicians can work with patients and their support systems to identify warning signs and create a list of coping strategies and resources to access if suicidal thoughts arise (e.g., resources for 24/7 support, such as crisis lines including 9-8-8 in the U.S. and Canada, and the closest emergency services). Safety plans should be revisited in every follow-up appointment and modified as needed.

### Thoughts of Infant-Related Harm

Thoughts of infant harm are common in the postpartum period.^428^ In most cases, thoughts are intrusive and unwanted and do not represent a safety concern. When care providers receive a disclosure of thoughts of harming one's infant, the thoughts should be fully assessed before referral to child protection in case the latter is unnecessary. Recurrent unwanted intrusive thoughts or images of harming an infant on purpose (intentionally) that cause distress to the mother, with no actual intent or desire to harm the infant (i.e., “ego-dystonic” thoughts) may be best conceptualized as obsessional symptoms, with low risk of infant harm.^428^ These ego-dystonic thoughts are very distressing, and many patients—especially those who are from population groups that have a history of experiencing discrimination in health care—may conceal them from others due to shame, stigma and/or fear that their infant will be removed from their care.^429^ When inquiring about thoughts of infant harm, clinicians can provide education about their occurrence and contextualize risk to reduce barriers to disclosure. For example, clinicians can explain that thoughts about infant-related harm are common, but usually only dangerous in the context of postpartum psychosis or suicidality.

When the patient expresses intent to harm their infant, does not appear distressed by thoughts of harming the infant (i.e., “ego-syntonic” thoughts), or when the thoughts occur in individuals with psychosis or those at high risk for suicide, there is a greater risk of infant-related harm.^430^ For example, patients suffering from postpartum psychosis may believe that their infant is inhabited by an evil spirit or alien and can only be saved from this terrible situation if the parent kills the infant. A mother with severe depression and suicidal thoughts may believe their infant would suffer if the mother were to die. In this instance, the patient may genuinely believe it is better for them to include their infant in the suicide plans. When any of these situations are present, the affected mother or birthing parent should not be left alone with the infant or other children, and treatment should be sought emergently (Level 4 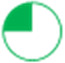). Infant-related harm can also occur spontaneously or impulsively out of extreme frustration, and clinicians should always be attentive to signs of possible child abuse and/or neglect.

## Question 10. What Are the Recommendations for Managing the Mental Health of Fathers/Co-parents?

Most research on PMADs in the non-birthing parent focuses on depression in fathers, where the prevalence of clinically significant perinatal depressive symptoms is between 8–10%.^431^ Risk factors are similar to those of maternal depression, including a history of mental illness and psychosocial stressors such as relationship distress and financial instability.^55,432^ Unemployment is an independent risk factor in this group, whereas in the maternal depression literature this appears to be of less significance.^55,432^ There is a strong correlation between maternal and paternal depression, and maternal depression is a strong risk factor for paternal depression.^55,432,433^ Anxiety has also been studied in fathers, with wide-ranging prevalence rates but an overall estimate for clinically significant anxiety symptoms of about 11% across the perinatal period.^434^ There is some suggestion that rates may increase over the course of the antenatal period, peaking in the early postpartum before decreasing thereafter.^432^ Paternal depression, and to a lesser extent paternal anxiety, is associated with a small increased risk for emotional and behavioural problems in children including after accounting for maternal mental health.^435–437^

Less is known about PMADs in non-birthing cisgender sexual minority (e.g., lesbian, bisexual, queer) or gender minority (e.g., transgender, non-binary) co-parents. There is some evidence to suggest a higher prevalence of perinatal depressive symptoms in these groups.^48,438,439^ These populations may encounter specific challenges during the transition to parenthood, including navigating heteronormative healthcare systems, minority stress and invisibility.^438–440^ Little is known about how factors such as race, ethnicity, culture and language may impact the mental health of these groups.^439^ Similarly, little is known about the prevalence of PMADs other than depression and anxiety in fathers or co-parents, although theoretically the risks of relapse could be high at this time for other disorders (including BD), given the stress of sleep deprivation and other factors in the transition to parenthood.

It is recommended that clinicians, including those who are treating the birthing parent or the child, be attentive to the mental health of the co-parent (Level 4 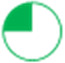). Co-parents may be reluctant to disclose mental health concerns due to a lack of knowledge about paternal/partner/co-parent mental health, fear of being judged, stigma or prioritizing the birthing parent's experience as more important.^441^ Standardized scales may be used to aid detection of depression and anxiety. The EPDS, at cut-off scores ranging from 7–10, has reasonable sensitivity/specificity (0.87/0.71 for a cut-off score of 7 to 0.71/0.86 for a cut-off score of 10) for probable depression in fathers, so can be used to aid case identification (Level 3 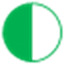), although how well this would generalize to other co-parents is not known.^442^

In systematic reviews, no RCTs focused on the treatment of depression in fathers or co-parents were identified.^443^ Interventions were generally targeted at non-clinical populations and were psychoeducational in nature, focusing on paternal well-being indirectly (i.e., via the mother or the child or the couple's relationship), with equivocal results. Systematic reviews on perinatal anxiety in fathers also evaluated mostly psychoeducational interventions in non-clinical populations.^444,445^ In some RCTs evaluating paternal anxiety, intervention groups had slightly lower levels of anxiety symptoms post-intervention than controls, but whether the interventions prevented the development of diagnosable PMADs is not known.^444^ Data on treatment for sexual and gender minority co-parents are limited but known barriers to uptake of care include a lack of targeted mental health services and supports (including peer support) specifically for—or sensitive to—the unique experiences of sexual and gender minority co-parents.^438^

## Discussion and Summary

This CANMAT guideline aims to provide clinicians with comprehensive and systematic guidance for the management of PMADs. The development of recommendations was complex at times, largely due to the gaps in available literature regarding the efficacy and safety of various treatments in the perinatal population overall and in specific subpopulations. There are also a lack of head-to-head comparisons within and across various treatment modalities (e.g., comparison of therapy and medication, or between types of therapies and types of medications). It is our hope that this guideline serves to encourage more research in the area. There is a need for better evidence in particular for conditions where there is controversy, lower-certainty and for diagnoses where there is a lack of perinatal-specific evidence such as OCD, PTSD and BD, and to a lesser extent anxiety disorders. Although there has been an attempt to improve the diagnostic criteria of certain perinatal mental health conditions, such as the “peripartum onset” specifier of the DSM-5, we encourage better acknowledgement of the unique perinatal aspects of other conditions such as anxiety, OCD, psychosis and the limitations inherent in restricting PPD onset to the first 4 weeks after childbirth. There is also a clear need for more research in specific sub-populations, including gender-diverse, sexually diverse and racialized people, and fathers and co-parents. This dynamic literature requires constant update, and it is our intent that this first CANMAT PMAD guideline can form the basis for future updates and guidance over time. We also hope that the current work will serve as a model for the development of clinical guidelines that can address other mental and behavioural disorders that were outside the scope of this guideline (e.g., autism, attention-deficit-hyperactivity disorder, eating disorders, personality disorders, substance and alcohol use disorders and others).

## Supplemental Material

sj-docx-1-cpa-10.1177_07067437241303031 - Supplemental material for Canadian Network for Mood and Anxiety Treatments 2024 Clinical Practice Guideline for the Management of Perinatal Mood, Anxiety, and Related Disorders: Guide de pratique 2024 du Canadian Network for Mood and Anxiety Treatments pour le traitement des troubles de l'humeur, des troubles anxieux et des troubles connexes périnatals Supplemental material, sj-docx-1-cpa-10.1177_07067437241303031 for Canadian Network for Mood and Anxiety Treatments 2024 Clinical Practice Guideline for the Management of Perinatal Mood, Anxiety, and Related Disorders: Guide de pratique 2024 du Canadian Network for Mood and Anxiety Treatments pour le traitement des troubles de l'humeur, des troubles anxieux et des troubles connexes périnatals by Simone N. Vigod, Benicio N. Frey, Crystal T. Clark, Sophie Grigoriadis, Lucy C. Barker, Hilary K. Brown, Jaime Charlebois, Cindy-Lee Dennis, Nichole Fairbrother, Sheryl M. Green, Nicole L. Letourneau, Tim F. Oberlander, Verinder Sharma, Daisy R. Singla, Donna E. Stewart, Patricia Tomasi, BJourn, Brittany D. Ellington, Cathleen Fleury, Lesley A. Tarasoff, Lianne M. Tomfohr-Madsen, Deborah Da Costa, Serge Beaulieu, Elisa Brietzke, Sidney H. Kennedy, Raymond W. Lam, Roumen V. Milev, Sagar V. Parikh, Arun V. Ravindran, Zainab Samaan, Ayal Schaffer, Valerie H. Taylor, Smadar V. Tourjman, Michael Van, Ameringen, Lakshmi N. Yatham and Ryan J. Van Lieshout in The Canadian Journal of Psychiatry

sj-docx-2-cpa-10.1177_07067437241303031 - Supplemental material for Canadian Network for Mood and Anxiety Treatments 2024 Clinical Practice Guideline for the Management of Perinatal Mood, Anxiety, and Related Disorders: Guide de pratique 2024 du Canadian Network for Mood and Anxiety Treatments pour le traitement des troubles de l'humeur, des troubles anxieux et des troubles connexes périnatals Supplemental material, sj-docx-2-cpa-10.1177_07067437241303031 for Canadian Network for Mood and Anxiety Treatments 2024 Clinical Practice Guideline for the Management of Perinatal Mood, Anxiety, and Related Disorders: Guide de pratique 2024 du Canadian Network for Mood and Anxiety Treatments pour le traitement des troubles de l'humeur, des troubles anxieux et des troubles connexes périnatals by Simone N. Vigod, Benicio N. Frey, Crystal T. Clark, Sophie Grigoriadis, Lucy C. Barker, Hilary K. Brown, Jaime Charlebois, Cindy-Lee Dennis, Nichole Fairbrother, Sheryl M. Green, Nicole L. Letourneau, Tim F. Oberlander, Verinder Sharma, Daisy R. Singla, Donna E. Stewart, Patricia Tomasi, BJourn, Brittany D. Ellington, Cathleen Fleury, Lesley A. Tarasoff, Lianne M. Tomfohr-Madsen, Deborah Da Costa, Serge Beaulieu, Elisa Brietzke, Sidney H. Kennedy, Raymond W. Lam, Roumen V. Milev, Sagar V. Parikh, Arun V. Ravindran, Zainab Samaan, Ayal Schaffer, Valerie H. Taylor, Smadar V. Tourjman, Michael Van, Ameringen, Lakshmi N. Yatham and Ryan J. Van Lieshout in The Canadian Journal of Psychiatry
