# Supplementary material for: Canadian Network for Mood and Anxiety Treatments 2024 Clinical Practice Guideline for the Management of Perinatal Mood, Anxiety, and Related Disorders: Guide de pratique 2024 du Canadian Network for Mood and Anxiety Treatments pour le traitement des troubles de l'humeur, des troubles anxieux et des troubles connexes périnatals
Source: Can J Psychiatry. 2025 Feb 12;70(6):429–89. doi: 10.1177/07067437241303031 (PMC11985483; doi:10.1177/07067437241303031)
Supplement: sj-docx-1-cpa-10.1177_07067437241303031 - Supplemental material for Canadian Network for Mood and Anxiety Treatments 2024 Clinical Practice Guideline for the Management of Perinatal Mood, Anxiety, and Related Disorders: Guide de pratique 2024 du Canadian Network for Mood and Anxiety Treatments pour  [file sj-docx-1-cpa-10.1177_07067437241303031.docx]

## **Supplement 1. Advisory Panels**

| Advisory Panel | Name | Details |  |
| --- | --- | --- | --- |
| Persons with lived experience | Aruna Boodram | Ontario |  |
|  | Jaimee Folkins | New Brunswick |  |
|  | Lindsay Gareau | Saskatchewan |  |
|  | Brydie Huffman | Ontario |  |
|  | Natasha MacDonald-Borgogno | Quebec |  |
|  | Naomi Mendes-Pouget | British Columbia and Ontario |  |
|  | Christine Oh | British Columbia |  |
|  | Amanda Scott | Alberta |  |
|  | Candice Thomas | Ontario |  |
|  | Christine Vanderveen | Ontario |  |
| Perinatal Care Experts | CJ Blennerhassett | Canadian Association of Midwives | |
|  | Dr. Milena Forte | Canadian College of Family Physicians | |
|  | Gabrielle Griffith | Ontario Black Doula Society | |
|  | Brittany Groom | Provincial Council for Maternal and Child Health (Ontario) | |
|  | Sheila Mills | Canadian Ass. of Perinatal and Women's Health Nurses | |
|  | Dr. Alison Shea | Society of Obstetricians and Gynaecologists of Canada | |
|  | Sarah Simpson | Social Work, McMaster University, Ontario | |
|  | Lt Colonel Dr. Andrea Tuka | Canadian Armed Forces | |
| Perinatal Mental Health Experts | Dr. Lisa Gagnon | Perinatal Psychiatry (Calgary, Alberta) | |
|  | Dr. Jasmine Gandhi | Perinatal Psychiatry Program Lead (Ottawa, Ontario) | |
|  | Dr. Tina Montreuil | Perinatal Mental Health Research (Quebec) | |
|  | Dr. Deirdre Ryan | Perinatal Psychiatry Lead (British Columbia, Yukon) | |
|  | Rami Safi | Perinatal Psychiatry Pharmacist | |
|  | Olivia Scobie | Social Work, Canadian Perinatal Mental Health Training | |
|  | Dr. Tanya Tulipan | Perinatal Psychiatry Lead (Maritime Provinces) | |
|  | Dr. Archana Vidyasankar | Perinatal Psychiatry Lead (Newfoundland) | |
|  | Dr. Andréanne Wassef | Perinatal Psychiatry (Quebec) | |
