# Supplementary material for: Canadian Network for Mood and Anxiety Treatments 2024 Clinical Practice Guideline for the Management of Perinatal Mood, Anxiety, and Related Disorders: Guide de pratique 2024 du Canadian Network for Mood and Anxiety Treatments pour le traitement des troubles de l'humeur, des troubles anxieux et des troubles connexes périnatals
Source: Can J Psychiatry. 2025 Feb 12;70(6):429–89. doi: 10.1177/07067437241303031 (PMC11985483; doi:10.1177/07067437241303031)
Supplement: sj-docx-2-cpa-10.1177_07067437241303031 - Supplemental material for Canadian Network for Mood and Anxiety Treatments 2024 Clinical Practice Guideline for the Management of Perinatal Mood, Anxiety, and Related Disorders: Guide de pratique 2024 du Canadian Network for Mood and Anxiety Treatments pour  [file sj-docx-2-cpa-10.1177_07067437241303031.docx]

## **Supplement 2. Search Strategy**

**Search Strategy Development**

The search strategy was developed for maximum sensitivity, to allow for the 10 queries identified in the project description. Following the latest CANMAT Depression Guidelines, we will focus on two concepts: (1) mood, anxiety, and related disorders, and (2) the perinatal life stage. If necessary, we will conduct additional target searches for specific interventions or specific populations to address gaps in the results.

Search terms were drawn from the project description, from related reviews, and from the MeSH subject headings and the related terms. The Mesh terms were searched separately from keywords, and the highest-level MeSH term was exploded. Truncation and wildcards were used to reduce repetition in the search string and to increase sensitivity.

| Concept 1 | Concept 2 |
| --- | --- |
| Mood and Anxiety related terms (mental health).  E.g.: mood disorders, baby blues, bipolar disorder... | Maternal or non-birth parent (life stage),  E.g.: puerperal, perinatal, postpartum... |

**Filters**

- Results are limited to English and French.
- Results are filtered to dates from January 2013 to current, and Human.
- Results are limited to Guidelines, Meta Analyses, and Systematic Reviews.

We are searching the following databases: PubMed, EMBASE, PsycINFO, MEDLINE, Cochrane, CINAHL, and Web of Science. While the overall literature search strategy will remain consistent, please note that the subject headings used may differ from one database to another.

Searches were conducted from October 26 to 29, 2023.  All results were uploaded to Covidence for screening and extraction.

**Testing**

1. A test of sensitivity was done to determine if all sub-categories of anxiety were being captured by the MeSH and Keyword searches. A separate search was done with all indicated anxiety terms, and none had been missed in the search strategy.
2. An additional test was done to determine if including psychotherapy MeSH terms would yield relevant terms. Only 32 additional citations were retrieved; none met the inclusion criteria.
3. A test of specificity was done by searching signal articles (systematic reviews) provided by members of the core editorial board that met the search criteria (date, publication type, mental disorders and life stage). All five systematic review citations (in addition to one meta-analysis) tested appeared in the search set.

**Signal articles:**

Kleine, I. (2020). Interventions to prevent perinatal depression: us preventive services task force recommendation statement. Archives of Disease in Childhood Education & Practice, 105(4), 242-243. <https://dx.doi.org/10.1136/archdischild-2019-317433>

Fawcett, E. J., Fairbrother, N., Cox, M. L., White, I. R., & Fawcett, J. M. (2019). The prevalence of anxiety disorders during pregnancy and the postpartum period: a multivariate bayesian meta-analysis. Journal of Clinical Psychiatry, 80(4), 18r12527. <https://doi.org/10.4088/JCP.18r12527>

Moore Simas, T. A., Flynn, M. P., Kroll-Desrosiers, A. R., Carvalho, S. M., Levin, L. L., Biebel, K., Byatt, N. (2018). A systematic review of integrated care interventions addressing perinatal depression care in ambulatory obstetric care settings. Clinical Obstetrics & Gynecology, 61(3), 573-590. <https://dx.doi.org/10.1097/GRF.0000000000000360>

Nillni, Y. I., Mehralizade, A., Mayer, L., Milanovic, S. (2018). Treatment of depression, anxiety, and trauma-related disorders during the perinatal period: a systematic review. Clinical Psychology Review, 66, 136-148. <https://dx.doi.org/10.1016/j.cpr.2018.06.004>

Singla, D. R., Lawson, A., Kohrt, B. A., Jung, J. W., Meng, Z., Ratjen, C., Zahedi, N., Dennis, C. L., Patel, V. (2021). Implementation and effectiveness of nonspecialist-delivered interventions for perinatal mental health in high-income countries: a systematic review and meta-analysis. JAMA Psychiatry, 78(5), 498-509. <https://dx.doi.org/10.1001/jamapsychiatry.2020.4556>

Wang, T. H., Tzeng, Y. L., Teng, Y. K., Pai, L. W., Yeh, T. P. (2022). Evaluation of psychological training for nurses and midwives to optimise care for women with perinatal depression: a systematic review and meta-analysis. Midwifery, 104, 103160. <https://dx.doi.org/10.1016/j.midw.2021.103160>

**Ovid Medline: Epub ahead of print, in-process & other non-indexed citations, Ovid Medline Daily and Ovid Medline 1946-present**

**Searched 26 October 2023**

|  | ***Search*** | ***Results*** |
| --- | --- | --- |
| 1 | exp Anxiety Disorders/ or exp bipolar disorder/ or exp Depressive disorder/ or exp mental disorders/ or exp Mental health/ or exp Mood disorders/ or exp depression, postpartum/ or exp puerperal disorders/ or exp stress disorders, post traumatic/ | 1,517,202 |
| 2 | ((Affect* adj2 (Disorder* or disturb* or psychos*)) or agitat* or Anankastic or angst or Anxiet* or anxious* or Baby blue* or baby pink* or Bipolar or child$parent relation* or compulsion* or (compuls* adj2 neuros*s) or cyclothymic or depress* or (fear adj2 child$birth) or GAD or (manic* adj2 (depress* or Disorder* or psycho*)) or (Mood adj2 (Disorder* or disregulat*)) or Moral Injur* or Neuros*s or Neurotic* or obessive$compulsive or Obsession* or panic or phobi* or PMAD or ((puerper* or Post$delivery or post$natal or post$partum) adj2 (Psycho* or depress* or dysphoria or blues)) or (Post$Traumatic adj2 (stress* or Neuros*s)) or PPD or PTSD or (season* adj2 affect*) or tokophobia or trauma* or (traumatic adj2 stress*)).ab,kf,kw,ti. | 1,320,092 |
| 3 | exp Parturition/ or exp Maternal-fetal relations/ or exp paternal behavior/ or exp stillbirth/ or exp prenatal care/ or exp pregnancy/ or exp pregnancy complications/ or exp Postpartum Period/ or exp Peripartum Period/ or exp breast feeding/ or exp adoption/ or exp surrogate mothers/ | 1,108,019 |
| 4 | ("After birth" or ante*natal or Birth* or non*birth or adopt* or surrogate or breast*feed* or Child*birth* or expectant or "Fourth trimester" or Gestation* or Labor or Matern* or Mother* or Parturit* or paternal or Peri*natal or Peri*partum or Post*birth or Post*delivery or post*natal or Post*partum or pre*natal or Pre*conception or pregnan* or Puerper* or Still*birth or patern* or father or non$birth* or same$sex).ab,kf,kw,ti. | 1,860,357 |
| 5 | 1 or 2 | 2,481,098 |
| 6 | 3 or 4 | 2,207,785 |
| 7 | 5 and 6 | 199,537 |
| 8 | protocol.ti. | 80,782 |
| 9 | 7 not 8 | 198,297 |
| 10 | limit 10 to (yr="2013 - 2024" and (english or french) and (guideline or meta analysis or practice guideline or "systematic review")) | 3,992 |

**APA PsychInfo, 1806 to October week 3 2023**

**Searched 27 October 2023**

|  | ***Search*** | ***Results*** |
| --- | --- | --- |
| 1 | exp Perinatal period/ or exp Pregnancy/ or exp Birth/ or exp Postnatal period/ or exp Antepartum period/ or exp Intrapartum period/ or exp Postnatal period/ or exp prenatal care/ or exp breast feeding/ or exp lactation/ or exp birth injuries/ or exp birth trauma/ or exp obstetrical complications/ or exp labor/ or exp pregnancy outcomes/ or exp adoption child/ or exp adoptive parents/ | 68,465 |
| 2 | ("After birth" or ante*natal or Birth* or breast*feed* or Child*birth* or expectant or "Fourth trimester" or Gestation* or Matern* or "Mother* ADJ2 new" or parturit* or paternal or Peri*natal or Peri*partum or Post*birth or Post*delivery or post*natal or Post*partum or pre*natal or Pre*conception or pregnan* or Puerper* or Still*birth or gestation or surrogate or adopt*).ab,id,ti. | 314,161 |
| 3 | 1 or 2 | 319,195 |
| 4 | ((exp affective disorders/ or exp postpartum depression/ or exp affective psychosis/ or exp anhedonia/ or exp amnesia/ or exp anxiety disorders/ or exp Atypical Depression/ or exp bipolar disorder/ or exp depression emotion/ or exp dissociation/ or exp euthymia/ or exp generalized anxiety disorder/ or exp gender dysphoria/ or exp hysteria/ or exp illness anxiety disorder/ or exp Major Depression/ or exp neurosis/ or exp obsessive compulsive disorder/ or exp panic disorder/ or exp phobias/ or exp postpartum psychosis/ or exp reactive depression/ or exp Recurrent Depression/ or exp schizoaffective disorder/ or exp social anxiety/ or exp stress/ or exp stress/) and trauma related disorders/) or exp stress reactions/ | 40,183 |
| 5 | ("Affect* ADJ2 Disorder*" or "affect* ADJ2 disturb*" or "Affect* ADJ2 Psychos*s" or agitat* or Anankastic or angst or anhedonia or Anxiet* or anxious* or "Baby blue*" or "baby pink*" or Bipolar or "child*parent relation*" or compulsion* or "compulsive ADJ3 neuros*s" or crisis or cyclothymic or depress* or dysphoria or euthymi* or "fear ADJ3 childbirth" or GAD or "mani* AJ2 depress*" or "Manic ADJ2 Disorder*" or "manic* ADJ2 psycho*" or "manic*depress Adj2 Psycho*" or melancholia or "Mood ADJ2 Disorder*" or "mood ADJ2 disregulat*" or "Moral Injur*" or "Moral Manic Depress*" or LGBTQ or neurocirculat* or Neuros*s or Neurotic* or obessive*compulsive or Obsession* or orthorexia or panic or phobi* or PMAD or "Post*delivery ADJ2 Psycho*" or "Post*natal ADJ2 Depress*" or "post*natal ADJ2 dysphoria*" or "post*natal ADJ2 blues" or "Post*partum ADJ2 Depress*" or "post*partum ADJ2 psycho*" or "Post*partum ADJ2 Blues" or "Post*Traumatic ADJ2 Neuros*s" or "Post*traumatic ADJ2 Stress*" or PPD or PTSD or Psychoneurosis or "Puerper* ADJ2 Psycho*" or "season* ADJ2 affect*" or tokophobia or trauma* or "traumatic ADJ2 stress*").ab,id,ti. | 754,794 |
| 6 | 4 or 5 | 784,402 |
| 7 | 3 and 6 | 52,994 |
| 8 | limit 7 to (("0830 systematic review" or 1200 meta analysis or 1300 metasynthesis) and (english or french) and yr="2013 -Current") | 1,042 |

**Embase Classic + Embase, 1947 to 2023 October 27**

**Searched 28 October 2023**

|  | ***Search*** | ***Results*** |
| --- | --- | --- |
| 1 | exp antenatal depression/ or exp depression/ or exp postnatal depression/ or exp prenatal depression/ or exp recurrent brief depression/ or exp mood disorder/ or exp fear of childbirth/ or exp anxiety/ or exp neurosis/ or exp posttraumatic stress disorder/ or exp phobia/ | 969,200 |
| 2 | ("Affect* ADJ2 Disorder*" or "affect* ADJ2 disturb*" or "Affect* ADJ2 Psychos*s" or agitat* or Anankastic or "ADJ2 Personalit*" or angst or anhedonia or Anxiet* or anxious* or "Baby blue*" or "baby pink*" or Bipolar or "child*parent relation*" or compulsion* or "compulsive ADJ3 neuros*s" or crisis or cyclothymic or depress* or dysphoria or euthymi* or "fear ADJ3 childbirth" or "Manic ADJ2 Disorder*" or "manic* ADJ2 psycho*" or "manic*depress Adj2 Psycho*" or melancholy* or "Mood ADJ2 Disorder*" or "mood ADJ2 disregulat*" or "Moral Injur*" or "Moral Manic Depress*" or Neuros*s or Neurotic* or obessive*compulsive or Obsession* or orthorexia or panic or phobi* or PMAD or "Post*delivery ADJ2 Psycho*" or "post*natal ADJ2 blues" or "post*partum ADJ2 psycho*" or "Post*partum ADJ2 Blues" or "Post*Traumatic ADJ2 Neuros*s" or "Post*traumatic ADJ2 Stress*" or PPD or PTSD or Psychoneurosis or "Puerper* ADJ2 Psycho*" or "season* ADJ2 affect*" or tokophobia or trauma* or "traumatic ADJ2 stress*").ab,hw,kf,ti. | 2,245,178 |
| 3 | 1 or 2 | 2,293,308 |
| 4 | exp child parent relation/ or exp expectant parent/ or exp maternal care/ or exp parent/ or exp paternal behavior/ or exp perinatal care/ or exp perinatal period/ or exp postnatal care/ or exp pregnancy/ or exp pregnancy care/ or exp prenatal care/ or exp prenatal period/ or exp birth/ or exp stillbirth/ or exp adoption/ or exp surrogate mother/ | 1,514,413 |
| 5 | ("After birth" or ante*natal or Birth* or breast*feed* or Child*birth* or expectant or "Fourth trimester" or Gestation* or Labor or Matern* or "Mother* ADJ2 new" or "father* ADJ2 new" or Parturit* or paternal* or Peri*natal or Peri*partum or Post*birth or Post*delivery or post*natal or Post*partum or pre*natal Pre*conception or pregnan* or adopt* or Puerper* or Still*birth).ab,hw,kf,ti. | 2,572,583 |
| 6 | 4 or 5 | 2,842,723 |
| 7 | 3 and 6 | 188,736 |
| 8 | limit 7 to ((meta analysis or "systematic review") and (english or french) and yr="2013 -Current") | 5,168 |

**EBSCO CINAHL Complete**

**Searched 29 October 2023**

|  | Search | Results |
| --- | --- | --- |
| 1 | “Pregnancy Complications” OR “Depression, Postpartum” OR “Postpartum Psychosis” OR “Behavioral and Mental Disorders” OR “Mental Disorders” OR “Affective Disorders, Psychotic” OR “Bipolar Disorder” OR “Cyclothymic Disorder” OR “Neurotic Disorders” OR “Affective Disorders” OR “Depression, Postpartum” OR “Female Urogenital Diseases and Pregnancy Complications” | 133,057 |
| 2 | “Affect* N2 Disorder*” OR “affect* N2 disturb*” OR “Affect* N2 Psychos*s” OR agitat* OR Anankastic OR angst OR anhedonia OR Anxiet* OR anxious* OR “Baby blue*” OR “baby pink*” OR Bipolar OR “child*parent relation*” OR compulsion* OR “compulsive N3 neuros*s OR crisis OR cyclothymic OR depress* OR dysphoria OR euthymia OR “fear N3 childbirth” OR GAD “mani* N2 depress*” OR “Manic N2 Disorder*” OR “manic* ADJ2 psycho*” OR melancholy* OR “Mood N2 Disorder*” OR “mood N2 disregulat*” OR “Moral Injur*” OR “Moral Manic Depress*” OR LGBTQ* OR Neuros*s OR Neurotic* OR obessive*compulsive OR Obsession* OR orthorex* OR panic OR phobi* OR PMAD OR “Post*delivery N2 Psycho*” OR “Post*natal N2 Depress*” OR “post*natal N2 dysphoria*” OR “post*natal N2 blues” OR “Post*partum N2 Depress*” OR “post*partum N2 psycho*” OR “Post*partum N2 Blues” OR “Post*Traumatic N2 Neuros*s” OR “Post*traumatic ADJ2 Stress*” OR PPD OR PTSD OR Psychoneuro* OR “Puerper* N2 Psycho*” OR “season* N2 affect*” OR tokophobia* OR trauma* OR “traumatic N2 stress*” | 94,068 |
| 3 | 1 OR 2 | 206,471 |
| 4 | (MH "Expectant Mothers") OR (MH "Pregnancy Outcomes") OR (MH "Labor Complications") | 43,915 |
| 5 | “Postnatal Period” OR Puerperium OR Pregnancy OR “Perinatal Period” OR Childbirth OR Labor | 289,685 |
| 6 | “After birth” OR ante*natal OR Birth* OR breast*feed* OR Child*birth* OR expectant OR “Fourth trimester” OR Gestation* OR Labor OR Matern* OR Mother* OR Parturit* OR paternal OR Peri*natal OR Peri*partum OR Post*birth OR Post*delivery OR post*natal OR Post*partum OR pre*natal OR Pre*conception OR pregnan* OR Puerper* OR Still*birth OR adopt* | 641,375 |
| 7 | 4 OR 5 OR 6 | 641,375 |
| 8 | TI meta*analysis OR "systematic review" OR guideline* | 1,256 |
| 9 | 3 AND 7  **Limiters** - Published Date: 20130101-20231131; Publication Type: Meta Analysis, Meta Synthesis, Practice Guidelines, Systematic Review; Language: English, French | 1,254 |

**Web of Science**

**Searched 28 October 2023**

|  | Search | Results |
| --- | --- | --- |
|  | TS=(Affect* OR agitat* OR Anankastic OR angst OR anhedonia OR Anxiet* OR anxious* OR “Baby blue*” OR “baby pink*” OR Bipolar OR compulsion* OR compulsive OR crisis OR cyclothymic OR depress* OR dysphoria OR euthymia OR gad OR mania* OR manic* OR melancholia OR mood OR “Moral Injur*” OR neurocirculat* OR Neuros*s OR Neurotic* OR obessive*compulsive OR Obsession* OR orthorexia OR panic OR phobi* OR pmad OR ppd OR ptsd OR Psychoneurosis OR tokophobia OR trauma* OR stress) |  |
|  | TS=(After*birth OR ante*natal OR Birth* OR breast*feed* OR Child*birth* OR child*parent OR expectant OR “Fourth trimester” OR Gestation* OR Labo*r OR Matern* OR Mother* OR Parturit* OR paternal OR Peri*natal OR Peri*partum OR Post*birth OR Post*delivery OR post*natal OR Post*partum OR pre*natal OR Pre*conception OR pre*natal OR pregnan* OR Puerper* OR Still*birth OR adopt*) |  |
|  | TI=(meta*analysis OR "systematic review" or guideline*) and English or French (Languages) | 6,152 |

**Cochrane Library**

**Searched 29 October 2023**

| 1 | MeSH descriptor: [Anxiety Disorders] explode all trees | 9,179 |
| --- | --- | --- |
| 2 | MeSH descriptor: [Bipolar Disorder] explode all trees | 3,632 |
| 3 | MeSH descriptor: [Depressive Disorder] explode all trees | 15,284 |
| 4 | MeSH descriptor: [Mood Disorders] explode all trees | 18,949 |
| 5 | MeSH descriptor: [Stress Disorders, Post-Traumatic] | 3,701 |
| 6 | #1 OR #2 OR #3 OR #4 OR #5 | 29,908 |
| 7 | agitat* OR Anankastic OR angst OR Anxiet* OR anxious* OR Bipolar OR compulsion* OR GAD OR cyclothymic OR depress* OR Neuros*s OR Neurotic* OR panic OR phobi* OR PMAD OR PPD OR PTSD  OR Obsession* OR tokophobia OR trauma* OR Baby NEXT blue* OR baby NEXT pink* OR child$parent NEXT relation* OR compuls* NEAR/2 neuros*s OR fear NEAR/2 child$birth OR Moral NEXT Injur* OR obessive NEXT compulsive OR season* NEAR/2 affect* OR traumatic NEAR/2 stress* | 209,310 |
| 8 | manic* NEAR/2 (depress* or Disorder* or psycho*) | 781 |
| 9 | Mood NEAR/2 (Disorder* or disregulat*) 4817 | 4,817 |
| 10 | (puerper* OR Post$delivery OR post$natal OR post$partum) NEAR/2 (Psycho* OR depress* OR dysphoria OR blues) | 473 |
| 11 | Post$Traumatic NEAR/2 (stress* or Neuros*s) | 5,815 |
| 12 | #7 OR #8 OR #9 OR #10 OR #11 | 210,313 |
| 13 | #6 OR #12 | 210,722 |
| 14 | After NEXT birth OR ante*natal OR Birth* OR non*birth OR adopt* OR surrogate OR breast*feed* OR Child*birth* OR expectant OR Fourth NEXT trimester OR Gestation* OR Labor OR Matern* OR Mother* OR Parturit* OR paternal OR Peri*natal OR Peri*partum OR Post*birth OR Post*delivery OR post*natal OR Post*partum OR pre*natal OR Pre*conception | 161,943 |
| 15 | MeSH descriptor: [Parturition] explode all trees | 741 |
| 16 | MeSH descriptor: [Maternal-Fetal Relations] explode all trees | 25 |
| 17 | MeSH descriptor: [Paternal Behavior] explode all trees | 41 |
| 18 | MeSH descriptor: [Stillbirth] explode all trees | 278 |
| 19 | MeSH descriptor: [Prenatal Care] explode all trees | 2,140 |
| 20 | MeSH descriptor: [Pregnancy] explode all trees | 31,498 |
| 21 | MeSH descriptor: [Pregnancy Complications] explode all trees | 16,176 |
| 22 | MeSH descriptor: [Postpartum Period] explode all trees | 2,478 |
| 23 | MeSH descriptor: [Peripartum Period] explode all trees | 36 |
| 24 | MeSH descriptor: [Breast Feeding] explode all trees | 2,677 |
| 25 | MeSH descriptor: [Adoption] explode all trees | 73 |
| 26 | MeSH descriptor: [Surrogate Mothers] explode all trees | 3 |
| 27 | #14 OR #15 OR #16 OR #17 OR #18 OR #19 OR #20 OR #21 OR #22 OR #23 OR #24 OR #25 OR #26 | 162,808 |
| 28 | #13 AND #27 with Cochrane Library publication date Between Jan 2013 and Oct 2023, in Cochrane Reviews | 2,478 |

Additional Focused Searches were conducted for medical therapies and for screening for bipolar disorder, as these had been identified as gaps.  The search was not limited to and for systematic reviews or meta-analyses, so the Cochrane Database was not searched.  Otherwise, the same databases and terms relating to the population and to perinatal terms were searched.

**Focused Search: Screening for Bipolar Disorder**

**Ovid MEDLINE(R) ALL, 1946 to March 29, 2024**

**Searched 31 March 31**

|  | Search | Results |
| --- | --- | --- |
| 1 | exp Parturition/ or exp Maternal-fetal relations/ or exp paternal behavior/ or exp stillbirth/ or exp prenatal care/ or exp pregnancy/ or exp pregnancy complications/ or exp Postpartum Period/ or exp Peripartum Period/ or exp breast feeding/ or exp adoption/ or exp surrogate mothers/ | 1,123,244 |
| 2 | ("After birth" or ante*natal or Birth* or non*birth or adopt* or surrogate or breast*feed* or Child*birth* or expectant or "Fourth trimester" or Gestation* or Labor or Matern* or Mother* or Parturit* or paternal or Peri*natal or Peri*partum or Post*birth or Post*delivery or post*natal or Post*partum or pre*natal or Pre*conception or pregnan* or Puerper* or Still*birth or patern* or father or non$birth* or same$sex).ab,kf,kw,ti | 1,904,460 |
| 3 | 1 or 2 | 2,253,982 |
| 4 | exp bipolar disorder/ | 45,950 |
| 5 | Bipolar.ti,ab,kf,kw | 77,049 |
| 6 | 4 or 5 | 93,482 |
| 7 | mass screening/ | 117,420 |
| 8 | (questionnaire* or screen* or scale* or tool*).ab,kf,kw,ti | 3,478,542 |
| 9 | 7 or 8 | 3,505,676 |
| 10 | (psych* or mental).ti,ab,kf,kw | 1,408,490 |
| 11 | 9 and 10 | 377,572 |
| 12 | exp psychiatric status rating scales/ | 87,351 |
| 13 | 11 or 12 | 438,818 |
| 14 | 3 and 6 and 13 | 599 |
| 15 | limit 14 to (yr="2013 -Current" and (english or french) and (clinical study or clinical trial, all or clinical trial, phase i or clinical trial, phase ii or clinical trial, phase iii or clinical trial, phase iv or clinical trial or controlled clinical trial or journal article or observational study or pragmatic clinical trial or randomized controlled trial or research support, nih, extramural or research support, nih, intramural or research support, non us gov't or research support, us gov't, non phs or research support, us gov't, phs or "review")) | 349 |
| 16 | limit 15 to humans | 290 |

**APA PsychInfo, 1806 to March Week 4 2024**

**Searched 31 March 2024**

|  | ***Search*** | ***Results*** |
| --- | --- | --- |
| 1 | exp Parturition/ or exp Maternal-fetal relations/ or exp paternal behavior/ or exp stillbirth/ or exp prenatal care/ or exp pregnancy/ or exp pregnancy complications/ or exp Postpartum Period/ or exp Peripartum Period/ or exp breast feeding/ or exp adoption/ or exp surrogate mothers/ | 61174 |
| 2 | exp bipolar disorder/ | 35436 |
| 3 | ("After birth" or ante*natal or Birth* or breast*feed* or Child*birth* or expectant or "Fourth trimester" or Gestation* or Matern* or "Mother* ADJ2 new" or parturit* or paternal or Peri*natal or Peri*partum or Post*birth or Post*delivery or post*natal or Post*partum or pre*natal or Pre*conception or pregnan* or Puerper* or Still*birth or gestation or surrogate or adopt*).ab,id,ti | 321155 |
| 4 | bipolar.ab,id,ti | 46511 |
| 5 | (questionnaire* or screen* or scale* or tool*).ab,id,ti | 934850 |
| 6 | (psych* or mental).ti,ab,id | 1691483 |
| 7 | 1 or 3 | 325578 |
| 8 | 2 or 4 | 51522 |
| 9 | exp Rating Scales/ | 26187 |
| 10 | exp Screening/ or exp Screening Tests/ | 19774 |
| 11 | 5 or 9 or 10 | 936609 |
| 12 | 7 and 8 and 11 | 458 |
| 13 | limit 12 to (human and ("0300 clinical trial" or "0430 followup study" or "0450 longitudinal study" or "0451 prospective study" or "0453 retrospective study" or "0600 field study" or "0700 interview" or "0750 focus group" or 1600 qualitative study or 1800 quantitative study) and (english or french)) | 200 |

**Embase Classic + Embase, 1947 to 2024 March 29**

**Searched 31 March 2024**

|  | ***Search*** | ***Results*** |
| --- | --- | --- |
| 1 | exp Parturition/ or exp Maternal-fetal relations/ or exp paternal behavior/ or exp stillbirth/ or exp prenatal care/ or exp pregnancy/ or exp pregnancy complications/ or exp Postpartum Period/ or exp Peripartum Period/ or exp breast feeding/ or exp adoption/ or exp surrogate mothers/ | 1,509,632 |
| 2 | ("After birth" or ante*natal or Birth* or non*birth or adopt* or surrogate or breast*feed* or Child*birth* or expectant or "Fourth trimester" or Gestation* or Labor or Matern* or Mother* or Parturit* or paternal or Peri*natal or Peri*partum or Post*birth or Post*delivery or post*natal or Post*partum or pre*natal or Pre*conception or pregnan* or Puerper* or Still*birth or patern* or father or non$birth* or same$sex).ab,kf,kw,ti | 2,526,179 |
| 3 | 1 or 2 | 2,947,977 |
| 4 | exp bipolar disorder/ | 84,867 |
| 5 | Bipolar.ti,ab,kf,kw | 115,799 |
| 6 | 4 or 5 | 142,915 |
| 7 | mass screening/ | 68,230 |
| 8 | (questionnaire* or screen* or scale* or tool*).ab,kf,kw,ti | 4,713,334 |
| 9 | 7 or 8 | 4,733,572 |
| 10 | (psych* or mental).ti,ab,kf,kw | 1,905,971 |
| 11 | 9 and 10 | 543,095 |
| 12 | exp psychiatric status rating scales/ | 22,856 |
| 13 | 11 or 12 | 557,310 |
| 14 | 3 and 6 and 13 | 1,133 |
| 15 | limit 14 to (human and (clinical trial or randomized controlled trial or controlled clinical trial or multicenter study or phase 1 clinical trial or phase 2 clinical trial or phase 3 clinical trial or phase 4 clinical trial) and (english or french) and yr="2013 -Current" and (article or "review")) | 35 |

**EBSCO CINAHL Complete**

**Searched Sunday, March 31, 2024**

|  | Search | Results |
| --- | --- | --- |
| 1 | (MH "Expectant Mothers")OR (MH "Pregnancy Outcomes") OR (MH "Labor Complications") | 45,319 |
| 2 | “After birth” OR ante*natal OR Birth* OR breast*feed*OR Child*birth* OR expectant OR “Fourth trimester” OR Gestation*OR Labor OR Matern* OR Mother* OR Parturit* OR paternal OR Peri*natal OR Peri*partum OR Post*birth OR Post*delivery OR post*natal OR Post*partum OR pre*natal OR Pre*conception OR pregnan* OR Puerper*OR Still*birth OR adopt* | 651,099 |
| 3 | (MH "Bipolar Disorder") | 13,698 |
| 4 | ""“Postnatal Period” OR Puerperium OR Pregnancy OR “Perinatal Period” OR Childbirth OR Labor"" | 361,675 |
| 5 | "bipolar" | 22,011 |
| 6 | S1 OR S2 OR S4 | 651,099 |
| 7 | S3 OR S5 | 22,011 |
| 8 | SU health screening | 56,939 |
| 9 | TI (tool OR scale OR questionnaire or rating) | 101,911 |
| 10 | S8 OR S9 | 156,248 |
| 11 | S6 AND S7 AND S10 | 28 |
| 12 | S6 AND S7 AND S10  Limiters  -PublicationDate: 20130101-20241231 | 18 |

**
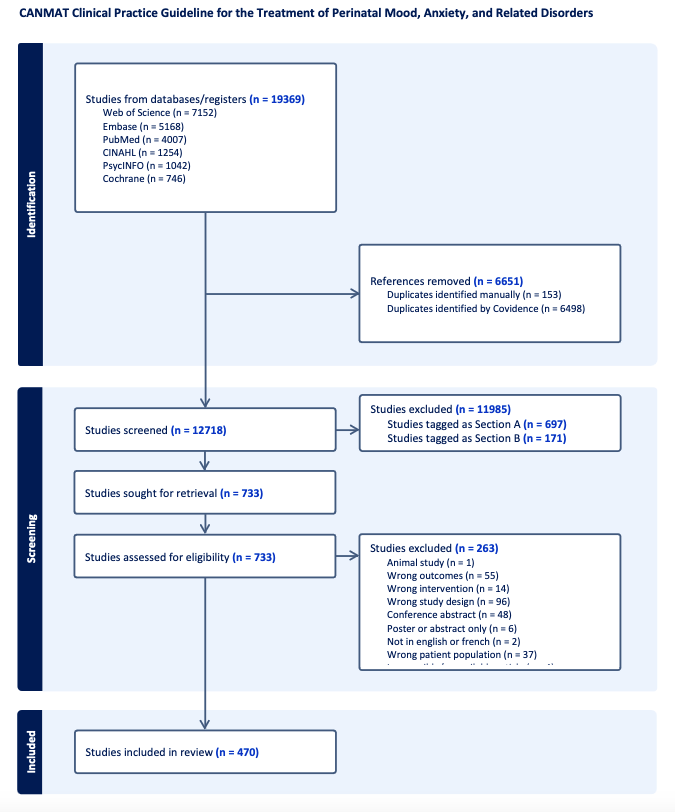
**
